# PROTACs: great opportunities for academia and industry (an update from 2020 to 2021)

**DOI:** 10.1038/s41392-022-00999-9

**Published:** 2022-06-09

**Authors:** Ming He, Chaoguo Cao, Zhihao Ni, Yongbo Liu, Peilu Song, Shuang Hao, Yuna He, Xiuyun Sun, Yu Rao

**Affiliations:** 1 Ministry of Education (MOE) Key Laboratory of Protein Sciences, School of Pharmaceutical Sciences, MOE Key Laboratory of Bioorganic Phosphorus Chemistry & Chemical Biology, Tsinghua University, 100084 Beijing, P. R. China; 2 Tsinghua-Peking Center for Life Sciences, 100084 Beijing, P. R. China; 3 School of Pharmaceutical Sciences, Zhengzhou University, 450001 Zhengzhou, China

**Keywords:** Chemical biology, Drug discovery

## Abstract

PROteolysis TArgeting Chimeras (PROTACs) technology is a new protein-degradation strategy that has emerged in recent years. It uses bifunctional small molecules to induce the ubiquitination and degradation of target proteins through the ubiquitin–proteasome system. PROTACs can not only be used as potential clinical treatments for diseases such as cancer, immune disorders, viral infections, and neurodegenerative diseases, but also provide unique chemical knockdown tools for biological research in a catalytic, reversible, and rapid manner. In 2019, our group published a review article “PROTACs: great opportunities for academia and industry” in the journal, summarizing the representative compounds of PROTACs reported before the end of 2019. In the past 2 years, the entire field of protein degradation has experienced rapid development, including not only a large increase in the number of research papers on protein-degradation technology but also a rapid increase in the number of small-molecule degraders that have entered the clinical and will enter the clinical stage. In addition to PROTAC and molecular glue technology, other new degradation technologies are also developing rapidly. In this article, we mainly summarize and review the representative PROTACs of related targets published in 2020–2021 to present to researchers the exciting developments in the field of protein degradation. The problems that need to be solved in this field will also be briefly introduced.

## Introduction

**Fig. 1:**
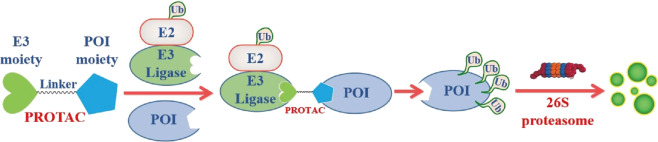
The mechanism of PROTAC-mediated protein degradation

**Fig. 2:**
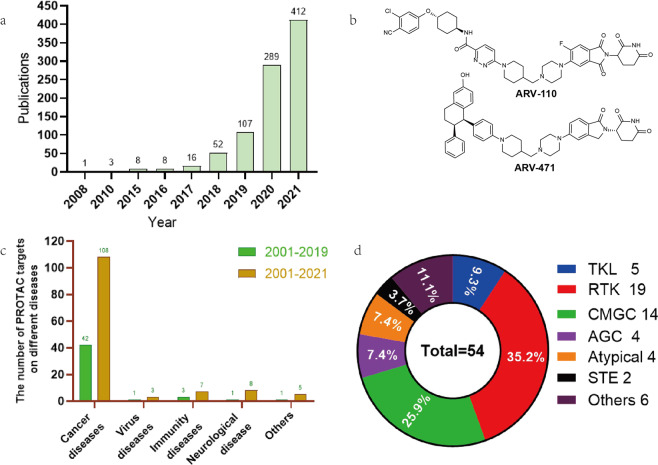
The researches on PROTAC from 2001 to 2021. **a** The publications on PROTACs from 2001 to 2021. **b** The structure of **ARV-110** and **ARV-471**. **c** The comparison of PROTAC targets on different diseases between 2001–2019 and 2001–2021. **d** Classification and percentage of degradable kinases

In 2001, Crews group and Deshaies group reported the first example of PROTACs.^1^ As a novel chemical biology technology, PROTACs present a chemical knockdown strategy by hijacking the ubiquitin–proteasome system with bifunctional small molecules that can simultaneously bind target protein and E3 ubiquitin ligase and induce the target protein to be ubiquitylated and then be degraded by proteasome (Fig. 1). In the past 20 years, especially since **dBET1** PROTAC based on pomalidomide as the E3 ligase ligand successfully degraded BET protein in 2015,^2^ the field of PROTACs has ushered in a period of rapid development (Fig. 2a). So far, a variety of PROTACs derived from different E3 ligase and protein ligands have been disclosed to achieve the degradation of various types of interesting proteins.

Due to its unique mode of action, PROTACs technology has received great attention in the industry and has been applied to the possible treatment of cancers, immune disorders, viral infections, neurodegenerative diseases, etc. Arivinas, a clinical-stage biopharmaceutical company, recently disclosed the structures of the androgen receptor (AR) degrader **ARV-110** and the estrogen-receptor (ER) degrader **ARV-471** (Fig. 2b).^3–5^ The previously announced results indicate that **ARV-110** is safe and effective for patients with metastatic castration-resistant prostate cancer(mCRPC). This is PROTAC’s first clinical trial data and presents a milestone in the transformation of PROTAC technology into a new treatment strategy. As the first targeted estrogen-receptor-degrading agent to enter clinical trials, **ARV-471** is another potential best-in-class drug that may bring hope to breast cancer patients. Besides the AR and ER targeting PROTAC molecules, more and more PROTAC degraders have entered the clinical trial stage in the past 2 years. For instance, the new targets of these PROTAC molecules include BCL-xL, IRAK4, STAT3, BTK, BRD9, MDM2, etc., among which most are the first-in-class targets. In addition to being used as possible clinical treatments, PROTAC is an efficient protein knockdown tool that can directly control protein levels without gene editing operations. It can be used as a useful supplement to existing genetic research tools and provide possible answers to many fundamental biological questions. With the in-depth understanding of the mechanism of PROTAC and its great potential in biological research and disease treatment, more and more researchers have begun to pay attention to this field, and more targets have been proven to be degradable by PROTAC molecules. In 2019, we wrote a review of PROTACs in this journal,^6^ summarizing that there were about 40 proteins that could be degraded at that time. According to the latest statistics in December 2021, the reported PROTAC targets have reached more than 130 (Fig. 2c). The number of degradable targets reported in 2020–2021 (about 90) has completely exceeded the total amount of the previous 18 years, indicating that the era of protein degradation has arrived.

In the reported studies, researchers are more inclined to choose kinases as the targets of protein degradation. According to incomplete statistics, about 54 kinases can be degraded by different degraders based on the PROTAC technology, accounting for 45% of the total targets (Fig. 2d). The main reason is that most kinases have known and effective inhibitors or ligands, which can be easily modified to connect linkers and maintain sufficient binding affinity. In addition, the kinase has a deep binding pocket, which can promote the binding of PROTACs, thereby inducing the interaction between the kinase and the E3 ligase, and then ubiquitinating and finally degrading the kinase. Moreover, although the kinase protein has a high degree of homology, PROTAC can selectively degrade different subtypes of kinases and can even be developed from nonselective inhibitors. How is the selectivity achieved by PROTAC? Nonselective inhibitors will target the highly conserved ATP-binding pocket to exert their inhibitory effects. As is known, the residues in the ATP-binding pocket of different subtypes of kinases are highly similar, which usually results in poor selectivity for these inhibitors. When nonselective inhibitors serve as POI ligands for PROTACs, not only the protein ligands can recognize the corresponding kinases, but the degraders can induce specific protein–protein interaction between POI and E3 ligase to form a ternary complex. The two-step recognition mechanisms can lead to selectively degradation of targets.

**Fig. 3:**
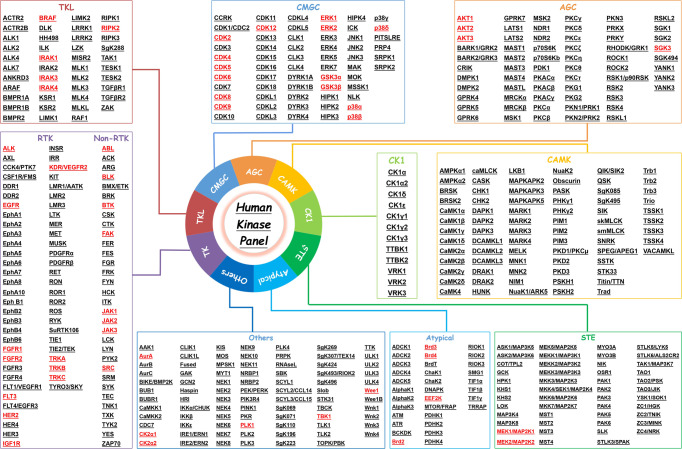
The list of human “degradable” kinases based on PROTAC

There are 518 kinds of kinases that have been discovered so far, which are involved in a variety of physiological regulatory processes such as cell survival, proliferation, differentiation, apoptosis, and metabolism.^7^ These kinases have been divided into nine categories based on their structure and function, namely, tyrosine kinases (RTKs), TKL kinases (TKLs), STE kinases (STEs), CAMK kinases (CAMKs), and AGC kinases (AGCs)), CMGC kinase group (CMGCs), atypical protein kinase group (atypical), CK1 kinase group, and other groups (others). According to the classification of the human kinase profile, we have marked the kinases that can be degraded as degradable kinases (Fig. 3). In the “degradable” kinase table, according to the classification method of the kinase profile, it was noticed that only CK1 and CAMK have no reported PROTACs. The first and second targets are RTKs and CMGCs, with 19 (35.2%) and 14(25.9%) targets, respectively, accounting for more than half of the total kinases (Fig. 2d). Based on the kinases marked in the human kinase map, we have compiled the current “list of human ‘degradable’ kinases based on PROTAC”. We believe that more and more kinases will be degraded by PROTAC technology in the future. In addition, we look forward to the joint efforts of scientists all over the world to achieve the goal of ‘each protein has a corresponding small-molecule degrader’ in the future.

In the past 2 years, the field of protein degradation has seen a rapid development and there has been many new literatures and technologies about not only PROTAC, but also molecular glue, LYTACs, AUTACs, ATTECs, RIBOTACs, PhosTACs, etc. A number of excellent reviews^8–13^ have been published to discuss these areas and progress. Due to space limitations, we will focus on the discussion about PROTAC degradation technology in this article over the period of 2020–2021. In the following pages, we will introduce the advances of PROTACs in disease fields, other PROTACs technologies, and the clinical progress of PROTACs. In different disease fields, cancers currently account for the largest part of the research on small-molecule degraders. We will generally introduce them in different sub-fields such as signaling pathways, transcriptional regulation, and cell cycle, etc. At the end of this paper, a brief summary and perspectives will be given.

## PROTACs targeting cancer-related targets

### MAPK/ERK signaling pathway-related proteins

#### AR

Androgen receptor (AR) is a member of the nuclear hormone receptor superfamily, which plays a vital role in the maintenance of male secondary sexual characteristics and the development of the prostate. Androgen receptor disorder is the main cause of prostate cancer. Metastatic castration-resistant prostate cancer(mCRPC) remains incurable and lethal. A number of AR antagonists have been developed to treat advanced prostate cancer, such as **Enzalutamide**, **Apalutamide**, and **Darolutamide.**^14^ Unfortunately, patients with these AR antagonists ultimately developed drug resistance. Most tumors are resistant to AR antagonists due to AR signaling continue to function and drive tumor growth and progression.^15^ There is an urgent need to develop new treatment strategies to treat prostate cancer, especially metastatic castration refractory prostate cancer (mCRPC).

**Fig. 4:**
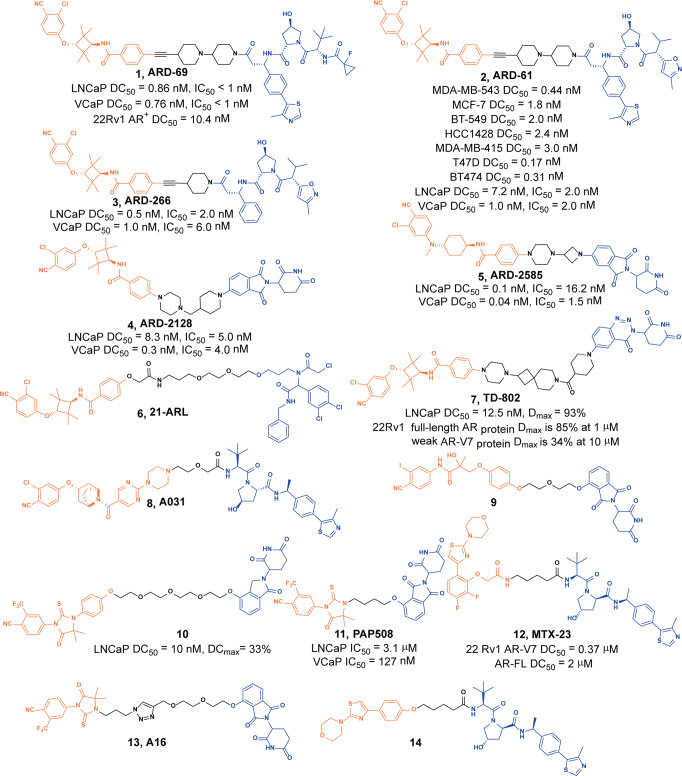
The representative PROTACs targeting AR

Wang group has been committed to the development of AR degraders. Since 2019, they have reported a number of high-efficiency AR degraders by mainly using AR antagonists as AR ligands and linking them with different E3 ligands such as VHL or CRBN to obtain different degraders. In the subsequent optimization process, they respectively modified the AR ligands, E3 ligands, linkers and achieved good results. In 2019, they first reported the degrader **1** (**ARD-69**, Fig. 4),^16^ a high-efficiency degrader obtained by connecting an AR ligand to VHL, could efficiently induce the degradation of AR protein in LNCaP cells, VCaP cells, and 22Rv1 AR^+^ cells. The DC_50_ were 0.86 nM, 0.76 nM, and 10.4 nM, respectively. It could also effectively reduce the level of AR protein in mouse xenograft tumor tissues and had a strong inhibitory activity in AR^+^ prostate cancer cell lines.

Subsequently, they optimized the VHL ligand and obtained the degraders **2** (**ARD-61**, Fig. 4)^17^ and **3** (**ARD-266**, Fig. 4)^18^ based on **ARD-69**. They found that the AR degradation activities of these two degraders were slight worse than that of **ARD-69** in LNCaP cells and VCaP cells. But they could effectively induce the degradation of AR in other AR^+^ breast cancer cell lines. Also, they were more effective than **Enzalutamide** in inhibiting cell growth and inducing cell cycle arrest and apoptosis.

In 2021, they used CRBN ligand to replace the VHL ligand and obtained the degrader **4** (**ARD-2128**, Fig. 4),^19^ which achieved 67% oral bioavailability in mice. Oral administration of **4** (**ARD-2128**) could effectively induce degradation of AR protein and effectively inhibit the growth of tumors in mice. This study demonstrated the possibility of developing an orally active AR degrader for the treatment of prostate cancer.

In order to improve its degradation activity, they further optimized and reduced the molecule weight and obtained the degrader **5** (**ARD-2585**, Fig. 4).^20^ They found that the new degrader **5** (**ARD-2585**) had a significant increase in the degradation activity of AR in LNCaP cells and VCaP cells with the DC_50_ of 0.1 nM and 0.04 nM, respectively, also it could effectively inhibit cell growth in those cell lines with the IC_50_ values of 1.5 nM and 16.2 nM, respectively. Moreover, it achieved excellent pharmacokinetics and 51% oral bioavailability in mice.

In 2021, Cravatt group discovered that DCAF11 can be used as the E3 ligases for protein degradation, and reported the ligand **21-SLF** that can efficiently bind to DCAF11.^21^ So they synthesized an AR-targeting degrader **6** (**21-ARL**, Fig. 4) based on **21-SLF** and found it could induce dose-dependent degradation of AR in normal 22Rv1 cells, but it could not cause changes in AR protein in DCAF11-KO cells. The degrader **6** (**21-ARL**) could induce the degradation of 90% AR protein at 10 μM in LNCaP cells. These data indicated that the electrophilic PROTAC (PROTAC that operate by covalent adduction of E3 ligases) combined with DCAF11 can promote the degradation of AR protein in human cells.

Subsequently, Hwang group reported a new series of AR degraders for the treatment of metastatic castration-resistant prostate cancer. Primarily, they utilized **TD-106** as an E3 ligase ligand, a novel CRBN ligand identified in their previous studies.^22^ Among all the AR degraders, the representative degrader **7** (**TD-802**, Fig. 4) could effectively induce the degradation of AR protein with DC_50_ of 12.5 nM and the D_max_ of 93% in LNCaP prostate cancer cells.^23^ In addition, the degrader **7** (**TD-802**) showed good liver microsomal stability and pharmacokinetic properties in vivo.

In order to find PROTACs with lower toxicity and better binding affinity than before, Wang group designed and synthesized several series of AR PROTACs by using CRBN/VHL E3 ligands and AR antagonists in 2021.^24^ They tested the cell inhibition of these synthetic compounds in AR-positive VCaP cells at different concentrations, and the degrader **8** (**A031**, Fig. 4) could inhibit 69.56% of the cell viability under 1.0 µM.

In 2021, another series of AR PROTACs based on **Bicalutamide** analogs and thalidomide were designed, synthesized, and biologically evaluated by the Kim group.^25^ Several novel PROTACs had their abilities to induce the degradation of AR. In particular, the degrader **9** (Fig. 4) induced the degradation of AR in a dose and time-dependent manner with DC_50_ of 5.2 µM in LNCaP cells.

In addition to the above examples, **Enzalutamide** had also been used in the design of AR degraders. The degrader **10** (Fig. 4) was reported by Skidmore group based on **Enzalutamide** in 2020.^26^ It was a potent degrader, whose DC_50_ was 10 nM and D_max_ was 33% in LNCaP cells. In addition, it showed an inhibitory effect on the proliferation of prostate tumor cells. The discovery of **Enzalutamide**-based PROTACs were expected to overcome the drug resistance that conventional drugs bring to patients.

The degrader **11** (**PAP508**, Fig. 4) was developed as a new type of PROTAC for AR protein by Lin group in 2020.^27^ The results showed that the degradation activities of **11** (**PAP508**) on AR protein depended on the action of proteasome, and the degradation effect was concentration and time dependent in LNCaP and VCaP cells. Also, it could inhibit the proliferation, migration, and invasion of prostate cancer cells.

AR-V7 is an AR variant with a truncated C-terminus. It has been confirmed that AR-V7 expression was induced by ADT and was associated with prostate cancer cell resistance. Recently, Kim group used the PROTAC technology to develop the degrader **12** (**MTX-23**, Fig. 4) to simultaneously target and induce the degradation of AR-V7 and AR full-length (AR-FL) proteins.^28^ The experimental results showed that the DC_50_ of degrader **12** (**MTX-23**) induced degradation of AR-V7 and AR-FL were 0.37 µM and 2 µM, respectively. The degrader **12** (**MTX-23**) inhibited the proliferation of prostate cancer cells, and only induced apoptosis in androgen-responsive prostate cancer cells.

Also in 2021, Ke group developed the AR degrader **13** (**A16**, Fig. 4) based on AR agonist **RU59063** and **Phthalimide.**^29^ They found these degraders could reduce the level of AR protein with the ranging from 6% to 32% at 20 µM, and the degrader **13** (**A16**) had the best activity (32% degradation at 20 µM) on reducing the level of AR protein in LNCaP cells.

In 2021, Samajdar group designed AR-V7 protein degrader **14** (Fig. 4) based on AR-DBD binder **VPC-14228.**^30^ The DC_50_ was 0.32 µM for AR-V7 protein in 22Rv1 cells and **14** was found to have modest oral bioavailability.

Finally, we compared the reported AR degraders (Table 1). It was found that there were various types of AR warheads currently in AR PROTACs design, but more of them were Pfizer AR antagonist derivatives, followed by **Enzalutamide** derivatives and other binders were less used. In terms of the selection of E3 ligases, currently CRBN and VHL were both frequently selected, and other E3 ligases were used less frequently.

**Table 1:** The summary and comparison of PROTACs targeting AR

No.	PROTAC	Warhead	E3 ligase
1	**ARD-69(1)**	Pfizer’s AR antagonist derivative	VHL
2	**ARD-61(2)**	Pfizer’s AR antagonist derivative	VHL
3	**ARD-266(3)**	Pfizer’s AR antagonist derivative	VHL
4	**ARD-2128(4)**	Pfizer’s AR antagonist derivative	CRBN
5	**ARD-2585(5)**	Pfizer’s AR antagonist derivative	CRBN
6	**21-ARL(6)**	Pfizer’s AR antagonist derivative	DCAF11
7	**TD-802(7)**	Pfizer’s AR antagonist derivative	CRBN
8	**A031(8)**	Pfizer’s AR antagonist derivative	VHL
9	**9**	Pfizer’s AR antagonist derivative	CRBN
10	**10**	**Enzalutamide** derivatives	CRBN
11	**PAP508(11)**	**Enzalutamide** derivatives	CRBN
12	**MTX-23(12)**	AR-DBD binder	VHL
13	**A16(13)**	**Enzalutamide** derivatives	CRBN
14	**14**	AR-DBD binder	VHL

#### BRAF and BRAF^*V600E*^

RAF kinase family regulates cell proliferation, growth, differentiation, and survival through the RAS–RAF–MEK–ERK signaling pathway. BRAF^*V600E*^ expression has been reported in a wide variety of human cancers.^31–33^ Small-molecule drugs targeting BRAF^*V600E*^ mutation include **Dabrafenib**, **Vemurafenib**, and **Encorafenib.**^34^ They have shown good effects in clinical application, but the generation of drug resistance limits their long-term use. These small-molecule inhibitors mainly play roles by binding to the catalytic pocket of RAF, but cannot inhibit dimerization, another key link of RAF activation, so they can not completely inhibit the activity of RAF.^35,36^ The limitations of current RAF inhibitors provide a rationale for the exploration of alternative therapeutic strategies by employing novel inhibitor mechanisms of action.

**Fig. 5:**
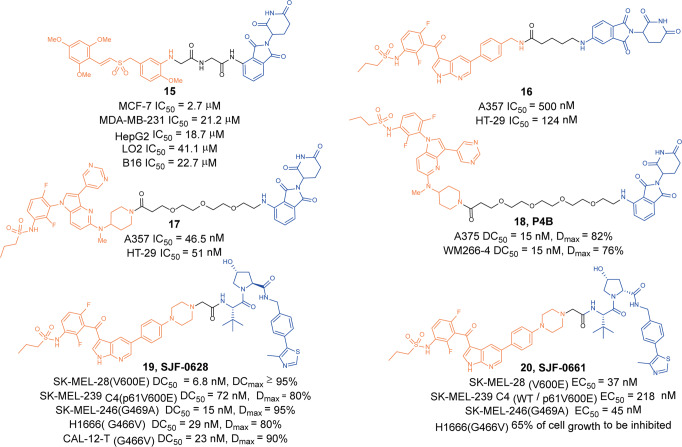
The representative PROTACs targeting BRAF and BRAF^*V600E*^

In 2019, Gou group designed a series of BRAF degraders based on **RGS** and pomalidomide.^37^ They found that degrader **15** (Fig. 5) could induce the degradation of BRAF protein in MCF-7 cells and it could effectively show antiproliferative activity on cancer cells by inducing apoptosis.

In 2020, Cullgen used **Vemurafenib** and **BI882370** as ligands for BRAF^*V600E*^ protein. By analyzing the binding mode of inhibitors and proteins, they designed a series of degraders and found that **16** (Fig. 5) based on **Vemurafenib** and **17** (Fig. 5) based on **BI882370** had better degradation activity of BRAF^*V600E*^ protein.^38^ They also found that degrader **16** could induce the degradation of BRAF^*V600E*^ protein at 12 nM, while degrader **17** had degradation effect at 37 nM. At the same time, experiments had also shown that the two degraders had no degradation activity to wild-type BRAF protein. Finally, they performed anti-proliferation experiments with **16** and **17** on A375 cells and HT-29 cells, respectively. The experimental results showed that the inhibitory effect of **Vemurafenib**-based degrader **16** on A375 cells was worse than that of inhibitor **Vemurafenib**. The IC_50_ in HT-29 cells was 124 nM. And **BI882370**-based degrader **17** had the same inhibitory effect on A375 cells and HT-29 cells with IC_50_ of 46.5 nM and 51 nM, respectively.

Similarly, in 2020, Sicheri group developed a series of novel PROTACs targeting BRAF based on different BRAF inhibitors and E3 ligands (pomalidomide and VHL).^39^ The most effective degrader **18** (**P4B**, Fig. 5) induced the selective degradation of BRAF^*V600E*^ but not the wild-type BRAF, although degrader **18** (**P4B**) had the same affinity for BRAF^*WT*^ and BRAF^*V600E*^. Downregulation of BRAF^*V600E*^ induced by degrader **18** (**P4B**) suppressed the MEK/ERK kinase cascade in melanoma cells and impaired cell growth in culture. In addition, the degrader **18** (**P4B**) displayed effectively BRAF^*V600D*^ and BRAF^*G466V*^ mutant cells. These findings highlighted a new approach to modulating the functions of oncogenic BRAF mutants and provided a framework to treat BRAF-dependent human cancers.

Recently, Crews group developed a VHL-based degrader **19** (**SJF-0628**, Fig. 5) and negative control degrader **20** (**SJF-0661**, Fig. 5) by coupling **Vemurafenib** to VHL through a rigid piperazine linker.^40^ They found the degrader **19** (**SJF-0628**) could induce the degradation of BRAF^*V600E*^ protein in a variety of cell lines but did not induce the degradation of BRAF^*WT*^ protein. In SK-MEL-28 cells, the DC_50_ to BRAF^*V600E*^ was 6.8 nM and the D_max_ exceeded 95%. It could not only induce the degradation of BRAF^*V600E*^, but also induce the degradation of a variety of BRAF mutants in a variety of cell lines, including BRAF-p61^*V600E*^, BRAF^*G469A*^, BRAF^*G466V*^*,* and so on. Subsequently, they tested the inhibitory effect of the degrader **19** (**SJF-0628**) on tumor cells. They found that in SK-MEL-28 cells (BRAF^*V600E*^) the EC_50_ of the degrader **19** (**SJF-0628**) was 37 nM. In SK-MEL-239 C4 cells(BRAF-p61^*V600E*^), the EC_50_ was 218 nM. In SK-MEL 246 cells (BRAF^*G469A*^), the EC_50_ was 45 nM.

#### eEF2K

eEF2K (eukaryotic elongation factor 2 kinase) is known as Calmodulin-dependent kinase III (CaM kinase III). eEF2K can phosphorylate its only intracellular substrate protein eEF2 and inhibit the peptide-chain extension stage during protein synthesis, and reduce the consumption of amino acids and energy so that the cell can survive under metabolic stress.^41,42^ The expression and activity of eEF2K in some tumor cells can affect the proliferation, migration, invasion, and other physiological processes^43–46^ But eEF2K shows different effects in different tumor cells. However, the eEF2K inhibitors have not achieved the expected effect in cancer treatment.

**Fig. 6:**
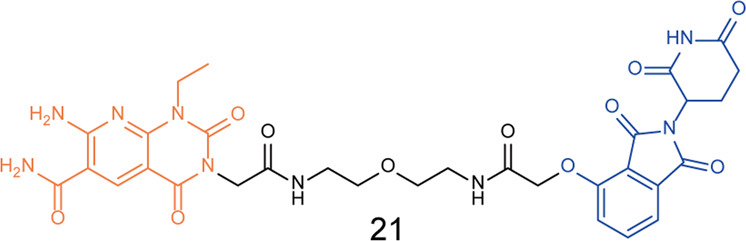
The representative PROTAC targeting eEF2K

In 2021, Ouyang group analyzed the binding mode of eEF2K inhibitor **A484954** and eEF2K protein, and obtained a series of degraders by linking it to different E3 ligase ligands.^47^ Western blotting experiments determined that the degrader **21** (Fig. 6) had the best eEF2K degradation activity in MDA-MB-231 cells, and the degradation activity Dr value was as high as 56.7%. It was also found that the degrader could induce apoptosis in MDA-MB-231 cells.

#### EGFR

EGFR, a member of the epidermal growth factor receptor (HER) family, is a glycoprotein belonging to tyrosine kinase-type receptor, which contains three regions: extracellular ligand-binding region, transmembrane region, and intracellular kinase region. As ligands, EGF and TGFα can activate EGFR to cause dimerization and guide the phosphorylation of downstream proteins, including MAPK, AKT and JNK pathways. Studies have shown that EGFR is overexpressed and abnormally expressed in many solid tumors and is associated with cell proliferation, angiogenesis, tumor invasion and tumor metastasis.^48^ Among them, non-small cell lung cancer(NSCLC), one of the most aggressive cancers, is closely related to aberrant EGFR signaling.^49^ Although three generations of small-molecule EGFR inhibitors have been approved by FDA for the treatment of NSCLC patients, drug resistance resulting from continuously heterogeneous mutations (EGFR^*C797S*^) remains a problem that inhibitors cannot overcome.^50^

**Fig. 7:**
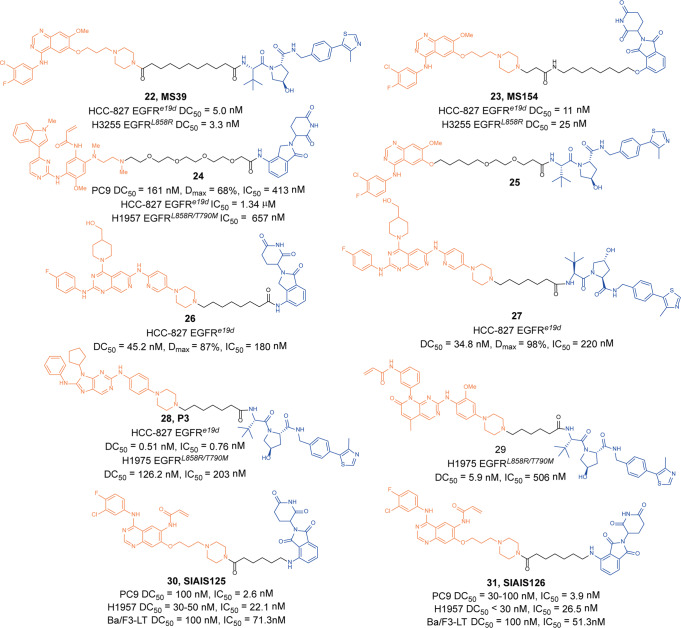
The representative PROTACs targeting EGFR

In 2019, Jin group designed a new class of EGFR PTOACs based on **Gefitinib** and different E3 ligands.^51^ The degrader **22** (**MS39**, Fig. 7) and degrader **23** (**MS154**, Fig. 7) which were based on VHL and CRBN potently induced the selective degradation of EGFR mutants but not EGFR^*WT*^ and inhibited lung cancer cells proliferation. In HCC-827 (EGFR^*e19d*^) cells and H3255 (EGFR^*L858R*^) cells, both the degrader **22** (**MS39**) and degrader **23** (**MS154**) could efficiently induce the degradation of mutant EGFR proteins, with DC_50_ of 5.0 nM, 3.3 nM, and 11 nM, 25 nM, respectively. While EGFR could not be degraded in OVCAR8(WT) cells and H1299 (WT) cells, indicating that the degrader **22** (**MS39**) and degrader **23** (**MS154**) had selective degradation activity against mutant EGFR. In addition, degrader **22** (**MS39**) had sufficient in vivo PK properties and was suitable for in vivo efficacy studies.

At the same time, a new class of EGFR PROTACs based on **Osimertinib** and lenalidomide was disclosed by Zhang group in 2020.^52^ The degrader **24** (Fig. 7) could effectively induce degradation EGFR^*Del19*^ with a DC_50_ of 161 nM and D_max_ value of 68% in PC9 cells. The degrader **24** had good antiproliferative activity against a variety of EGFR mutant cells, such as PC9(EGFR^*Del19*^) cells, HCC-827(EGFR^*Del19*^) cells, and H1957(EGFR^*L858R/T790M*^) cells, with IC_50_ of 413 nM, 1.34 µM, and 657 nM, respectively. In addition, apoptosis and G0/G1 phase arrestation of PC9 cells was significantly induced by the degrader **24** unsurprisingly.

In 2020, Zhou group also reported other EGFR degrader based on **Gefitinib** and VHL.^53^ They found that the degrader **25** (Fig. 7) could not only induce the degradation of EGFR^*L858R*^ protein, but also inhibit PD-L1 and IDO1 activities. The degrader **25** also significantly inhibited the growth of H3255 cells and enhanced the antitumor immune response of NSCLC.

In 2020, Zhang group published two works on EGFR PROTACs. In the first work, they developed EGFR degraders based on an inhibitor with pyrido[3,4-d] pyrimidine moiety, which was a fourth-generation EGFR-TKI that displayed potent inhibitory activity against EGFR^*L858R*^ and EGFR^*L858R/T790M/C797S*^. The promising degrader **26** (Fig. 7) and degrader **27** (Fig. 7) induced degradation of EGFR in HCC-827(EGFR^*e19d*^) cells with the DC_50_ values of 45.2 nM and 34.8 nM, respectively.^54^ It was found that the apoptosis and the G1 phase arrestation of HCC-827 cells were significantly induced by the two degraders. In another work, they conjugated a purine-containing derivative which was discovered as a highly potent EGFR-TKI with lenalidomide and VHL ligand to obtained a PROTAC library.^55^ The most potent degrader **28** (**P3**, Fig. 7) effectively induced the degradation of mutant EGFR with a DC_50_ of 0.51 nM, D_max_ of 80.4% and DC_50_ of 126 nM, Dmax of 90.3% in HCC-827 (EGFR^*e19d*^) cells and H1975 (EGFR^*L858R/T790M*^) cells, respectively. The degrader **28** (**P3**) also showed significant antiproliferative activity on HCC-827 (EGFR^*e19d*^) cells and H1975 (EGFR^*L858R/T790M*^) cells with IC_50_ of 0.76 nM and 203 nM. In addition, it also induced cell apoptosis and arrested cell cycle.

In 2020, Ding group also reported PROTACs targeting EGFR^*L858R/T790M*^ based on a novel selective EGFR^*L858R/T790M*^ inhibitor **XTF-262**, which was more than 100 folds selectivity over the wild-type EGFR and a panel of 465 kinases.^56^ Four E3 ligases (VHL, CRBN, MDM2, and cIAP1) were utilized for design of PROTACs. The promising degrader **29** (Fig. 7) with a VHL ligand effectively induced the degradation of EGFR^*L858R/T790M*^ with DC_50_ value of 5.9 nM and showed significant antiproliferative activity on H1975 (EGFR^*L858R/T790M*^) cells with IC_50_ of 506 nM.

In 2021, Jiang group developed two highly selective and functional EGFR-targeting PROTAC **30** (**SIAIS125**, Fig. 7) and **31** (**SIAIS126**, Fig. 7) based on **Canertinib** and CRBN ligand.^57^ Interestingly, they induced sustaining and selective degradation of EGFR^*L858R/T790M*^ in H1975 cells and EGFR^*e19d*^ in PC9 cells rather than EGFRE^*e19d/T790M*^ in PC9Brca1 cells and EGFR^*WT*^ in A549 cells, which led to the selective growth inhibition of EGFR mutant lung cancer cells instead of normal cells or A549 cells. Surprisingly, mechanistic studies showed that PROTAC-induced EGFR degradation acted through both ubiquitin–proteasome system and ubiquitin-autophagy-lysosome system. They also proved that elevated autophagy activities enhanced EGFR degradation and cell apoptosis induced by PROTACs.

Finally, we compared the reported EGFR degraders (Table 2). It was found that there were various types of EGFR warheads currently in EGFR PROTACs design, but more of them were already marketed drugs, followed by reported inhibitors. In the selection of E3 ligases, CRBN and VHL were currently used, and other E3 ligases were not used in EGFR degraders.

**Table 2:** The summary and comparison of PROTACs targeting EGFR

No.	PROTAC	Warhead	E3 ligase
1	**MS39(22)**	**Gefitinib**	VHL
2	**MS154(23)**	**Gefitinib**	CRBN
3	**24**	**Osimertinib**	CRBN
4	**25**	**Gefitinib**	VHL
5	**26**	Pyrido[3,4-d] pyrimidine moiety	CRBN
6	**27**	Pyrido[3,4-d] pyrimidine moiety	VHL
7	**P3(28)**	Purine-containing derivative	VHL
8	**29**	**XTF-262**	VHL
9	**SIAI125(30)**	**Canertinib**	CRBN
10	**SIAI126(31)**	**Canertinib**	CRBN

#### eIF4E

eIF4E (Eukaryotic translation initiation factor 4E) is a cap-binding protein that can specifically recognize the cap structure at the 5’-end of mRNA.^58^ It plays an important role in the initiation of eukaryotic translation.^59^ Studies have found that the overexpression of eIF4E is related to cancer and other diseases, and eIF4E levels are elevated in 30% of cancers, so eIF4E has become an attractive target for drug discovery.^60^

**Fig. 8:**
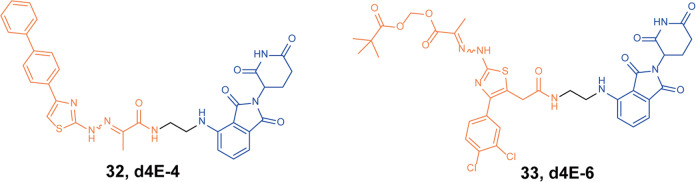
The representative PROTACs targeting eIF4E

In 2021, Arthanari group developed a new type of eIF4E inhibitor **i4EG-BiP** based on the crystal structure and proved that the inhibitor could inhibit the proliferation of cancer cells.^61^ Then they designed and synthesized a series of eIF4E degraders based on the binding mode of **i4EG-BiP** and eIF4E.^62^ Through the degradation activity test, it was found that the degrader **32** (**d4E-4**, Fig. 8) and the degrader **33** (**d4E-6**, Fig. 8) showed certain eIF4E degradation activity. When the drug concentrations were 10 µM, the obvious downregulation of eIF4E could be observed after 0.5 h of drug treatment.

#### ER

Estrogen receptors include two types: type I receptors are the classical nuclear receptors, including ERα and ERβ, which are located in the nucleus and mediate the genotype effect of estrogen. Type II receptors are membranous receptors, including membranous components of classical nuclear receptors and GPER1 (GPR30), Gaq-ER, and ER-X, which belong to the G-protein-coupled receptor family. They mediate rapid non-genotypic effects and perform indirect transcriptional regulatory functions through the second messenger system. The distribution of these two types of receptors is tissue and cell-specific and they are involved in the regulation of various functions such as reproduction, learning, memory, cognition, and so on.

It is well-known that the overexpression of estrogen receptor α(ERα) may lead to ER-positive breast cancer, which accounts for 70% of breast cancer.^63,64^ Approved endocrine therapies include aromatase inhibitors(AIs) such as **Letrozole**, selective ER modulators (SERMs) such as **Tamoxifen**, and selective ER degrader (SERDs) such as **Fulvestrant**. But the long-term use of SERMs is prone to lead to drug resistance and **Fulvestrant** is limited by poor solubility and low oral bioavailability.^65,66^ The emergence of PROTACs provides a new method for the development of drugs targeting ERα.

**Fig. 9:**
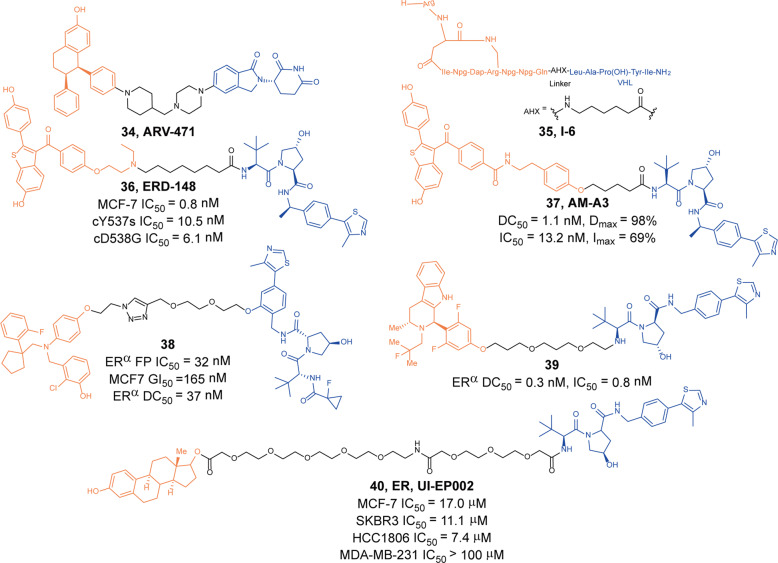
The representative PROTACs targeting ER

Since July 2021, Pfizer and Arvinas have been collaborating to advance the study of oral PROTAC **34** (**ARV-471**, Fig. 9) targeting estrogen receptors (ER).^5^ The PROTAC **34** (**ARV-471**) was the first degrader developed by Arvinas in 2018 and entered clinical studies for ER^+^/HER2^−^, locally advanced or metastatic breast cancer. In December 2020, a phase I clinical study was completed and the results showed that the PROTAC **34** (**ARV-471**) induced degradation of ER potently with encouraging clinical efficacy and tolerability. Another two trials of **34** (**ARV-471**) were scheduled to start this year. With the advancement of **34** (**ARV-471**) research, PROTACs could not only provide tools for scientific research but also serve as an important approach for drug development.

PROTACs/SNIPERs, the peptide-based target protein-degradation inducers, tend to have low cell penetrability and poor intracellular stability as drawbacks. In 2019, Qian group attempted to improve stability and cell penetration by using a lactam cyclic peptide as ERα binding ligand.^67^ The optimized degrader **35** (**I-6**, Fig. 9) induced obvious ERα degradation and inhibited MCF-7 cell growth with an IC_50_ of 9.7 µM.

In 2019, Rae group reported a new ER degrader **36** (**ERD-148**, Fig. 9).^68^ They found that in wild-type MCF-7 and ERα LBD mutant cells, the degrader **36** (**ERD-148**) showed strong inhibitory activity, the IC_50_ values in MCF-7, cY537S, and cD538G cells were 0.8 nM, 10.5 nM, and 6.1 nM, respectively. At the same time, the degrader **36** (**ERD-148**) could not only induce the degradation of wild-type ERα but also showed strong degradation activity against Y537S and D538G mutant ERα.

In 2020, Tang group developed a two-stage strategy for PROTACs screening.^69^ In stage one, the ERα ligands with a hydrazide functional group reacted with E3 ligase ligands with a terminal aldehyde group in DMSO solution to form a more than 100 compounds library. Then the ELISA screening was conducted without further purification. Among them, the degrader **A3** was screened out with DC_50_ of 10 nM and *D*_max_ of 95%. Then they transformed the degrader **A3** to a more stable degrader **37** (**AM-A3**, Fig. 30) with better degradation activity (DC_50_ = 1.1 nM, D_max_ = 98%) and cell growth inhibition (IC_50_ = 13.2 nM, *I*_max_ = 69%) in MCF-7.

In 2021, X-Chem developed a novel class of PROTACs based on a new ERα binder which was based on DNA-encoded chemical library screening.^70^ They screened 120 billion DNA-encoded molecules and found the best warhead to ERα, then they conjugated the warhead to many kinds of E3 ligase ligands by click reaction to obtain some novel PROTACs. They found the degrader **38** (Fig. 9) could induce the degradation of ERα with DC_50_ of 37 nM and inhibit MCF-7 cells growth with GI_50_ of 165 nM, and effectively inhibited ER^+^ MCF-7 tumor growth in a mouse xenograft model of breast cancer.

AstraZeneca also announced its degraders that targeted the degradation of ERα.^71^ Their study found that the affinity of degrader **39** (Fig. 9) for ERα protein was as high as 0.8 nM. In MCF-7 cells, it had a strong degradation activity for ERα protein, and the DC_50_ was 0.3 nM.

Salem group reported the degrader **40** (**UI-EP002**, Fig. 9) could effectively induce the degradation of ERα, ERβ, and GPER.^72^ **UI-EP002** would induce degradation of the plasma membrane and intracellular GPER and nuclear ERs, but could not affect others proteins lacking the estrogen targeting domain. The target specificity was further proved in human MCF-7 cells, which could effectively induce degradation of ERα, ERβ and GPER without affecting the PRs. The degrader **40** (**UI-EP002**) induced cytotoxicity and G2/M cell cycle arrest in MCF-7 breast cancer and human SKBR3 (ERα-ERβ-GPER^+^) breast cancer cells but did not induce proliferation inhibition of MDA-MB-231 breast cancer cells.

#### FGFR1/2

FGFR (fibroblast growth factor receptor) belongs to the tyrosine receptor kinase (TRK) family in the human genome, including FGFR1, FGFR2, FGFR3, and FGFR4 subtypes. The signaling pathways mediated by FGFR are required for normal cell growth and differentiation, and they are involved in physiological processes, such as neovascularization, cell proliferation, and migration, regulation of organ development, and wound healing.^73–75^ However, when FGFR is mutated or overexpressed, it will cause excessive activation of the FGFR signaling pathway and induce normal cell canceration. There are FGFR aberrations in almost all the detected malignant tumors, such as urothelial carcinoma, bile duct carcinoma, breast carcinoma, endometrial carcinoma, squamous carcinoma and so on.^76–78^ A number of companies are engaged in the research and development of FGFRs inhibitors. Although 2 FGFR inhibitors have been approved, they are all multi-target inhibitors.^79,80^ The FGFR subtype-selective inhibitors are both in the clinical development stage.^81^

**Fig. 10:**
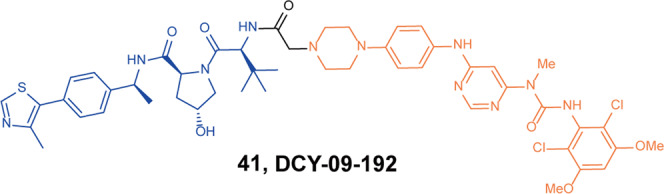
The representative PROTAC targeting FGFR1/2

In 2021, Gray group synthesized several glutarimide-based CRBN-targeting degraders with various linkers based on FGFR inhibitor **BGJ398.**^82^ Through binding affinity tests and protein-degradation experiments, it was found that the PROTACs based on **BGJ398** could degrade TEL-FGFR2 in Ba/F3 cells, while the full-length FGFR2 was poorly degraded in Kato III cells. Therefore, they used VHL ligands to replace CRBN and found the degrader **41** (**DGY-09–192**, Fig. 10) based on **BGJ398** and VHL ligands had nanomolar degradation activity for both wild-type and fusion-mutant FGFR2 proteins. Furthermore, the degrader **41** (**DGY-09-192**) showed highly selective degradation of FGFR1 and 2, as well as full-length FGFR2 or FGFR2 fusion proteins, despite retaining equivalent biochemical inhibition of all four FGFR isoform proteins. However, the degrader **41** (**DGY-09-192**) also had some limitations that need to be overcome. For example, the degrader **41** (**DGY-09-192**) showed no improvement in antiproliferative activity compared to the inhibitor **BGJ398** and did not overcome **BGJ398**-induced point mutations in the FGFR protein.

#### IGF-1R and Src

Insulin-like growth factor 1 receptor (IGF-1R) is a membrane receptor tyrosine kinase. Overexpression of IGF-1R plays a key role in the proliferation, transformation, and survival of various cancer cells, such as breast cancer, lung cancer, prostate cancer, and so on.^83^ The anti-apoptotic effect of IGF-1R can cause tumor cells to develop resistance to commonly used chemotherapy drugs or radiotherapy drugs. The protein encoded by the Src gene belongs to the Src family of kinases (SFKs), which consists of 9 members, namely Src, Lyn, Fyn, Lck, Hck, Fgr, Blk, Yrk, and Yes. Among them, Src is currently the most known member.^84^ It is a non-receptor tyrosine kinase, which is related to the survival and drug resistance of cancer cells. At present, studies have shown that the activation of Src is related to the drug resistance of IGF-1R inhibitors. Therefore, the dual inhibitory effect of IGF-1R and Src may be a feasible way to develop new antitumor drugs to overcome drug resistance.^85^

**Fig. 11:**
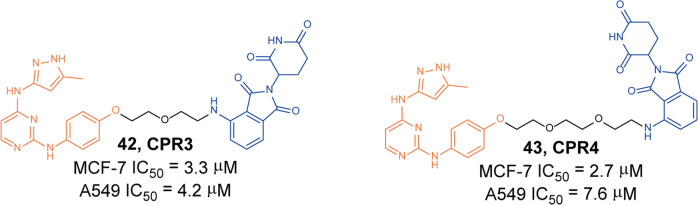
The representative PROTACs targeting IGF-1R and Src

In 2020, Lee group designed a variety of degraders based on different types of IGF-1R inhibitors and CRBN.^86^ The ligands for the targeted protein included IGF-1R/Src dual-target inhibitors. During the screening process, it was found that the degraders **42** (**CPR3**, Fig. 11) and 43 (**CPR4**, Fig. 11) based on *N*^2^-phenyl-*N*^4^-(1H-pyrazol-3-yl) pyrimidine-2,4-diamine had IGF-1R/Src dual-target degradation activity. In MCF-7 and A549 cells, the obvious degradation of IGF-1R and Src could be observed at the concentration of 5 µM. Although the degradation effect was poor, it showed obvious antiproliferative activity on MCF-7 and A549 cells, and the IC_50_ were 3.3 µM, 2.7 µM and 4.2 µM, 7.6 µM, respectively.

#### KRAS^*G12C*^

KRAS is a mouse sarcoma virus oncogene. There are three ras genes related to human cancer: HRAS, KRAS, and NRAS, which are located on chromosomes 11, 12, and 1, respectively. Among them, KRAS has the greatest impact on cancer because it serves as a molecular switch which cycles between an inactive(GDP-bound “off”) state and an active (GTP-bound “on”) state.^87,88^ It controls the pathways that regulate cell growth under normal conditions, when KRAS gene is mutated to be permanently activated, normal RAS protein can not be produced which leads to abnormal cell proliferation and cancerization. KRAS is one of the most frequently mutated oncogenes, high-frequency mutations(such as G12A, G12C, G12D, G12S, G12V, G13C, G13D) and some low-frequency mutations can activate KRAS.^89^ KRAS has mutations in a variety of cancers, among which pancreatic cancer has a mutation rate of 90%, colon cancer and lung cancer (mainly non-small cell lung cancer) have mutation rates of 30–50% and 19%, and cholangiocarcinoma accounts for about 26%. Since the KRAS protein does not have a suitable binding pocket for small inhibitors, the development of small inhibitors targeting KRAS has not made a breakthrough for a long time. However, people have been paying attention to the G12C mutation and have developed some covalent inhibitors. There have been many KRAS^*G12C*^ inhibitors were in clinical researches, but the results also showed that some patients have already developed drug resistance.^90^ PROTACs which had the advantages of overcoming drug resistance and targeting undruggable targets provide a complementary approach to cancer treatment.

**Fig. 12:**
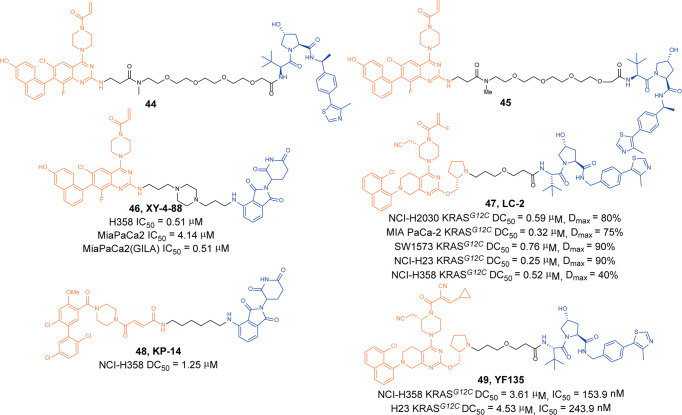
The representative PROTACs targeting KRAS^*G12C*^

In 2019, Arvinas announced its patent for using PROTAC technology to induce the degradation of KRAS^*G12C*^. They designed a large number of KRAS^*G12C*^ degraders based on **ARS-1620** derivatives and different E3 ligase ligands.^91^ They found that degraders **44** (Fig. 12) and **45** (Fig. 12) had good KRAS^*G12C*^ protein-degradation activity through degradation activity screening. In NCI-H2030 cells, the degradation of KRAS^*G12C*^ was less than 25% when the drug concentration was 300 nM, but the degradation activity was significantly enhanced as the concentration increased. The degradation activity was higher than 50% when the concentration was 1 µM. The results proved that the KRAS^*G12C*^ could also be induced degradation by PROTAC technology, which brought hope for the treatment of related diseases.

In 2020, Gray group also reported the PROTAC targeting KRAS^*G12C*^. The CRBN ligands were tethered to covalent quinazoline-based ligand **ARS-1620** at the 2-position on the quinazoline to construct a degrader library.^92^ Then KRAS^*G12C*^ degradation was screened by a fluorescent-activated cell sorting (FACS)-based assay in a high-throughput manner. But it was regrettable that these compounds only induced the degradation of an artificial GFP-KRAS^*G12C*^ fusion protein but not endogenous KARS^*G12C*^, which might be due to the inability of the lead degrader to effectively poly-ubiquitinate endogenous KRAS^*G12C*^. However, the degrader **46** (**XY-4-88**, Fig. 12) showed good antiproliferative activity in different tumor cells, such as H358 cells (IC_50_ = 0.51 µM), MiaPaCa2 cells (IC_50_ = 4.14 µM) and MiaPaCa2(GILA) cells (IC_50_ = 0.51 µM). Nevertheless, they developed a series of critical assays for in vitro activity evaluation and laid the foundation for the emergence of subsequent KARS PROTACs.

In 2020, Crews group also reported the first-in-class endogenous KRAS^*G12C*^ degraders based on covalent KRAS inhibitor **MRTX849** and VHL ligand.^93^ A variety of linkers were introduced at *N*-methyl moiety of the pyrrolidine to generate a PROTAC library. The degrader **47** (**LC-2**, Fig. 12) was identified as the most potent KRAS^*G12C*^ degrader through screening, which induced rapid and sustained degradation of endogenous KRAS^*G12C*^ with D_max_ of 80% and DC_50_ of 0.59 µM in NCI-H2030 cells. In addition, they have also tested the degradation activity of the degrader **47** (**LC-2**) in a variety of cells and found that it could efficiently induce the degradation of KRAS^*G12C*^ protein in different cells. It also modulated downstream ERK signaling in homozygous and heterozygous KRAS-mutant cell lines.

Recently, Chen group designed and synthesized KRAS^*G12C*^ degraders based on **KRas G12C-IN-3** and pomalidomide.^94^ They found the degrader **48** (**KP-14**, Fig. 12) showed the best KRAS^*G12C*^ degradation activity in NCI-H358 cells with a DC_50_ of 1.25 μM. Mechanism experiments have proved that degrader **48** (**KP-14**) selectively induced the degradation of KRAS^*G12C*^ through the protein–ubiquitin system, but could not induce the degradation of other KRAS mutants such as G13D. In addition, the degrader **48** (**KP-14**) exhibited effective antiproliferative activity and inhibited the formation of tumor colonies in NCI-H358 cells.

Also, based on the structure of **LC-2**, Lu group replaced its vinyl moiety to obtain a series of KRAS^*G12C*^ degraders.^95^ They found that degrader **49** (**YF135**, Fig. 12) had the best degradation activity in H358 and H23 cells. It could induce the degradation of KRAS^*G12C*^ protein with DC_50_ of 3.61 µM and 4.53 µM, respectively. At the same time, the degrader also showed good antiproliferative activity in these two tumor cells, and the IC_50_ were 153.9 and 243.9 nM, respectively. The degrader **49** (**YF135**) was the first reversible covalent PROTAC that was capable of recruiting VHL-mediated proteasomal to induce the degradation of KRAS^*G12C*^.

#### MEK

MAP kinase kinase or mitogen-activated protein kinase (MEK) is an important signal molecule in Ras–RAF-ME- ERK pathway. MEK1 and MEK2 are two subtypes of the MEK family. MEK1 and MEK2 activate ERK in cell proliferation, apoptosis, cell differentiation and play an important role in tumorigenesis.

**Fig. 13:**
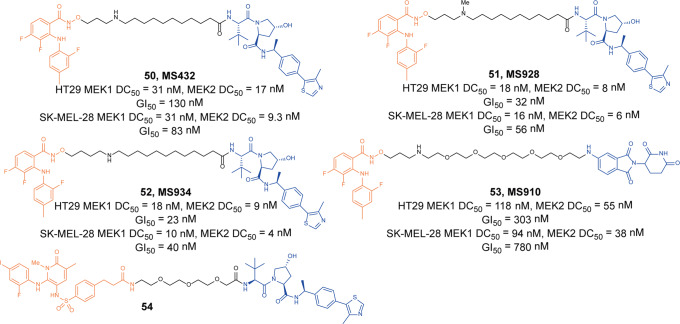
The representative PROTACs targeting MEK

Jin group published two articles on MEK degraders in 2019 and 2020. In 2019, they synthesized the MEK degraders based on the structure of non-ATP competitive MEK inhibitor **PD0325901** and VHL. They found that the degrader **50** (**MS432**, Fig. 13) showed the strongest degradation activity, but it was not selective for MEK1 and MEK2.^96^ In HT-29 cells, the degradation activity DC_50_ of MEK1 and MEK2 were 31 nM and 17 nM, and the inhibitory activity of GI_50_ was 130 nM. In SK-MEL-28 cells, the DC_50_ of MEK1 and MEK2 were 31 nM and 9.3 nM, and the inhibitory activity GI_50_ was 83 nM. It could also inhibit ERK phosphorylation in cells, and could inhibit the proliferation of colorectal cancer and melanoma cells more effectively than the negative control.

In 2020, they also developed the other MEK degraders based on **PD0325901** and different E3 ligases ligands. They obtained VHL-based degraders **51** (**MS928**, Fig. 13), **52** (**MS934**, Fig. 13), and CRBN-based degrader **53** (**MS910**, Fig. 13),^97^ and they found these degraders could effectively induce the degradation of MEK1/2 through the ubiquitin–proteasome system, but they were not selective for MEK1 and MEK2. These degraders also inhibited downstream signal transduction and cancer cell proliferation. The degradation activities and inhibitory activities of VHL ligand-based degraders **51** (**MS928**) and **52** (**MS934**) in HT-29 cells and SK-MEL-28 cells were equivalent to the degrader **50** (**MS432**), but the CRBN-based degrader **53** (**MS910**) was significantly weaker than the degrader **50** (**MS432**).

In 2020, Perry group reported the MEK1/2 degrader based on allosteric MEK inhibitor **Refametinib** derivative and VHL.^98^ They found the degrader **54** (Fig. 13) had the efficacy of inducing the degradation of MEK1/2 at 10 µM. The degrader **54** had a stronger effect on cell proliferation than inhibitor and showed a better efficacy of suppression of ERK1/2 phosphorylation and IL-6 secretion.

#### Myc

MYC is a broadly acting transcription factor that regulates cell differentiation and proliferation through multiple mechanisms.^99^ MYC gene is currently the most studied nuclear protein carcinoid gene, including C-MYC, N-MYC, L-MYC, and R-MYC. C-MYC is one of the most common activated proto-oncogenes.^100^ Cancers regulated by C-MYC account for about 20% of human cancers.^101^ However, because it is extremely difficult to develop drugs that directly target the MYC protein, MYC has become an “undruggable” target.^102^

**Fig. 14:**
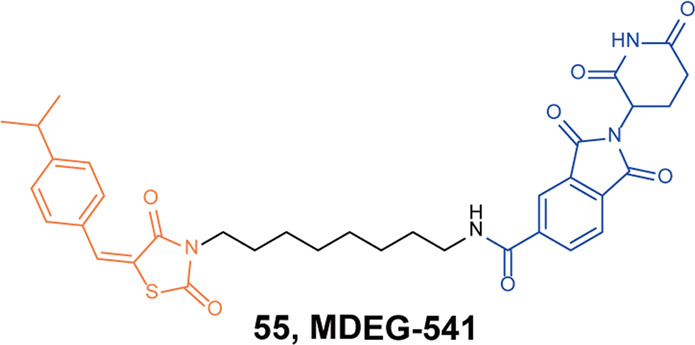
The representative PROTAC targeting Myc

Schneider group designed and synthesized a MYC degrader based on the MYC-MAX dimerization inhibitor **10058-F4** derivative **28RH** and thalidomide.^103^ They found the degrader **55** (**MDEG-541**, Fig. 14) could rapidly induce the degradation of MYC protein in HCT116 or PSN1 cells, and almost completely induced the degradation of MYC protein when the drug concentration was 20 µM. In addition, they also found that the degrader **55** (**MDEG-541**) could degrade GSPT1 and GSPT2 in a variety of cells.

#### p38

p38 mitogen-activated protein kinase (MAPK) family consists of p38α, p38β, p38γ, and p38δ.^104^ p38α is widely expressed in almost all cell types and attributed the main function in the p38 family. p38β expressed at a lower level and may have redundant functions with p38α. The function of p38α is highly dependent on cell type and environment. For example, during tumorigenesis, p38α usually plays a tumor-suppressive role in normal epithelial cells, while in malignant cells p38α tends to support tumor development.^105^ However, currently available p38α inhibitors have not shown the expected efficacy in clinical trials.

**Fig. 15:**
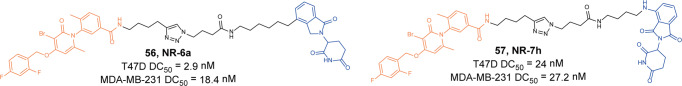
The representative PROTACs targeting p38

In 2020, Nebreda declared their design and synthesis of a series of novel p38α/β degraders based on an ATP competitive inhibitor of p38α/β **PH-797804** and thalidomide analogs.^106^ The degraders **56** (**NR-6a**, Fig. 15) and **57** (**NR-7h**, Fig. 15) were the two representative degraders which could efficiently induce the degradation of p38α/β without other related kinases at nanomolar concentrations in several mammalian cell lines. The degraders **56** (**NR-6a**) and **57** (**NR-7h**) inhibited the p38α signaling pathway induced by stress and cytokines, which provided a useful tool to investigate function and influence of the p38 MAPK pathway in diseases.

#### PDEδ

KRAS is recognized as one of the targets of cancer treatment. However, no small-molecule drug targeting KRAS has been approved. PDE δ is a shuttling factor of RAS which can prevent KRAS binding to the endomembrane and promote its diffusion in the whole cell.^107^ Subsequently, KRAS is released from PDEδ by releasing factor Arl2 and transported to the plasma membrane. Several KRAS-PDEδ inhibitors with potent affinity have been reported, such as **Deltarasin**, **Deltazinone**, and **Deltasonamide.**^108^ However, Arl2 induces the fast release of high-affinity inhibitors from PDEδ and finally reduces their antitumor efficacy.^109^

**Fig. 16:**
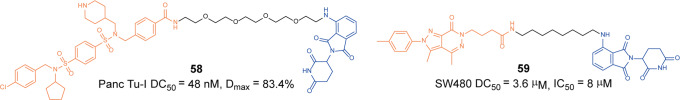
The representative PROTACs targeting PDEδ

In 2019, Waldmann group reported the development of degraders based on PDEδ inhibitor **Deltasonamide** and thalidomide.^110^ The degrader **58** (Fig. 16) could efficiently and selectively induce the degradation of PDEδ with DC_50_ of 48 nM and *D*_max_ of 83.4% in Panc Tu-I cells. The application of the PDEδ degrader **58** increased sterol regulatory element binding protein (SREBP)-mediated gene expression of enzymes involved in lipid metabolism, resulting in elevated levels of cholesterol precursors. It demonstrated that PDEδ function played a role in the enzymatic regulation of the mevalonate pathway.

In 2020, Sheng group developed a series of potent PDEδ degraders by connecting PDEδ inhibitor **Deltazinone** and pomalidomide.^111^ The most promising degrader **59** (Fig. 16) efficiently induced the degradation of PDEδ with the DC_50_ value of 3.6 μM, and exhibited significantly improved antiproliferative potency in KRAS-mutant SW480 cells. In addition, the degrader **59** also achieved significant tumor growth inhibition in the SW480 xenograft model. This approach offered an effective lead degrader for the treatment of KRAS-mutant cancer.

#### SHP2

Src homology 2 domain-containing phosphatase 2 (SHP2) belongs to protein tyrosine phosphatase family.^112^ SHP2 mutations exist in most tumor cells. Moreover, this target has been confirmed to be related to a variety of signaling pathways. For example, in the RAS-ERK pathway, SHP2 acts as its upstream positive regulator, which promotes cancer cell proliferation by phosphorylating ERK. Therefore, the inhibition of SHP2 can inhibit the growth of cancer cells and induce apoptosis.^113^

**Fig. 17:**
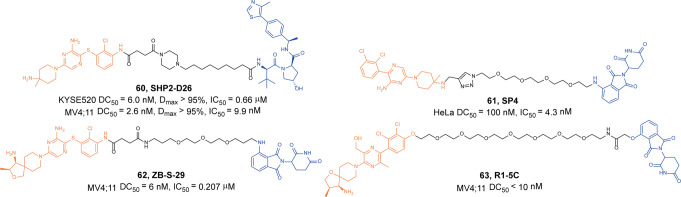
The representative PROTACs targeting SHP2

In 2020, Wang group developed a SHP2 degrader **60** (**SHP2-D26**, Fig. 17) conjugated with the SHP2 inhibitor **SHP099** and VHL ligand.^114^ The degrader **60** (**SHP2-D26**) could induce the rapid and efficient degradation of SHP2 protein in KYSE520 cells (DC_50_ = 6.0 nM) and MV4; 11 cells (DC_50_ = 2.6 nM), and was capable of reducing SHP2 protein levels by more than 95% in cancer cells. Compared to inhibitor **SHP099**, the degrader **60** (**SHP2-D26**) exhibited more than 30-folds of potent inhibition to cell growth in KYSE520 and MV4;11 cancer cell lines.

In 2020, Li group developed a novel SHP2 degrader **61** (**SP4**, Fig. 61) also conjugated with the SHP2 inhibitor **SHP099** and CRBN ligand through a PEG linker.^115^ The degrader **61** (**SP4**) successfully induced the moderate degradation of SHP2 in HeLa cells with the *D*_max_ about 40% at 500 nM after 24 h of treatment. At the same years, Zhou group developed another SHP2 degrader **62** (**ZB-S-29**, Fig. 17) conjugated with the potent and selective SHP2 inhibitor **TNO155** and CRBN ligand.^116^ The degrader **62** (**ZB-S-29**) effectively induced the degradation of SHP2 protein in a time and dose-dependent manner with a DC_50_ of 6.02 nM in MV4;11 cells, and induced more than 90% SHP2 degradation at 500 nM after 24 h of treatment. Moreover, it exhibited significant cell proliferation inhibition in MV4;11 cells and induced apparent G1 phase arrest or apoptosis in a dose-dependent manner. Subsequently, another SHP2 degrader **63** (**R1-5C**, Fig. 17) developed by conjugating **RMC-4550** with pomalidomide was reported.^117^ The degrader **63** (**R1-5C**) exhibited highly selective SHP2 degradation with low concentration, suppressed MAPK signaling, and inhibited cancer cell growth.

### PI3K/Akt signaling pathway-related proteins

#### AKT

The serine/threonine kinase AKT is a central component of the phosphoinositide 3-kinase (PI3K) signaling cascade and a key regulator of critical cellular processes, including proliferation, survival, and metabolism. Hyperactivation of AKT, due to gain-of-function mutations or amplification of oncogenes (receptor tyrosine kinases and PI3K) or inactivation of tumor suppressor genes (PTEN, INPP4B, and PHLPP), is one of the most common molecular perturbations in cancer and promotes malignant phenotypes associated with tumor initiation and progression.^118^ Thus, AKT is an attractive therapeutic target.

**Fig. 18:**
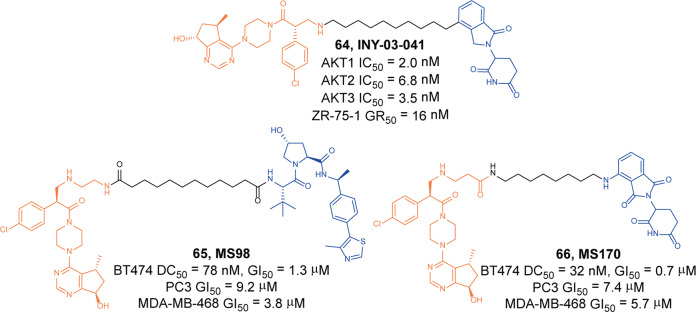
The representative PROTACs targeting AKT

In 2020, Toker group described a degrader **64** (**INY-03-041**, Fig. 18) by conjugating lenalidomide and the most advanced AKT inhibitor **GDC-0068**.^119^ The degrader **64** (**INY-03-041**) inhibited AKT1, AKT2, and AKT3 with the IC_50_ values of 2.0 nM, 6.8 nM, and 3.5 nM, respectively, while the IC_50_ values of **GCD-0068** were 5.0 nM, 18.0 nM, and 8.0 nM, respectively. They found degrader **64** (**INY-03-041**) could induce the degradation of AKT1/2/3 in a dose-dependent manner and the maximal degradation activity was observed at 100 nM to 250 nM. Also they found degrader **64** (**INY-03-041**) had good antiproliferative activities in different cell lines, such as ZR-75-1 cells (GR_50_ = 16 nM). Besides, degrader **64** (**INY-03-041**) destabilized all the three AKT isoforms and reduced the downstream signaling effects even after degrader **64** (**INY-03-041**) was washed out. It suppressed cell proliferation more potently than **GCD-0068**, which indicated that it had potential therapeutic value for targeted degradation of AKT.

In 2021, Jin group also designed the AKT degraders based on the structure of **GDC-0068**, which they coupled with different VHL and pomalidomide ligands to obtain VHL-based AKT degrader **65** (**MS98**, Fig. 18) and CRBN-based degrader **66** (**MS170**, Fig. 18).^120^ They found **65** (**MS98**) and **66** (**MS170**) showed AKT-degradation activities on BT474 cells with DC_50_ of 78 nM and 32 nM, respectively. They tested the inhibitory activity of the two degraders on different tumor cell lines, including BT474 cells, PC-3 cells and MDA-MB-468 cells. The results showed **65** (**MS98**) and **66** (**MS170**) both had inhibitory activity on these cell lines with GI_50_ were at the micromolar level. However, the degradation activities and growth inhibitory activities were both weaker than **64** (**INY-03-041**).

#### ALK

Anaplastic lymphoma kinase (ALK) is a tyrosine kinase of the insulin receptor (IR) kinase subfamily. Fusion proteins of anaplastic lymphoma kinase (ALK) are emerging therapeutic targets for cancer and other human diseases, especially non-small cell lung cancer (NSCLC) and anaplastic large cell lymphoma(ALCL).^121,122^ So far, five ALK inhibitors, including **Alectinib**, **Brigatinib**, **Ceritinib**, **Crizotinib**, and **Lorlatinib** have been approved by the FDA for the treatment of ALK-positive NSCLC. Despite the initial response to these inhibitors, drug resistance was observed within 1–2 years in most patients partly due to acquired ALK-resistant mutations.^123,124^ Hence, novel therapeutic strategies are in demand to overcome drug resistance.

**Fig. 19:**
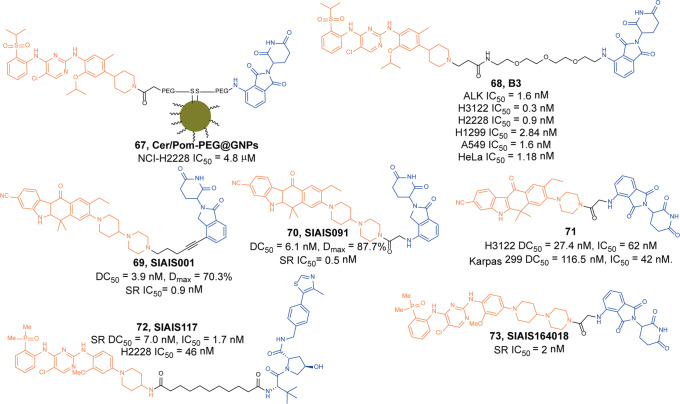
The representative PROTACs targeting ALK

In 2019, the first multi-headed(several interconnected ligands for POI and E3 ubiquitin ligase) PROTAC was developed as a gold nanoparticle (GNP)-based drug delivery system for delivering PROTAC to target ALK.^125^ The degrader **67** (**Cer/Pom-PEG@GNPs**, Fig. 19) loaded with both **Ceritinib** and pomalidomide, and showed good stability in several media. **67** (**Cer/Pom-PEG@GNPs**) potently decreased the levels of ALK fusion proteins in a dose and time-dependent manner. And it specifically inhibited the proliferation of NCI-H2228 cells with IC_50_ of 4.8 µM. In comparison with small-molecule PROTACs, the new multi-headed PROTAC promoted the formation of coacervates of POIs/multi-headed PROTAC/E3 ubiquitin ligases, and POI and E3 ubiquitin ligase interacted through multidirectional ligands and a flexible linker, thereby avoiding the need for complicated structure optimization of PROTACs.

In 2021, Li group also reported a new series of ALK degraders based on **Ceritinib** and pomalidomide.^126^ The degrader **68** (**B3**, Fig. 19) showed potent selective inhibitory activity to ALK (IC_50_ = 1.6 nM) and could decrease the level of ALK fusion protein in H3122 cells. Meanwhile, **68** (**B3**) showed better anticancer activity in vitro compared with **Ceritinib** in different cell lines and the antiproliferative activity to xenograft tumor model was acceptable. All the results demonstrated that the anticancer activities of **68 (B3)** in vitro and in vivo were valuable for further investigation.

Apart from **Ceritinib**, **Alectinib** was also widely used in the design of ALK degraders. In 2020 and 2021, Jiang group reported several ALK degraders based on **Alectinib** and lenalidomide, such as **69** (**SIAIS001**, Fig. 19) and **70** (**SIAIS091**, Fig. 19).^127^ And they found these degraders had good ALK degradation activity in SR cells as the DC_50_ were 3.9 nM and 6.1 nM, and *D*_max_ were 70.3% and 87.7%, respectively. At the same time, they also showed good anti-proliferation activity in SR cells, and the IC_50_ were 0.9 nM and 0.5 nM.

Subsequently, Xu group reported an ALK degrader **71** (Fig. 19) also based on **Alectinib.**^128^ The difference from Jiang group was that the E3 ligase ligand they used was pomalidomide, while Jiang group was lenalidomide. They found degrader **71** had the best ALK degradation activity, highest ALK binding affinity and best antiproliferative activity in such ALK-dependent cell lines as H3122 cells and Karpas 299 cells, whose DC_50_ were 27.4 nM, 116.5 nM and IC_50_ were 62 nM, 42 nM. The degrader **71** also had no obvious cytotoxicity in ALK fusion-negative cells. More importantly, it showed obvious antitumor proliferation activity in the xenograft mouse model.

Also in 2020, Jiang group reported the ALK degrader **72** (**SIAIS117**, Fig. 19),^129^ which was based on **Brigatinib** and VHL. It could not only induce the degradation of ALK protein, but also showed obvious antiproliferative activity on SR cells and H2228 cells, and the inhibitory activity was significantly better than that of the inhibitor **Brigatinib**. In addition, the degrader **72** (**SIAIS117**) could also induce the degradation of ALK^*G1202R*^ mutant protein in vitro so it had potential anti-proliferation activity of small cell lung cancer. Then they replaced the VHL ligand with pomalidomide on the basis of **72** (**SIAIS117**) and obtained the degrader **73** (**SIAIS164018**, Fig. 19) with good degradation activity for ALK.^130^ It could not only induce the degradation of the wild-type ALK protein and ALK^*G1202R*^ mutant protein, and even the EGFR^*L858R+T790M*^ mutant protein. The degrader **73** (**SIAIS164018**) also had a strong inhibitory effect on the migration and invasion of a variety of tumor cells. Also, the kinase inhibition of **73** (**SIAIS164018**) was different from that of **Brigatinib** and it rearranged the kinase inhibition of **Brigatinib**.

#### BCL-XL

In the BCL-2 family, overexpression of anti-apoptotic proteins including BCL-2, Bcl-xl, and myeloid cell 1 is a key sign of cancer partly evading apoptosis. B-cell lymphoma extra large (Bcl-xl) is a well-validated cancer target.^131^ Inhibition of these BCL-2 family proteins with small molecules has been widely studied as a cancer treatment strategy, resulting in the discovery of **ABT-263** (BCL-2 and Bcl-xl dual inhibitor) and several selective Bcl-xl inhibitors as promising anticancer drug candidates.^132^ Although these inhibitors are useful for the treatment of certain hematological malignancies, such as CLL and AML, the on-target and dose-limiting thrombocytopenia induced by Bcl-xl inhibition has limited the clinical use of these inhibitors.^133^ Bcl-xl is mainly overexpressed in many solid tumor cells and leukemia cells, and its expression is highly correlated with resistance to cancer therapy. As one of the most important validated cancer targets without a safe and effective therapeutic, Bcl-xl needs more selective methods to inhibit its activity. E3 ligases are differentially expressed in tumor cells compared with normal tissues. Thus, a method that relies on PROTAC to induce protein degradation seems to perfectly overcome this problem.

**Fig. 20:**
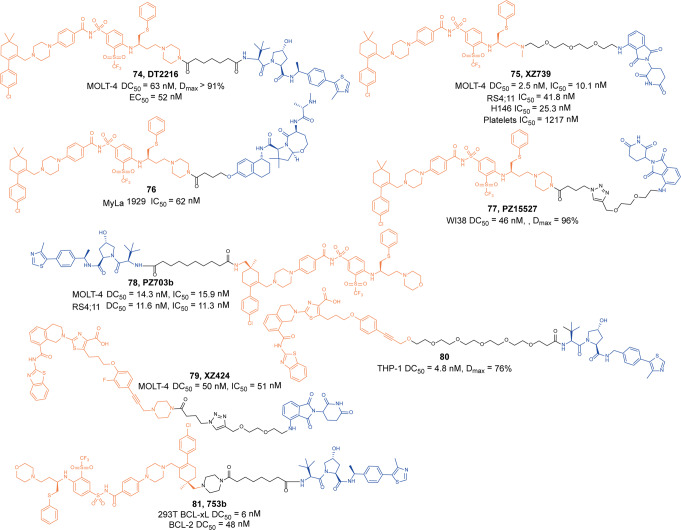
The representative PROTACs targeting Bcl-xl

Zhou and Zheng group has been devoted to the development of high-efficiency Bcl-xl degraders. They used different Bcl-xl inhibitors and E3 ligands to design and synthesize a large number of different Bcl-xl degraders, and obtained some Bcl-xl degraders with good degradation activity. In 2019, Zhou and Zheng group reported the first selective Bcl-xl degrader **74** (**DT2216**, Fig. 20) by coupling the toxicity **ABT-263** with the VHL ligand to achieve efficient Bcl-xl degradation.^134^ The DC_50_ and D_max_ were 63 nM and 90.8% in MOLT-4 T-cell acute lymphoblastic leukemia(T-ALL) cells. Since VHL was poorly expressed in platelets, degrader **74** (**DT2216**) was more potent against various Bcl-xl-dependent leukemia and cancer cells but considerably less toxic to platelets than **ABT-263** in vitro. In vivo, degrader **74** (**DT2216**), as a single drug or in combination with other chemotherapeutic drugs, can effectively inhibit the growth of several xenograft tumors without causing significant thrombocytopenia. These findings suggested that degrader **74** (**DT2216**) had greater clinical potential than **ABT-263** or other Bcl-xl inhibitors.

In addition to VHL, CRBN was also modestly expressed in platelets. Therefore, they replaced the VHL ligand with CRBN on the basis of **DT2216** to obtain a series of degraders.^135^ In the degradation activity test, they found that the degrader **75** (**XZ739**, Fig. 20), a pomalidomide-dependent degrader to Bcl-xl, had the best degradation activity. It had a DC_50_ of 2.5 nM for the degradation activity of Bcl-xl in MOLT-4 T-ALL cells and had good antiproliferative activity against a variety of cells. In MOLT-4 T-ALL cells, its antiproliferative activity was 22-folds and the selectivity to human platelets was up to 120-folds than that of inhibitor **ABT-263**. Both the antiproliferative activity and the selectivity to platelets were significantly better than inhibitor **ABT-263**, thus showing great potential for application.

Furthermore, the group also introduced IAP ligands into the degraders in 2020.^136^ They found that the degrader **76** (Fig. 20) showed the best antiproliferative activity in MyLa 1929 cells with IC_50_ of 62 nM and it could efficiently induce the degradation of Bcl-xl protein in different cell lines, such as MyLa 1929 cells, A549 cells, MDA-MB-231 cells, SW620 cells, MeWo cells, SK-MEL-28 cells, and CHL-1 cells, which proved that it was feasible to replace CRBN and VHL with IAP ligands in the design of Bcl-xl degraders.

Subsequently, they still used **ABT-263** and CRBN as ligands to design and synthesize the degrader **77** (**PZ15527**, Fig. 20).^137^ They found that the degrader could effectively induce the degradation of Bcl-xl protein in WI38 non-senescent cells (NCs) when the DC_50_ and *D*_max_ were 46 nM and 96.2%, respectively. More importantly, it could effectively clear senescent cells and rejuvenate tissue stem and progenitor cells in naturally aged mice without causing severe thrombocytopenia. With further improvements, Bcl-xl PROTACs have the potential to become a safer and more effective treatment than Bcl-xl inhibitors.

In 2021, on the basis of analyzing the binding mode of **DT2216** and Bcl-xl protein, they connected the VHL ligand with **ABT-263** from the methyl group of dimethylcyclohexene in **ABT-263**^138^ and finally found that the chiral compound **78** (**PZ703b**, Fig. 20) had the best degradation activity and cell growth inhibitory activity. In MOLT-4 and RS4;11 cells, the DC_50_ to Bcl-xl were 14.3 nM and 11.6 nM, and the IC_50_ were 15.9 nM and 11.3 nM, respectively. Its inhibitory activities were significantly better than the inhibitor **ABT-263** and the degrader **DT2216**. It could not only induce the degradation of Bcl-xl, but also inhibit but not degrade BCL-2, which showed an unprecedented mixed dual-targeting mechanism in the PROTAC. They further found that **PZ703b** can form a stable {BCL-2:PROTAC:VCB} ternary complex in living cells, which may help **PZ703b** enhance its inhibitory effect on BCL-2.

In the same year, they also developed a pomalidomide-based degrader **79** (**XZ424**, Fig. 20)^139^ for Bcl-xl degradation by conjugating a potent and selective Bcl-xl inhibitor **A-1155463**. The degrader **79** (**XZ424**) selectively induced Bcl-xl protein degradation in a dose and time-dependent manner in MOLT-4 cells but not in platelets. The DC_50_ was 50 nM and the IC_50_ was 51 nM. Subsequently, they confirmed that the platelet-toxic Bcl-xl/2 dual inhibitor **ABT-263** can be converted into platelet-sparing pomalidomide-based Bcl-xl specific PROTAC without reduction in activity or selectivity.

In addition to Zhou and Zheng group, Benowitz group also reported a degrader **80** (Fig. 20) based on **A-1155463** and VHL ligand to target the degradation of Bcl-xl protein in 2020.^140^ They found that the degrader **80** can be used in THP-1 to induce the degradation of Bcl-xl protein efficiently. And the 1.9 Å heterotetramer structure composed of (ElonginB:ElonginC:VHL):PROTAC:Bcl-xl revealed the interaction between E3 ligase and target protein and PROTAC. The mode of action between the homologous part and the partner protein provided ideas for the subsequent design of protein–protein interaction inhibitors and degraders.

Then, Zhou and Zheng group obtained a series of degraders based on **DT2216** and the binding mode of **ABT-263** in Bcl-xl, which was linked the VHL to the methyl group of dimethylcyclohexene in **ABT-263.**^141^ They found **81** (**753b**, Fig. 20) was a better dual-target degrader targeting Bcl-xl and BCL-2, it could not only induce the degradation of Bcl-xl(DC_50_ = 6 nM), but also degrade BCL-2 (DC_50_ = 48 nM) in 293T cells. It was the first dual-targeted degrader for Bcl-xl/BCL-2. In addition, they also found **81** (**753b**) exhibited more potent antitumor activity than **DT2216** in Kasumi-1 cells, which was also superior to that of **ABT-263**.

Through the comparative analysis of the degradation activity and inhibitory activity of these degraders, it was found that the degradation activities of the degraders were better, the inhibitory activities were better in most cells. But in some special cell lines, such as 293T and THP-1 cells, it was also reported that the degradation activities had no obvious influences with the inhibitory activities. The main reason may be that some cells were not sensitive to the changes of Bcl-xl protein.

Finally, we compared the reported Bcl-xl degraders (Table 3). It was found that there were two types of Bcl-xl warheads currently in Bcl-xl PROTACs design, **ABT-263** and **A-1155463**. In the selection of E3 ligases, CRBN and VHL were currently used, and other E3 ligases were not used in Bcl-xl degrades. Therefore, more Bcl-xl degraders based on different Bcl-xl inhibitors need to be developed.

**Table 3:** The summary and comparison of PROTACs targeting Bcl-xl

No.	PROTAC	Warhead	E3 ligase
1	**DT2216(74)**	**ABT-263**	VHL
2	**XZ739(75)**	**ABT-263**	CRBN
3	**76**	**ABT-263**	cIAP1
4	**PZ15527(77)**	**ABT-263**	CRBN
5	**PZ703(78)**	**ABT-263**	VHL
6	**XZ424(79)**	**A-1155463**	CRBN
7	**80**	**A-1155463**	VHL
8	**753b(81)**	**ABT-263**	VHL

#### BCR-ABL

Chronic myelogenous leukemia(CML) is most often caused by the lack of autoinhibition of the *c*-ABL kinase domain in the oncogenic fusion protein BCR-ABL.^142^ When the ABL gene is translocated from chromosome 9 to the BCR gene on chromosome 22, BCR-ABL is generated. BCR-ABL activates downstream signaling pathways to cause CML cell proliferation disorder in patients.^143^ At present, three generations of BCR-ABL inhibitors have been approved for the clinical treatment of CML. As the first-generation ABL inhibitor, **Imatinib** becomes the paradigm for targeted cancer therapy. But the intolerance and drug resistance of **Imatinib**, especially for T315I, limits its clinical application. The second-generation (**Nilotinib**, **Dasatinib**, and **Bosutinib**) and third-generation (**Ponatinib**) ABL inhibitors provide multiple options for resistance patients. According to the difference between the inhibitors and the protein binding sites, ABL inhibitors also can be divided into five categories (type I–V), among which type I (such as **Dasatinib**), type II (**Imatinib** and **Ponatinib**), and type IV (**Asciminib**) have been paied more attention. However, these inhibitors could not inhibit all resistant mutants; severe side effects also limit their clinical application.^144,145^ Therefore, it seems that the degradation of BCR-ABL may overcome these problems.

**Fig. 21:**
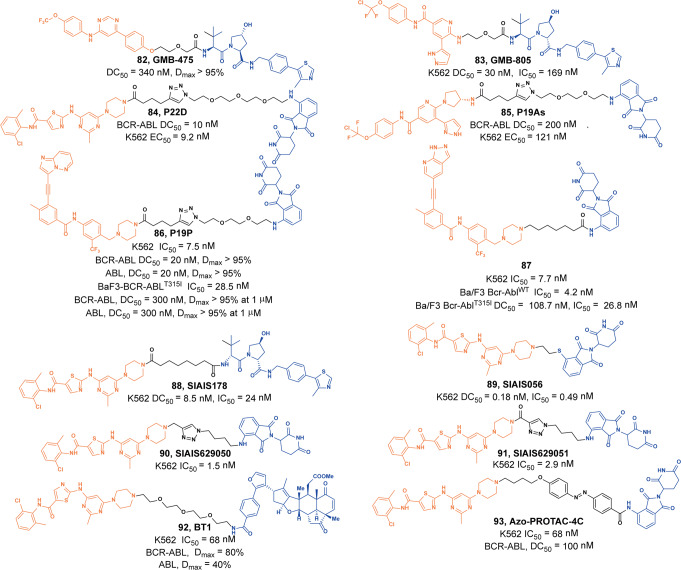
The representative PROTACs targeting BCR-ABL

In 2016, Crews group reported the first BCR-ABL PROTAC based on **Dasatinib**, but it only achieved the degradation of BCR-ABL at micromoles (>60% at 1 µM), but could not overcome the common drug-resistant mutants, especially for T315I mutant.^146^ In 2019, Crews group designed and synthesized a series of VHL-based BCR-ABL degraders by using the allosteric GNF family compounds of BCR-ABL.^147^ They optimized the linker portion of the degraders to improve both potency and cell permeability, obtaining the lead degrader **82** (**GMB-475**, Fig. 21). The degrader induced rapid proteasomal degradation and inhibition of downstream biomarkers in both human CML K562 cells and murine Ba/F3 cells. Besides, it inhibited the proliferation of BCL-ABL mutant Ba/F3 cells (T315I and G250E) more effectively than inhibitor **Imatinib**. Scaffold hopping is one of the common methods for structural modification of drugs in medicinal chemistry, which is a method to obtain novel core scaffold by changing the core structure of known active compounds. Subsequently, they employed a scaffold hopping approach to enhance the activity of **GMB-475** to obtain the degrader **83** (**GMB-805**, Fig. 21).^148^ The new BCL-ABL degrader **83** (**GMB-805**) demonstrated more than ten-folds increase in ability to induce BCL-ABL degradation, and improved pharmacokinetic properties and in vivo activity.

In 2021, Rao group developed some BCR-ABL degraders targeting all the three binding sites of BCR-ABL.^149^ These BCL-ABL degraders were designed and synthesized using four BCR-ABL inhibitors **Imatinib**, **Dasatinib**, **Asciminib**, and **Ponatinib** as target molecules. In this toolbox for inducing degradation of BCR-ABL from different binding pockets, the degraders designed on the other three BCR-ABL inhibitors all worked obviously well except the **Imatinib**-based degraders. Among them, three representative degraders, **84** (**P22D**, based on **Dasatinib**, DC_50_ = 10 nM, Fig. 10), **85** (**P19As**, based on **Asciminib**, DC_50_ = 200 nM, Fig. 10), and **86** (**P19P**, based on **Ponatinib**, DC_50_ = 20 nM, Fig. 10) exhibited effective degradation activity for wild-type BCL-ABL, and also showed good cytostatic activity. More importantly, **86** (**P19P)** could efficiently induce the degradation of wild-type and mutated BCR-ABL (**Dasatinib**-resistant T315I, **Asciminib**-resistant V468F mutants, E255K and H396R) in transfected HeLa cells without causing serious side effects. And it also exhibited better antiproliferative activity against T315I mutant BaF3 cell line, with EC_50_ of 28.5 nM. Through the above studies, it could found that PROTACs designed based on the type I inhibitor **Dasatinib**, the type II inhibitor **Bonatinib** and the type IV inhibitor **Asciminib** can successfully induce the degradation of the BCR-ABL protein. The degradation activities of PROTACs designed based on **Dasatinib** were significantly better than that of other PROTACs, while the PROTACs designed based on type II inhibitor **Imatinib** could not induce the degradation of BCR-ABL protein under the concentration of 30 µM. It indicated that the degraders based on type II inhibitors can be designed to induce the degradation of BCR-ABL protein, but it requires more careful consideration that the design of degraders based on **Imatinib** to induce the degradation of BCR-ABL. In summary, these PROTACs showed better selectivity and fewer adverse reactions than inhibitors, which indicated that PROTACs have great potential in overcoming the clinical resistance and safety issues of BCR-ABL.

Recently, Lu group described a new class of selective BCR-ABL^*T315I*^ degraders based on a BCR-ABL^*T315I*^ inhibitor **GZD824.**^150^ The degrader **87** (Fig. 21) exhibited the most potent degradation efficacy with DR of 69.89% and 94.23% at 100 and 300 nM, respectively. In addition, the degrader **87** had an IC_50_ value of 26.8 nM against Ba/F3^*T315I*^ cells and also showed significant tumor regression in this mutation xenograft model in vivo.

In 2019, Jiang group reported the degrader **88** (**SIAIS178**, Fig. 21) by connecting **Dasatinib** and VHL by extensive optimization of linkers.^151^ The degrader **88** (**SIAIS178**) induced the effective degradation of wild-type BCR-ABL with the DC_50_ value of 8.1 nM in K562 cells, and several clinically relevant resistance-conferring mutations. Moreover, it achieved significant growth inhibition of the BCR-ABL^+^ leukemic cells in vitro, and induced substantial tumor regression in vivo against K562 xenograft tumors. Subsequently, Jiang group synthesized a series of CRBN-based degraders by conjugating **Dasatinib** to pomalidomide or lenalidomide.^152^ As an important example, a pomalidomide-based degrader **89** (**SIAIS056**, Fig. 21), possessing sulfur-substituted carbon chain linker, exhibited the potent degradation of wild-type and clinically relevant resistance-conferring mutations of BCR-ABL. Furthermore, the degrader **89** (**SIAIS056**), with favorable pharmacokinetics, induced significant tumor regression against K562 xenograft tumors in vivo. In addition, they reported a highly efficient protocol to construct a new IMiD-based azide library through click reaction. The degraders **90** (**SIAIS629050**, Fig. 21) and **91** (**SIAIS629051**, Fig. 21) showed good antiproliferative activity with IC_50_ of 1.5 nM and 2.9 nM in K562 cells, and exhibited potent degradation activity of wild-type BCR-ABL in a dose-dependent manner. This approach provided help for the rapid construction of degraders libraries.

Some examples reported recently have further enriched the tools for PROTAC-induced degradation of BCR-ABL. Based on the natural product **Nimbolide**, a covalent recruiter for the E3 ligase RNF114, Nomura group developed a novel PROTAC linking **Nimbolide** to **Dasatinib** to obtain the degrader **92** (**BT1**, Fig. 21), which could selectively induce the degradation of BCR-ABL over c-ABL in leukemia cancer cells.^153^ Compared with the previously reported cereblon or VHL-recruiting BCR-ABL degraders, it showed the unique degradation specificity profiles. These achievements pave a way to develop more drug-like PROTACs for degrading BCR-ABL in the future. In 2020, Jiang group also developed a novel photo-switchable azobenzene-PROTAC **93** (**Azo-PROTAC-4C**, Fig. 21).^154^ They could control the degradation of ABL and BCR-ABL proteins in live cells by changing the configuration of Azo-PROTAC with UV-C light.

Finally, we compared the reported BCR-ABL degraders (Table 4). It was found that there were various types of BCR-ABL warheads currently in BCR-ABL PROTACs design, but **Dasatinib** was still the most frequently used inhibitor. In the selection of E3 ligases, CRBN and VHL were currently used, and other E3 ligase such as RNF114 were also used in BCR-ABL degraders.

**Table 4:** The summary and comparison of PROTACs targeting BCR-ABL

No.	PROTAC	Warhead	E3 ligase
1	**GMB-475(82)**	Allosteric GNF family compounds	VHL
2	**GMB-805(83)**	Allosteric GNF family compounds	VHL
3	**P22P(84)**	**Dasatinib**	CRBN
4	**P19As(85)**	**Asciminib**	CRBN
5	**P19P(86)**	**Ponatinib**	CRBN
6	**87**	**GZD824**	CRBN
7	**SIAIS178(88)**	**Dasatinib**	VHL
8	**SIAIS056(89)**	**Dasatinib**	CRBN
9	**SIAIS629050(90)**	**Dasatinib**	CRBN
10	**SIAIS629051(91)**	**Dasatinib**	CRBN
11	**BT1(92)**	**Dasatinib**	RNF114
12	**Azo-PROTAC-4C(93)**	**Dasatinib**	CRBN

#### FAK

Focal adhesion kinase (FAK) is a cytoplasmic non-receptor protein tyrosine kinase, which is a member of the focal adhesion complex family.^155,156^ It mediates multiple signaling pathways, such as PI3K/AKT and RAS/MAPK, and also plays an important role in cell invasion and metastasis. FAK exerts kinase-dependent enzyme function and kinase-independent scaffold function which can’t be investigated with kinase inhibitors.^157^ The emergence of PROTACs technology opens a new door for studying the non-enzymatic function of FAK.

**Fig. 22:**
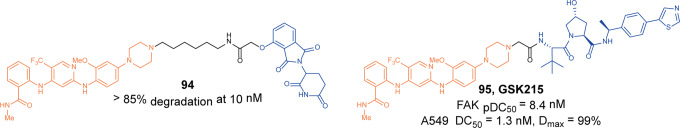
The representative PROTACs targeting FAK

In 2020, Gray group published the patent on FAK degraders.^158^ The patent showed that they designed a large number of FAK degraders based on FAK inhibitor **VS-4718** and different E3 ligase ligands. In the subsequent activity screening, they found that most of the compounds have good FAK-degradation activity. Among them, the degrader **94** (Fig. 22) had relatively potent FAK-degradation activity, which could induce degradation of more than 85% FAK protein at 10 nM.

Recently, GSK has also developed a new type of potent and selective degrader **95** (**GSK-215**, Fig. 22) based on **VS-4718** and VHL ligand.^159^ Interestingly, it was confirmed that degrader **95** (**GSK-215**) which possessed a short and rigid linker generated a highly cooperative ternary complex by SPR and X-ray crystallography data. It induced the degradation of FAK with DC_50_ of 1.3 nM in A549 cells and induced cell proliferation inhibition in A549, MCF-7 cells rather than BT474 cells. The levels of other proteins would also be affected when the concentration was increased to 100 nM, such as CDK7, RPS6KA3, MET, and GAK. In addition, the degrader **95** (**GSK-215**) induced fast, effective, and durable FAK degradation in vivo in mouse liver.

#### MDM2

p53 is an important tumor suppressor which can promote the apoptosis of cancer cells and prevent the development of tumors. Nearly 50% of human cancers are related to the abnormal activity of p53. The interaction between p53 and MDM2 is the main factor affecting the biological activity of p53.^160^ MDM2 is one of the key inhibitors of p53. It is highly expressed in a variety of tumors and plays an important role in the occurrence and development of tumors. Overexpression of MDM2 can downregulate the expression of p53, and inhibiting or degrading the MDM2 protein can block the MDM2–p53 interaction and upregulate the expression of p53, thereby exerting antitumor activity.^161^ Therefore, the development of antitumor drugs targeting MDM2–p53 has become one of the important methods to treat tumors. Although several MDM2–p53 inhibitors have entered clinical trials, no drugs have been approved for clinical use.

**Fig. 23:**
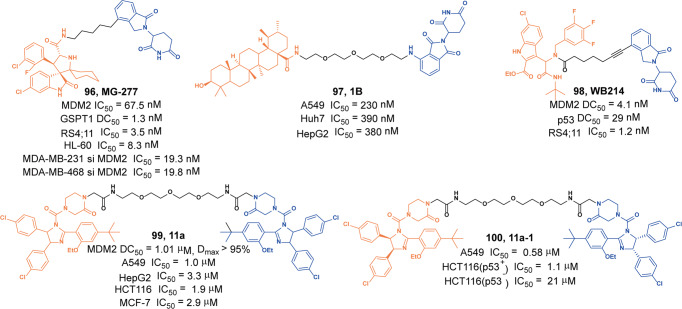
The representative PROTACs targeting MDM2

In 2018, Wang group reported the potent MDM2 degrader **MD-224**. Based on the structure of **MD-224**, they designed and synthesized other **MD-224** analogs in 2019. They found that the degrader **96** (**MG-277**, Fig. 23) was not only a degrader for MDM2, but also a molecular glue for inducing degradation of GSPT1.^162^ The degrader **96** (**MG-277**) only induced moderate degradation of MDM2, but had very good degradation activity of GSPT1 with DC_50_ of 1.3 nM. It could not activate wild-type p53, but it had good antiproliferative activity in a variety of cells. For example, in RS4;11 and HL-60 cells, its IC_50_ were 3.5 nM and 8.3 nM, respectively. Subsequently, they also tested the antiproliferative activity of the degrader **96** (**MG-277**) in MDA-MB-231 *si*MDM2 and MDA-MB-468 *si*MDM2 cell lines, which IC_50_ were 19.3 nM and 19.8 nM, respectively, indicating that the degrader **96** (**MG-277**) exerted antiproliferative activity not only through the MDM2-p53 pathway, but also played a related physiological role through the degradation of GSPT1.

In 2021, Wang group designed and synthesized a series of degraders based on ursolic acid(UA) and pomalidomide.^163^ During the screening process, they found that the degrader **97** (**1B**, Fig. 23) had significant inhibitory activity in different tumor cells. The IC_50_ values in A549, Huh7, and HepG2 were 230, 390, and 380 nM, respectively. Then they conducted mechanism studies and the western blotting results showed that the degrader **97** (**1B**) induced the significant degradation of MDM2 and promoted the expression of P21 and PUMA proteins thereby inhibiting the proliferation of tumor cells and promoting the apoptosis of A549 cells. This was the first research on MDM2 degraders designed based on natural products, which provided ideas for the development of MDM2 degraders.

In 2021, Tang group reported a series of MDM2 ligands which were designed and synthesized based on the four-component Ugi reaction, and then synthesized MDM2 degraders based on these ligands.^164^ After extensive optimization based on antiproliferative activity and MDM2-degradation activity, the degrader **98** (**WB214**, Fig. 23) was determined to be the degrader with the best MDM2-degradation activity in leukemia cells. In RS4;11 cells, the MDM2-degradation activity DC_50_ was 4.1 nM. In addition, they also found that the degrader **98** (**WB214**) also induced the degradation of p53 with DC_50_ of 29 nM. Further studies have shown that it was a molecular glue that induced the degradation of MDM2. At the same time, it could effectively induce the degradation of GSPT1 and showed strong proliferation inhibitory activity on cells. In RS4;11 cells, its inhibitory activity IC_50_ was 1.2 nM.

Also in 2021, Sheng group reported the homo-PROTAC designed based on MDM2 inhibitor **Nutlin-3** derivatives targeted degradation of MDM2.^165^ Since MDM2 was an E3 ligase, when MDM2 inhibitors were used as ligands, they could not only target MDM2, but also could combine with E3 ligase. The results showed that the degrader **99** (**11a**, Fig. 23) could effectively induce the dimerization of MDM2 with highly competitive binding activity and induce the degradation of MDM2 protein in A549 cells. The DC_50_ and *D*_max_ were 1.01 µM and 95%, respectively. At the same time, it showed good antiproliferative activity on a variety of tumor cells. However, because the degrader **99** (**11a**) was a compound with chiral centers, they purified enantiomer **100** (**11a-1**, Fig. 23) and found that its antiproliferative activities in tumor cells were significantly better than that of degrader **99** (**11a**). It was found enantiomer **100** (**11a-1**) showed strong antitumor activity in vivo in the A549 xenograft nude mouse model.

### JAK/STAT signaling pathway-related proteins

#### FLT3

FMS-like tyrosine kinase 3 (FLT3) is a type III receptor tyrosine kinase that regulates hematopoiesis. It is expressed on the surface of many hematopoietic cells and is essential for the normal development of hematopoietic stem cells and hematopoietic cells. After FLT3 binds to ligands, it will dimerize or autophosphorylate and activate JAK-STAT, PI3K, and MAPK signaling pathways, which can promote tumor cell proliferation and differentiation or inhibit tumor cell apoptosis.^166^ FLT3 is expressed in most AML patients and exists 30% mutations. The mutations of FLT3 mainly include internal tandem duplication alteration(FLT3-ITD) and point mutations in the tyrosine kinase domain, accounting for 25% and 5%, respectively. Mutation or high expression of FLT3 may cause the continuous activation of the protein, leading to acute myeloid leukemia and acute lymphocytic leukemia.^167^ There are currently eight drugs that can act on FLT3 have been approved, but these drugs have poor selectivity and can cause gastrointestinal intolerance, long-term cytopenias, hand-foot syndrome and other side effects.^168^ In addition, the single drug has limited efficacy in AML patients with FLT3 mutations. So it is particularly important to use new technologies to develop treatment targeting FLT3 and mutations.

**Fig. 24:**
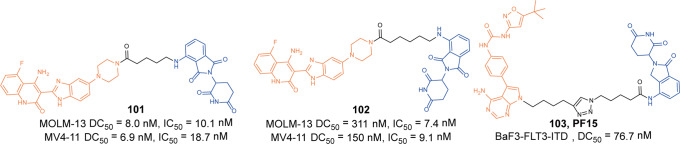
The representative PROTACs targeting FLT3

In 2021, Yang group obtained a series of PROTACs based on the binding model of **Dovitinib** and FLT3.^169^ After screening in vitro antiproliferative activity, it was found that the degrader **101** (Fig. 24) and **102** (Fig. 24) had significant antiproliferative effects on MOLM-13 and MV4-11 cells (FLT3-ITD-positive AML cells), which were better than the inhibitor **Dovitinib**. Subsequently, they tested the degradation activities of these two degraders on FLT3 in MOLM-13 and MV4-11 cells, and the results showed that both the two degraders could induce the efficient degradation of FLT3 protein. The DC_50_ of the degrader **101** to FLT3 in MOLM-13 and MV4-11 cells were 8.0 nM and 6.9 nM, respectively. While the DC_50_ of the degrader **102** in these two cells were 311 nM and 150 nM, respectively. In addition, they also proved that these two degraders completely block the downstream signaling pathways at low concentrations. And in the mouse model of vein transplantation, the degrader **101** and **102** could also show a significant inhibitory effect on the proliferation of MV4-11 cells in vivo.

Also in 2021, Chen group also reported the FLT3 degrader **103** (**PF15**, Fig. 24).^170^ They found **103** (**PF15**) could induce the degradation of FLT3 in BaF3-FLT3-ITD cells with DC_50_ of 76.7 nM.

#### JAK

Janus kinase (JAK) is a family of non-receptor tyrosine kinases, including JAK1, JAK2, JAK3, and Tyk2. Signal and activator of transcription (STAT), the substrate of JAK, dimerizes after being phosphorylated by JAK and then crosses the nuclear membrane into the nucleus to regulate the expression of related genes. JAK-STAT pathway is a major signal transduction mechanism of various cytokines and growth factors and has been implicated in a multitude of diseases from cancer to inflammatory diseases.^171^ Given the importance of the JAK-STAT pathway, blocking the function of JAK can silence the entire pathway, which has important implications for scientific research and disease treatment. Although several JAK kinase inhibitors have entered clinical research, they are usually difficult to achieve selectivity due to the high homology of JAK family proteins.^172^ Therefore, the rise of PROTACs technology provides a new strategy for the study of JAK proteins.

**Fig. 25:**
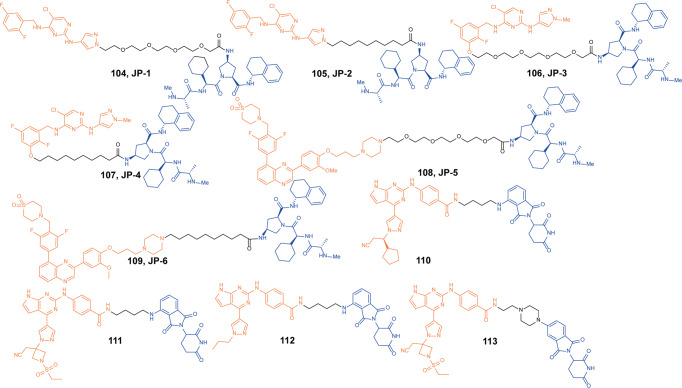
The representative PROTACs targeting JAK

In 2020, GSK developed the first-in-class PROTACs targeting JAK proteins based on two pan-inhibitors which have pyrimidine and quinoxaline scaffold, respectively.^173^ They then tested the degradation activities of these degraders to endogenous JAK1 and JAK2 in THP-1 cells through automated western blotting. They found that the degraders **104**–**109** (**J1-J6**, Fig. 25) were able to induce the degradation of JAK1 and JAK2, which showed no selective degradation. And JAK3 protein was not detected in these cells. The results also proved that the PROTACs bearing an IAP E3 ligase ligand could induce significant degradation of JAK1 and JAK2 in THP-1 cells while VHL and CRBN-based PROTACs could not.

In 2021, Mullighan group designed and synthesized a large number of PROTACs based on the binding modes of **Ruxotinib** and **Barictinib** with JAK protein.^174^ Through protein degradation and MTT experiment screening, it was found that most of the compounds had good JAK2 kinase degradation activity, but they also had certain GSPT1 degradation activity, especially the degrader **110** (Fig. 25) and **111** (Fig. 25). In order to minimize the molecular weight and polar surface area of PROTAC and improve its cell permeability, they also synthesized the N-propyl analog degrader **112** (Fig. 25). And in order to avoid GSPT1 degradation, degrader **113** (Fig. 39) was also designed and synthesized. The degradation activities of these degraders were tested on MHH-CALL-4 cells. They found that the degrader **110** and **111** could induce the degradation of JAK1/2/3, and also had good degradation activity on GSPT1/IKZF1. The degrader **112** could induce the degradation of JAK1/3 and also had good degradation activity for GSPT1/IKZF1, while the degrader **113** could only induce the degradation of JAK2.

Subsequently, they tested the antiproliferative activity of these degraders. They found that the activity of the degraders was better than that of the corresponding inhibitors. At the same time, the activity of the degrader **113** with better selectivity was significantly worse than that of degrader **110**–**112**, which indicated that the antiproliferative activity was not only due to Janus kinase degradation. Finally, they tested the inhibitory activity of degraders in ALL tumor cells derived from different patients. These cells were in different genetic recombination backgrounds, including CRLF2r, EPORr, JAK2r, IL7R/SH2B3 mutations, and so on. Most tumors were sensitive to degraders but not sensitive to the corresponding inhibitors, and the inhibitory activities of the degrader **113** were relatively poor.

#### STAT3

Signal transducer and activator of transcription 3 (STAT3) is a member of the STAT family of transcription factors that replicates and transmits signals from cell surface receptors to the nucleus.^175^ The continuous activation of STAT3 is often associated with the poor prognosis of human cancer because the activated STAT3 signal can not only promote the growth, survival, and metastasis of tumor cells but also inhibit the antitumor immune response.^176^ Therefore, STAT3 is an attractive target for the treatment of human cancer and other human diseases. Although scientists have been working tirelessly on this target for 20 years, targeting STAT3 is still very challenging.

**Fig. 26:**
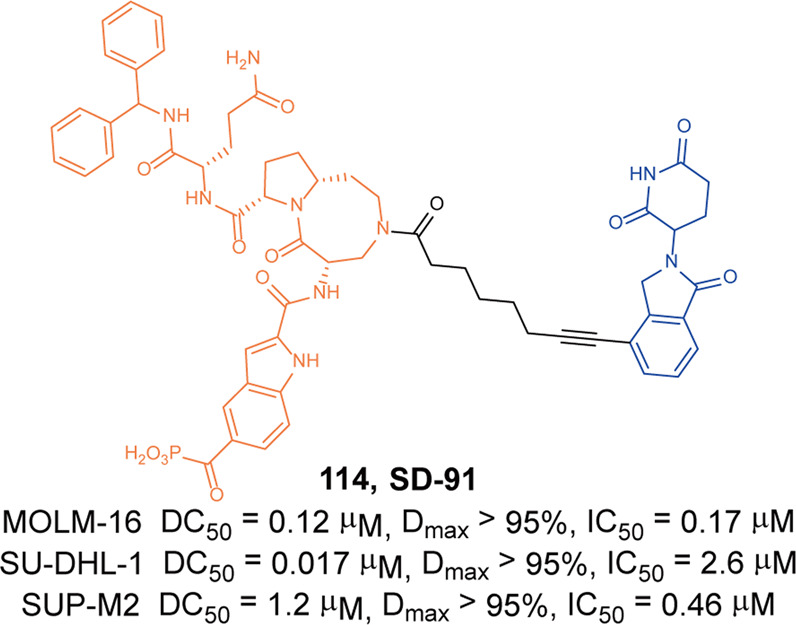
The representative PROTAC targeting STAT3

In 2021, Wang group developed a new STAT3 degrader **114** (**SD-91**, Fig. 26),^177^ which was generated by the *gem*-difluoride of a previously reported STAT3 degrader **SD-36**^178^ convert into a ketone. The degrader **SD-36** could convert into degrader **114** (**SD-91**) in the cell culture media rapidly, with nearly 50% of conversion observed at 2 h and 90% of conversion at 24 h. However, the degrader **114** (**SD-91**) exhibited excellent stability in the cell culture media and the in vivo dosing vehicle. The degrader **114** (**SD-91**) bound to STAT3 protein with a high affinity, and was more potent than **SD-36** in inducing degradation of STAT3 in SU-DHL-1 and MOLM-16 lymphoma cells. A single administration of the degrader **114** (**SD-91**) could selectively and continuously reduce STAT3 in tumor tissues. Moreover, the degrader **114** (**SD-91**) achieved complete and long-lasting tumor regression in the MOLM-16 xenograft model in mice even with weekly administration. The degrader **114** (**SD-91**) represented a promising STAT3 degrader for the treatment of human cancers and other diseases related to STAT3 overactivation.

### Wnt/β-catenin signaling pathway-related proteins

#### β-Catenin

Among many signal pathways, the aberrant activation of Wnt/β-catenin signal is the most important for the occurrence and development of various cancer.^179^ More than 80% of cancer patients have inactivating mutations of APC or activating mutations of CTNNB1, leading to the messenger molecule β-catenin protein continuing to accumulate in cells, which eventually leads to the excessive activation of Wnt/β-catenin signal and the proliferation of cancer cells.^180^ As β-catenin is the central player of the canonical Wnt/β-catenin signaling and is frequently mutated in cancers, it is the most attractive target for cancer therapy.^181^ However, β-catenin has no enzymatic activity and small-molecule binding pockets, and has a large interaction interface with other proteins. Therefore, it is difficult for such small molecule inhibitors to completely inhibit the function of β-catenin.^182^ Thus, inducing the degradation of β-catenin is an ideal method for treating Wnt/β-catenin signal-related diseases.

**Fig. 27:**
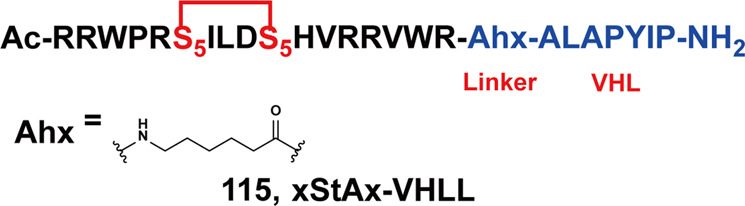
The representative PROTAC targeting β-catenin

Based on the Axin-derived peptide that binds to β-catenin, a stapled peptides **xStAx** was reported to impair Wnt/β-catenin signaling.^183^ Recently, β-catenin-targeting degrader **115** (**xStAx-**VHL**L**, Fig. 27) was developed by coupling **xStAx** with the VHL ligand to achieve efficient β-catenin degradation.^184^ Although they had clinical limitations as peptide-based PROTACs, they still displayed long-term degradation of β-catenin and strong inhibition of Wnt/β-catenin signaling in cancer cells and in APC/organoids. Furthermore, the degrader **115** (**xStAx-**VHL**L**) could effectively restrain tumor formation in BALB/C nude mice and potently inhibit the survival of colorectal cancer patient-derived organoids. In the future, it would be worthwhile to develop a β-catenin-targeting degrader with good pharmacodynamics for clinical therapies.

#### FOXM1

The forkhead box superfamily is a class of transcription factors with a specific “winged helix structure” DNA binding domain, including several families such as FOXA, FOXC, FOXM, FOXO, and FOXP.^185^ FOXM1 (forkhead box protein M1) is one of the key genes controlling cell proliferation and its abnormal activation is closely related to the proliferation and division of cancer cells. It is generally upregulated in laryngeal cancer, gastric cancer, ovarian cancer, etc.^185–188^ And tumor gene expression profiling analysis also confirmed that FOXM1 is one of the most frequently upregulated genes in human malignant tumors,^189^ so it has become an important target for drug development.

**Fig. 28:**
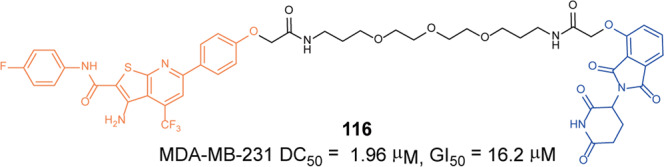
The representative PROTAC targeting FOXM1

Xiang research group synthesized a potent PROTAC degrader **116** (Fig. 28) based on the binding mode of FOXM1 inhibitor **FDI-6.**^190^ They proved that degrader **116** could induce the degradation of FOXM1 protein in MDA-MB-231 cells. The DC_50_ was 1.96 µM and GI_50_ for cell proliferation inhibition was 16.2 µM, which was slightly better than the corresponding inhibitor. This was the first time to use PROTAC technology to induce the degradation of FOXM1.

### GPCR-related proteins

#### α_1A_-AR

Adrenergic receptors (AR) are divided into two categories: α subtype and β subtype. α adrenergic receptor is a type of G-protein-coupled receptor, mainly including α_1_ and α_2_, which mediate mostly excitatory function. Among them, α_1_-ARs play an important role in many physiological processes, including smooth muscle contraction, myocardial time-varying force, liver glycogen metabolism and so on.^191,192^ α_1_-ARs are divided into three subtypes: α_1A_, α_1B_, and α_1D_. α_1A_-AR is crucial in maintaining basic blood pressure,^193^ α_1B_-AR is of significance in the regulation of blood pressure by catecholamines,^194^ and α_1D_-AR is important in both physiological responses.^195^ Therefore, α_1_-ARs are important targets for the treatment of hypertension,^196^ benign prostatic hypertrophy,^197^ prostate cancer^198^ and other diseases. Especially, due to the fact that the α_1A_-AR has a close relationship to the proliferation of prostate cancer cells.^199,200^ Therefore, the development of α_1A_-AR selective degrader can provide a new method for the treatment of prostate cancer.

**Fig. 29:**
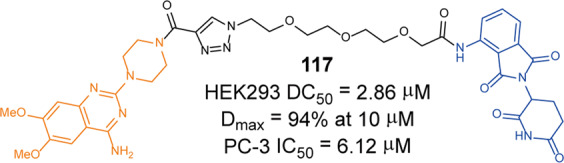
The representative PROTAC targeting α_1A_-AR

In 2020, Li group developed the α_1A_-AR degrader **117** (Fig. 29) based on 4-amino-6,7-dimethoxy-2-(piperazin-l-yl)-quinazoline core of prazosin derivatives,^201^ which endured antagonism to α_1_-ARs. This was the first PROTAC for GPCRs. The PROTAC was conjugated with prazosin and pomalidomide through different linkers. These PROTACs induced the slight degradation of α_1A_-AR in α_1A_-AR stably transfected HEK293 cells. And compound **117** showed the best degradation activity with the drug treatment for 12 h, and the DC_50_ value approximately was 2.86 µM in the treated HEK293 cells. For the HEK293 cells treated with 10 µM compound **117** for 12 h, the *D*_max_ reached up to 94%. Then they test the antiproliferative activity of the compound **117** in androgen-independent PC-3 prostate cancer cells. They found that the antiproliferative activity of **117** (IC_50_ = 6.12 µM) was better than that of **Prazosin** (IC_50_ = 11.72 µM) in PC-3 cells. At last, they found compound **117** resulted in an inhibition in tumor growth when they conducted an in vivo study to examine its influence.

### Epigenetics-related proteins

#### BRD

Lysine acetylation in histone tails has been associated with epigenetic. Bromodomian are highly conserved module which recognize acetylated lysine, it has been shown that 46 proteins contain 61 unique bromodomian so far.^202^ Most of them play important role in transcriptional regulation and chromatin remodeling and have been widely studied as a key target for the treatment of cancer.

The bromo and extral terminal domain family (BET) proteins, which include BRD2, BRD3, BRD4 and testis-specific BRDT, are the most widely studied class of bromodomain-containing proteins. In recent years, a large number of inhibitors targeting BET proteins have been reported,^203–208^ most of them showed good tumor suppression and partial compounds enter clinical studies successfully. Since the first degrader **dBET1** was reported by Brander group in 2015,^2^ a number of PROTACs targeting BET proteins have been developed. However, due to the high homology of BET proteins, it is still difficult to achieve the selectivity of BET protein subtypes.

**Fig. 30:**
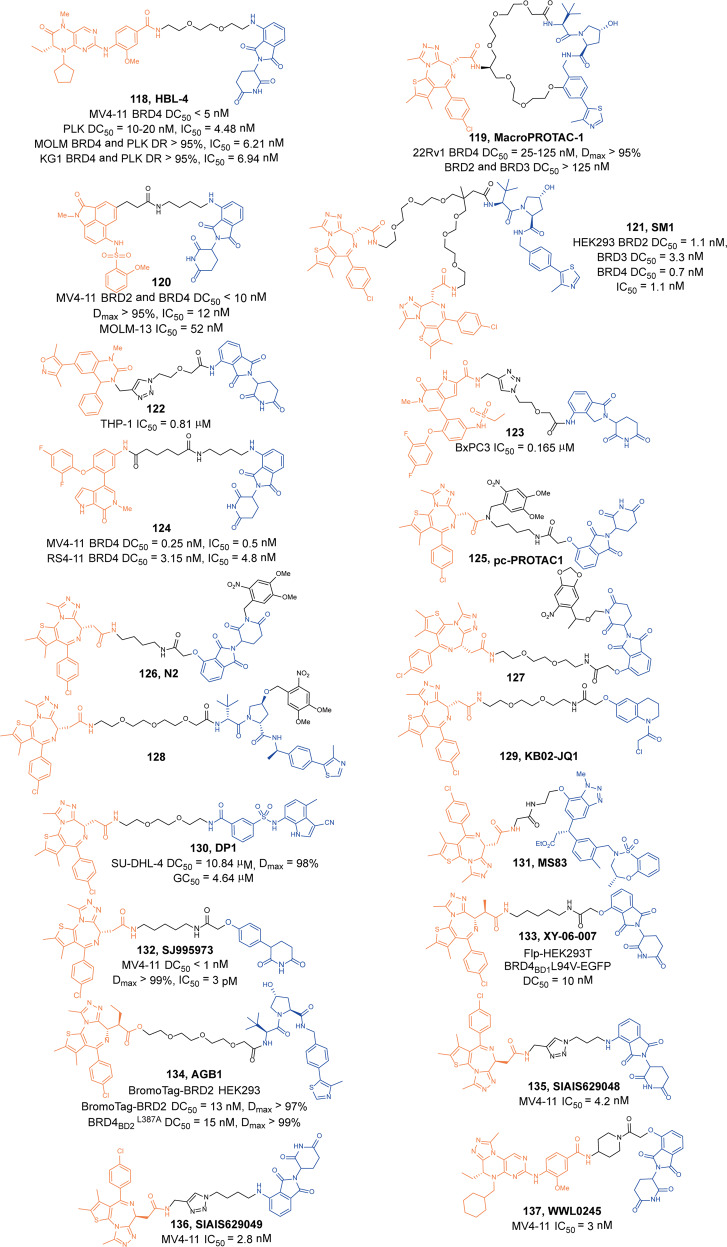
The representative PROTACs targeting BRD

Since Polo-like kinase 1(PLK1) and bromodomain 4(BRD4) are both attractive therapeutic targets in acute myeloid leukemia (AML), Lu group developed BRD4 and PLK1 degrader **118** (**HBL-4**, Fig. 30) based on a dual-target inhibitor **BI2536.**^209^ The degrader **118** (**HBL-4**) induced efficient and fast degradation in human acute leukemia cells tested in vitro and vivo, such as MV4-11, MOLM-13, and KG1. It exhibited potent anti-proliferation in these cells and more effectively suppresses c-Myc levels than inhibitor **BI2536**. Meanwhile, the degrader **118** (**HBL-4**) induced dramatically improved efficacy in the MV4-11 tumor xenograft model compared with **BI2536**, which may be a potential therapy for the treatment of acute leukemia and other types of tumors.

In 2019, Ciulli group introduced macrocyclization into PROTACs based on the structure of reported BET degrader **MZ1**. Macrocyclization could restrict the molecule in its bioactive conformation to reduce the energetic penalty, which has been widely used as a strategy to develop drug selectivity. They synthesized the degrader **119** (**MacPROTAC-1**, Fig. 30) which induced more obvious affinity discrimination between BD1 and BD2 to the BET degrader **MZ1** by adding a cyclizing linker and solved the co-crystal structure of VHL-**119** (**MacPROTAC-1**)-BRD4 BD2.^210^ Compared with **MZ1**, the degrader **119** (**MacPROTAC-1**) showed similar degradation activity and inhibition of cell proliferation in BET-sensitive human prostate carcinoma 22RV1 cells, although the binding affinity to BRD4 decreased.

Despite the pan-inhibitors or degraders targeting BET proteins have achieved great success in treating tumors, lack of subtybe selectivity limits their applications as biological tools and anticancer drugs which could lead unwanted side effects or toxicity. In 2020, Chen group reported degrader **120** (Fig. 30) which achieved potently and selectivity for BRD2 and BRD4 over BRD3 derived from a BD1 selective inhibitor and thalidomide.^211^ The degrader **120** completely induced the degradation of BRD4 at 1 μM with 8h treatment and effectively inhibited cell growth with low cytotoxic effect in both hematoma and solid tumor cells, especially for acute myeloid lymphoma cells MV4-11.

Recently, Ciulli group designed trivalent PROTACs consisting of a bivalent BET inhibitor and an E3 ligand tethered via a branched linker.^212^ Compared to bivalent PROTACs, the most active degrader **121** (**SM1**, Fig. 30), a VHL-based trivalent degrader, displayed more sustained and higher degradation efficacy for BET family proteins which led to more potent anticancer activity. Mechanistically, the degrader **121** (**SM1**) intramolecularly engaged BD2 and BD1 to form a 1:1:1 ternary complex with VHL and BRD4 with prolonged residence time. Although the molecular weight of the trivalent degrader had increased, the degrader **121** (**SM1**) exhibited better cell permeability and the remarkably favorable PK profile.

Besides several common structures such as **JQ1**, more and more BET inhibitors have been used in the design and synthesis of PROTACs. In 2019, Zhang group developed a new class of degrader based on a potent dihydroquinazolinone-based BRD4 inhibitor.^213^ Among them, the best degrader **122** (Fig. 30) completely induced the degradation of BRD4 at 1 μM with 3h treatment and inhibited cell growth with an IC_50_ of 0.81 μM in THP-1 cells, which was four-folds more potent than inhibitor in the antiproliferative assay.

Subsequently, **ABBV-075** derivative was used to design PROTACs by Zhang group^214^ and Yu group^215^ successively. In 2020, Zhang group introduced linkers at the position of pyrrole ring to obtain the degrader **123** (Fig. 30) which induced the degradation of BRD4, cell cycle arrest and apoptosis effectively in human pancreatic cancer cell line BxPC-3. Antiproliferative activity of the degrader **123** against BxPC-3 cell line (IC_50_ = 0.165 μM) was improved about seven-folds compared with **ABBV-075**. In 2021, the sulfonyl group of **ABBV-075** was removed to connect with E3 by Yu group and led to the discovery of the degrader **124** (Fig. 30) which induced the degradation of BRD4 with DC_50_ of 0.25 nM and 3.15 nM in MV4-11 cells and RS4-11 cells. It also induced the proliferation inhibition in MV4-11 and RS4-11 cells with IC_50_ of 0.5 nM and 4.8 nM, respectively.

Given the important role of BET protein in cells, PROTACs targeting BET will inevitably cause toxic effects on normal cells, which limit the clinical application of BET degraders. So there are some strategies were adopted to make PROTACs regulated in space and time.

The introduction of a photocaged group into PROTACs is a common method. In 2019, Pan group introduced the bulky 4,5-dimethoxy-2-nitrobenzyl (DMNB) group into **dBET1** through the amide nitrogen of the linker to get the degrader **125** (**pc-PROTAC1**, Fig. 30) which induced the degradation of BRD4 in live cells and the expected changes of phenotypic in zebrafish only after light irradiation.^216^ As known that methylation of the imide nitrogen abolished degradation activity of a CRBN-based BRD4 degrader, Li group also installed DMNB groups on the glutarimide nitrogen of **dBET1** to obtain the degrader **126** (**N2**, Fig. 30), which was proved to induce the degradation of BRD4 in HEK293T cells and inhibit tongue squamous cell carcinoma(TSCC) HN-6 cells growth in a zebrafish xenograft model.^217^ Subsequently, Deiters group successfully installed a 6-nitropiperonloxymethyl (NPOM) group into the glutarimide nitrogen to generate the BRD4 degrader **127** (Fig. 30), which induced the potent degradation of BRD4 by treatment with 365 nm light in HEK293T cells.^218^ And the DMNB group was attached to the hydroxyl group of VHL E3 ligase-recruiting ligand by Tate group in 2020.^219^ The degrader **128** (Fig. 30) could induce the degradation of BRD4 after a short irradiation time under the live-cell video microscopy.

It is well-known that more than 600 E3 ubiquitin ligases are encoded by the human genome, only a handful of E3 ligases(VHL, CRBN, IAPs, and MDM2) could be used by PROTACs technology. BRD4 is a commonly used target for screening new E3 ligases for PROTACs. In 2019, Cravatt group has validated that the E3 ligase subunit DCAF16 supported targeted protein degradation by synthesizing degrader **129** (**KB02-JQ1**, Fig. 30) which induced the degradation of BRD4 with the engagement of DCAF16.^220^ However, **KB02-JQ1** could only induce the degradation of BRD4 at a concentration of 40 μM. In 2020, Chen group also developed a BRD4 degrader **130** (**DP1**, Fig. 30) based on JQ1 and DCAF15 ligand **E7820.**^221^ The results showed that **130** (**DP1**) was the most potent degrader with DC_50_ of 10.84 μM and *D*_max_ of 98% in SU-DHL-4 cells. Recently, Jin group synthesized the degrader **131** (**MS83**, Fig. 30) by connecting a highly potent, selective, and noncovalent KEAP1 ligand **KI696** to the BET bromodomain pan-inhibitor (+)-**JQ1**.^222^ Interestingly, the degrader effectively induced the degradation of BRD4 and BRD3 protein levels, but did not have an influence on BRD2 protein and selectively induced the degradation of BRD4 short isoform over long isoform in MDA-MB-231 cells. The CRBN-based PROTACs are known to be inherently unstable, readily undergoing hydrolysis in body fluids, which significantly affects their cell efficacy. Recently, Rankovic group developed novel CRBN binders, phenyl glutarimide (PG) analogs, which retained high affinity for CRBN and displayed improved chemical stability.^223^ Based on this novel ligand, their group synthesized a novel BRD4 degrader **132** (**SJ995973**, Fig. 30) which inhibited the MV4-11 cells proliferation with an IC_50_ value of 3 pM. Moreover, the degrader **132** (**SJ995973**) exhibited the most potent degradation efficacy with DC_50_ of 0.87 nM in MV4-11 cells.

In 2021, Fischer group developed a series of novel CRBN-based BRD4_BD1_L94V PROTAC based on a “bump-and-hole” approach.^224^ By using cells stably expressing the BRD4_BD1_L94V degron fused to EGFP systems, the corresponding degrader **133** (**XY-06-007**, Fig. 30) bound to BRD4_BD1_L94V with increased selectivity over the wild-type bromodomain, and exhibited potent and selectively degradation of BRD4_BD1_L94V with no degradation of wild-type and other BET family of bromodomains. In addition, the degrader **133** (**XY-06-007**) displayed acceptable pharmacokinetics profiles, which made it suitable candidates for future in vivo studies. In recently, Ciulli group also developed a novel VHL-based degrader **134** (**AGB1**, Fig. 30) based on the “bump-and-hole” approach.^225^ In an inducible BromoTag degron system, the degrader **134** (**AGB1**) not only formed a strong, cooperative ternary complex between VHL and the BromoTag-BRD2 but also completely induced the degradation of BromoTagged target proteins with low nanomolar potency. The degrader **134** (**AGB1**) exhibited exquisite selectivity over the native wild-type BET, which led it not cytotoxic in several cancer relevant cell lines. In summary, these two distinct methods provided a useful tool to study the effect and implications of rapid and highly selective degradation of a target protein.

Also in 2021, Jiang group reported the efficient construction of an IMiD-based azide compound library and applied this method to the development of BET protein degraders. The BET protein degraders **135** (**SIAIS629048**, Fig. 30) and **136** (**SIAIS629049**, Fig. 30) were obtained.^152^ These two degraders showed strong BET protein-degradation activity when the concentration was 50 nM. At the same, it could show strong anti-proliferation inhibitory activity on MV4-11 cells. Recently, Wang group reported the selective BRD4 degrader **137** (**WWL0245**, Fig. 30) with potent antiproliferative effects in AR-positive prostate cancer based on a dual BET/PLK1 inhibitor **WNY0824**^226^ The degrader **137** (**WWL0245**) could effectively induce the degradation of BRD4 and the *D*_max_ was more than 99% in AR-positive prostate cancer cells. It had no degradation activity against other BRD subtype proteins. The degrader **137** (**WWL0245**) also showed good antiproliferative activity in BETi-sensitive cancer cells (including AR-positive prostate cancer cells) with an IC_50_ of 3 nM in MV4-11 cells.

Finally, we compared the reported BRD degraders (Table 5). It was found that although there were various types of BRD warheads currently used, **JQ1** was used more frequently and others were used less. In the selection of E3 ligases, most of the currently used were CRBN and VHL, and some new E3 ligases have been mentioned in reports, such as DCAF15, DCAF16, etc. These cases were all in the development of new E3 ligases reportes. Therefore, more BRD degraders based on different BRD inhibitors need to be developed.

**Table 5:** The summary and comparison of PROTACs targeting BRD

No.	PROTAC	Warhead	E3 ligase
1	**HBL-4(118)**	**BI2536**	CRBN
2	**MacroPROTAC-1(119)**	**JQ1**	VHL
3	**120**	Benzo[cd]indol-2(1H)-one) derivative	CRBN
4	**121**	**JQ1**	VHL
5	**122**	Dihydroquinazolinone derivative	CRBN
6	**123**	**ABBV-075**	CRBN
7	**124**	**JQ1**	CRBN
8	**pc-PROTAC1(125)**	**JQ1**	CRBN
9	**N2(126)**	**JQ1**	CRBN
10	**127**	**JQ1**	CRBN
11	**128**	**JQ1**	VHL
12	**KB02-JQ1(129)**	**JQ1**	DCAF16
13	**DP1(130)**	**JQ1**	DCAF15
14	**MS83(131)**	**(+)-JQ1**	KEAP1
15	**SJ995973(132)**	**JQ1**	CRBN
16	**XY-06-007(133)**	**JQ1**	CRBN
17	**AGB1(134)**	**JQ1**	VHL
18	**SIAIS629048(135)**	**JQ1**	CRBN
19	**SIAIS629049(136)**	**JQ1**	CRBN
20	**WWL0245(137)**	**WNY0824**	CRBN

#### CBP and p300

The paralogous chromatin regulators CREB-binding protein (CBP) and p300 (also known as KAT3A and KAT3B) maintain gene expression programs through chromatin lysine acetylation and transcription regulation, as well as the scaffold function mediated by multiple protein–protein interaction domains. Several potency, selectivity, and drug-like properties of small-molecule inhibitors targeting these domains have been developed, which provided unique tools for exploring various p300/CBP functions of enhancers.^227^ However, inhibiting a single domain alone cannot completely eliminate the p300/CBP activity in the cell. Thus, it is necessary to inhibit multiple functional domains at the same time, or even completely deplete the protein to ablate the p300/CBP-mediated enhancer activity.^228^

**Fig. 31:**
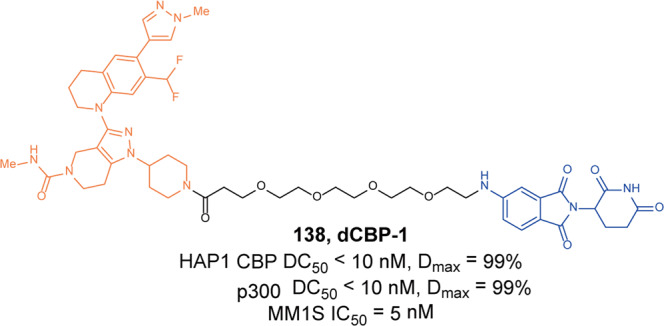
The representative PROTAC targeting CBP and p300

In 2021, Ott group described the first CRBN-based p300/CBP degrader **138** (**dCBP-1**, Fig. 31).^229^ The degrader **138** (**dCBP-1**) induced the highly potent, selective, and rapid dual degradation of CBP and p300, which highlighted the capability of applying PROTAC technology to exceptionally large proteins(>300 kDa). The degrader **138** (**dCBP-1**) had potent antiproliferative activity in multiple myeloma cells. Compared with the treatment of bromodomain and KAT domain inhibitors alone or in combination, it had enhanced effects on MYC gene expression programs, anti-proliferation, and chromatin structure in multiple myeloma. As an effective degrader of this unique acetyltransferase, degrader **138** (**dCBP-1**) was a useful tool to analyze the mechanisms of these factors coordinate enhancer activity in normal and cancer cells.

#### ENL

The YEATS domain is a new type of histone acetylation “reader”. The human genome encodes four YEATS domain-containing proteins, namely ENL, AF9, YEATS2, and GAS41.^230^ Among them, ENL protein is essential for the development of acute myeloid leukemia(AML).^231^ When ENL is knocked out, it would induce anti-leukemia effect and inhibit the growth of leukemia in vivo and in vitro.

**Fig. 32:**
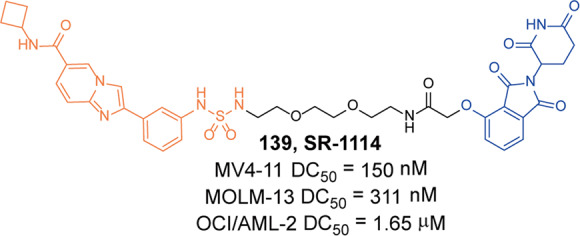
The representative PROTAC targeting ENL

In 2021, Erb group discovered the highly effective ENL YEATS domain inhibitor **SR-0813** based on high-throughput screening technology. On the basis of **SR-0813**, they designed and synthesized a degrader **139** (**SR-1114**, Fig. 32) that targeted ENL.^232^ It was found that the degrader **139** (**SR-1114**) could induce degradation of ENL protein in a variety of cell lines, and it had the best activity in the MV4-11 cells with DC_50_ of 150 nM.

#### HDAC

Histone deacetylases (HDACs) which serve as “epigenetic erasers” play an important role in chromosome structural modification and gene expression regulation by catalyzing deacetylation of substrate proteins. They make histones bind tightly to negatively charged DNA by deacetylating them, which densify chromatin and inhibit gene transcription. Until now, 18 human HDACs have been identified and classified into four classes: Class I (HDAC1, 2, 3, and 8), class II (HDAC 4, 5, 6, 7, 9, and 10), class III (SIRT1, 2, 3, 4, 5, 6, and 7) and class IV (HDAC11).^233–235^ Class I HDAC is mainly located in the nucleus, and HDAC3 is also present in the cytoplasm. Class II can respond to different cell signal responses and shuttle between the nucleus and the cytoplasm. Class III is a completely different type as the atypical histone deacetylase family of other HDACs.^236,237^ Type IV is mainly located in the nucleus.

**Fig. 33:**
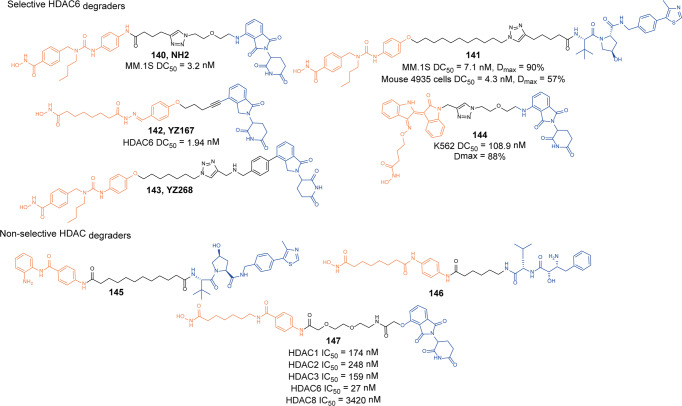
The representative PROTACs targeting HDAC

HDAC6, the only protein in the HDACs family that has two tandem domains, is mainly distributed in the cytoplasm. HADC6 deacetylates α-tubulin, HSP90, cortactin and interacts with many proteins such as dynein, ubiquitin, which make it participate in cancer progression, neurodegenerative diseases, and inflammatory disorders. Rao group has been working on developing PROTACs targeting HDAC6 since 2019,^238^ they linked pomalidomide to the benzene ring of **NexA** unlike **NP8**, which led to the discovery of HDAC6 degrader **140** (**NH2**, Fig. 33) in 2019.^239^ When it was compared with **NP8**, the degrader **140** (**NH2**) exhibited comparably excellent degradation activity in MM.1S cells with the DC_50_ of 3.2 nM. The two degraders extended from different directions of the inhibitor exerted the same degradation activity revealed the extremely large flexibility of the ternary complex.

In 2020, on the basis of previous work, Tang group replaced pomalidomide in the structure of HDAC6 degrader with VHL and obtained the first selective HDAC6 degrader.^240^ Among them, the degrader **141** (Fig. 33) showed the best degradation activity in MM.1S with DC_50_ of 7.1 nM and the *D*_max_ of 90%. In addition, they also tested its HDAC6 degradation activity in mouse cells. In the mouse 4935 cell line, the DC_50_ was 4.3 nM and the *D*_max_ was 57%. Subsequently, they developed a competition assay to evaluate binding affinity of different E3 ligands in cells and screened a library of thalidomide analogs, including those with partial linkers. After screening, the most active E3 ligase ligands were conjugated to pan-inhibitor **SAHA** led to the discovery of a selective HDAC6 degrader **142** (**YZ167**, Fig. 33).^241^ The DC_50_ of the degrader **142** (**YZ167**) was 1.94 nM in MM.1S cells. At the same time, it was found that the degrader **143** (**YZ268**, Fig. 33) designed based on HDAC6-selective inhibitor **Next-A** also had selective degradation activity for HDAC6 during the screening process, without influence on neo-substrate IKZFs and GSPT1.

In 2021, He group also reported another HDAC6 degrader based on CDK/HDAC6 inhibitor derived from Indirubin.^242^ They evaluated the degradation activities of HDAC6 in K562 cells. Interestingly, the PROTAC **144** (Fig. 33) demonstrated the most superior HDAC6 degradation with DC_50_ of 108.9 nM and *D*_max_ of 88%. Also, they found the degrader **144** (Fig. 33) upregulated acetylation of α-tubulin without any obvious degradation of HDAC1 and CDK2 in K562 and HeLa cells.

In addition to these degraders that selectively targeting HDAC6, other research groups have also reported degraders targeting other subtypes of HDAC in 2020. For example, Hodgkinson group reported a degrader **145** (Fig. 33) targeting class I HDAC (HDAC1/2/3) designed based on VHL and HDAC inhibitor **CI-994.**^243^ They found that the degrader **145** could induce the degradation of HDAC1/2/3 in HCT116 cells. Zhang group attempted to design PROTACs targeting HDAC6 based on **Bestatin** and **SAHA** by recruiting cellular inhibitor of apoptosis protein 1(cIAP1) E3 ubiquitin ligase.^244^ However, effective degradation of HDAC1/6/8 could be observed in RPMI-8226 cells. What interesting was that the degradation effect of the degrader was not obvious when the cells were treated for 6 hours. However, the degradation effect on the three subtypes of HDAC was significantly enhanced when the treatment time was extended to 24 hours. The degrader **146** (Fig. 33) exhibited more potent aminopeptidase N (APN, CD13) inhibitory activities and anti-angiogenic activities than the approved APN inhibitor **Bestatin**, which made it work as a potent APN and HDAC dual inhibitor and HDAC1/6/8 degrader instead of just a degrader. The degrader **147** (Fig. 33) was designed by Hansen group based on pomalidomide and **SAHA**.^245^ Although it could show strong inhibitory activity against a variety of subtypes of HDAC, in subsequent experiments they found that the degrader **147** could only induce the degradation of HDAC1 and HDAC6, which proved the difference between the selectivity and affinity activity of degrader. It provided a reference for the subsequent design of selective degraders.

#### KDM5C

Epigenetic plays an important role in various biological functions. They are not only closely related to the normal functions of the human body, but also related to the occurrence and development of many diseases. Therefore, it an effective means that controlling epigenetic changes to treat diseases. Histone methylation is a reversible genetic modification. Histone methylation and demethylation are in a dynamic equilibrium process, which participates in the regulation of gene transcription and other biological events.^246^ The imbalance between histone methylation and demethylation may play an important role in the occurrence and development of tumors. Many histone demethylase inhibitors have been reported, but no drugs have been approved so far.^247^ Therefore, traditional small-molecule histone demethylase inhibitors have insufficient stamina in the treatment of diseases.^248^ So developing degraders targeting histone demethylases based on PROTAC technology has become a feasible solution.

**Fig. 34:**
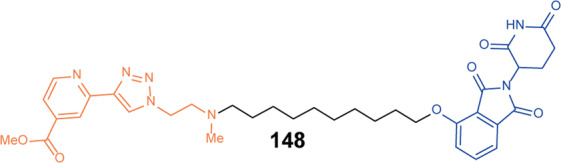
The representative PROTAC targeting KDM5C

In 2021, Suzuki group reported histone demethylase degrader **148** (Fig. 34) designed based on KDM5C inhibitor.^249^ They found that the degrader **148** had good degradation activity in prostate cancer PC-3 cells. When the concentration was 5 µM, obvious protein degradation could be observed. With the increase of drug concentration, the degradation activity was obviously enhanced. It was also proved that the antiproliferative activity of degrader **148** on tumor cells was significantly better than inhibitor. Although the degradation activity was not excellent, as the first case of histone demethylase degrader, it laid the foundation for the subsequent development of related target degraders.

#### NAMPT

Nicotinamide phosphoribosyltransferase (NAMPT) is a cytokine that promotes B-cell maturation and inhibits neutrophil apoptosis.^250^ It is the rate-limiting enzyme that catalyzes the synthesis of nicotinamide adenine dinucleotide (NAD^+^). Since tumor cells have a high demand for large amounts of NAD^+^, inhibiting NAMPT expression can significantly inhibit tumor cell proliferation. It has been confirmed that NAMPT is highly expressed in various malignant tumors such as breast cancer, prostate cancer, gastric cancer, thyroid cancer, colon cancer and hematological tumors.^251^ Therefore, NAMPT is considered as a drug target for antitumor therapy.^252^

**Fig. 35:**
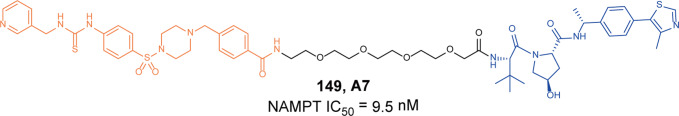
The representative PROTAC targeting NAMPT

Based on the binding mode of inhibitor **MS7** and NAAMPT, a series of PROTAC molecules were obtained by connecting **MS7** with VHL ligands.^253^ Then it was found that **149** (**A7**, Fig. 35) was an effective degrader of NAMPT protein. Intracellular NAMPT (iNAMPT) could be degraded by **149** (**A7**) through the ubiquitin–proteasome system, thereby in turn decreases the secretion of extracellular NAMPT (eNAMPT). At the same time, they also found that the inhibitor **MS7** did not have the function of inhibiting tumor-infiltrating MDSCs, and the degrader could boost antitumor efficacy through this effect, so the degrader **149** (**A7**) exhibited stronger antiproliferative activity than the inhibitor **MS7**.

#### NSD3

Histone lysine methyltransferase catalyzes the transfer of methyl groups to specific lysine side chains at the ends of histones H3 and H4 to form histone methylation marks, thereby affecting gene transcription, DNA replication, and DNA repair. It plays an important role in maintaining chromatin stability and gene expression regulation. Representative lysine methylases include PRDM6, G9a, EZH2, DOT1L, NSD, and SUV420H1.^254^ NSD is a nuclear receptor-binding SET domain protein (NSD) family, including three members of NSD1, NSD2 (also known as MMSET or WHSC1) and NSD3 (also known as WHSC1L1). NSD3 is a Lysine methyltransferase at position 36 of histone H3(H3K36), catalyzes the dimethylation of H3K36.^255^ NSD methyltransferases are mutated, amplified, and overexpressed in a variety of human cancers, including multiple myeloma, acute myeloid leukemia, acute lymphoblastic leukemia, breast cancer, prostate cancer, and lung cancer.^256^ Therefore, NSD is considered as a potential target for the development of novel anticancer drugs.

**Fig. 36:**
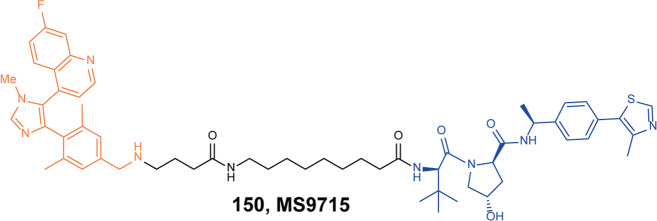
The representative PROTAC targeting NSD3

**BI-9321** is a reported NSD3 PWWP1 antagonist. By analyzing its binding mode with NSD3 protein, Wang group obtained a series of PROTAC molecules by connecting **BI-9321** with different E3 ligase ligands and then screened the degradation activities of NSD3.^257^ They found that **150** (**MS9715**, Fig. 36) had the best NSD3 degradation activity with DC_50_ of 4.9 µM and *D*_max_ of greater than 80% in MOLM-13 cells. After quantitative proteomic analysis, it was found that **150** (**MS9715**) was highly selective and had no degradation activity on other proteins. In addition, **150** (**MS9715**) could effectively inhibit the expression of NSD3 and cMyc-related genes, while the corresponding inhibitor **BI-9321** did not have this function.

#### PRC2 (EZH2, EED)

The polycomb repressive complex 2 (PRC2) is an epigenetic modulator of transcription, which is mainly constituted of four subunits: EZH1/2 (enhancer of the zeste homolog 1/2), EED (embryonic ectoderm development), SUZ12(suppressor of the zeste 12 protein homolog), and RbAp46/RbAp48 (retinoblastoma (Rb)-associated proteins 46/48).^258^ It catalyzes methylation of H3K27. The trimethylation of H3K27 (H3K27me3) is a transcriptionally repressive epigenetic mark that regulates gene expression, and hyper-trimethylation of H3K27 can be observed in several types of tumors. EZH2 is the catalyst subunit of PRC2 and mutation will be happened in several cancers, such as DLBCL.^259^ **EPZ6438** is a selective EZH2 inhibitor approved for epithelioid sarcoma.^260^ In fact, EZH2 also mediates transcriptional activation independent of EZH2/PRC2 catalytic activity in some cancers.

**Fig. 37:**
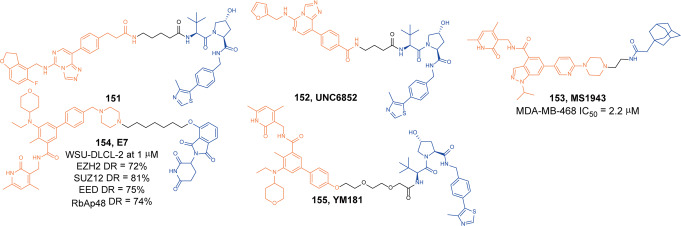
The representative PROTACs targeting PRC2 (EZH2, EED)

In 2019, Bloeche group reported an EED degrader **151** (Fig. 37) by conjugating a potent EED inhibitor **MAK683** with VHL ligand.^261^ The degrader **192** not only induced rapid degradation of EED but also EZH2 and SUZ12 within the PRC2 complex. It selectively inhibited the proliferation of PRC2-dependent cancer cells. At the same time, James group reported that degrader **152** (**UNC6852**, Fig. 37) also reduced the protein level of EED (DC_50_ = 0.79 µM) and EZH2 (DC_50_ = 0.3 µM) on HeLa cells.^262^ In summary, these data demonstrated that the degradation of the subunit of the PRC2 complex could cause the complex to lose its function, which was expected to be a method for PRC2-mediated cancer treatment.

In 2020, Jin group utilized a hydrophobic tag to link an EZH2 inhibitor to generate the degrader targeting EZH2.^263^ The degrader **153** (**MS1943**, Fig. 37) could reduce EZH2 protein levels in MDA-MB-468 cells at 5 µM and suppress the H3K27me3 mark. Compared with inhibitor, the degrader **153** (**MS1943**) showed significant inhibition on cell growth and induced cell death in TNBC cells in vitro and in vivo.

Based on a clinical EZH2 inhibitor **EPZ6438** and pomalidomide, Yu group developed the degrader **154** (**E7**, Fig. 37) which induced degradation efficacies of all PRC subunits(EZH2 72%, SUZ12 81%, EED 75%, RbAp48 74%) at 1 µM in WSU-DLCL-2 cells.^264^ The degrader **154** (**E7**) also displayed stronger anticancer abilities than **EPZ6438** in WSU-DLCL-2 cells and SWI/SNF-mutant cancer cells.

In 2021, Wen group reported a series of degraders with VHL ligands. The degrader **155** (**YM181**, Fig. 37) induced the degradation of 50% EZH2 protein level at 1 μM, and the degradation could be detected at 2 h.^265^ It showed superior antiproliferative effects against lymphoma cell lines.

#### PRMT5

The PRMT family is a group of enzymes that can modify the nitrogen atom of the arginine guanidine group with monomethyl and dimethyl groups. Nine members have been found in mammals. According to the different methylation products, PRMTs can be divided into type I and type II. Both type I and type II enzymes can catalyze the production of monomethylated modification. The difference is that type I can further catalyze the production of asymmetric dimethylarginine (aDMA), while type II catalyzes the formation of symmetric dimethylarginine (sDMA). PRMT5 is the first type II methyltransferase to be isolated as a JAK2 binding protein.^266^ Overexpression of PRMT5 leads to infectious diseases, heart diseases, and cancers(such as breast, lung, and liver cancer).^267–269^

**Fig. 38:**
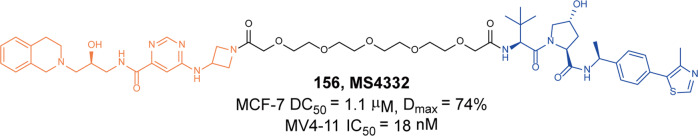
The representative PROTAC targeting PRMT5

In 2020, Jin group developed a degrader **156** (**MS4322**, Fig. 38) conjugated with the PRMT5 inhibitor **EPZ015666** and VHL ligand through a PEG linker.^270^ As the first-in-class PRMT5 degrader, **156** (**MS4322**) induced the efficient degradation of PRMT5 in MCF-7 cells with the DC_50_ and *D*_max_ value of 1.1 µM and 74%, respectively. The antiproliferative activity of the degrader **156** (**MS4322**) (IC_50_ = 18 nM) was better than **EPZ015666** (IC_50_ = 30 nM) in MV4-11 cells. Moreover, it was highly selective for PRMT5 by proteomic study and exhibited good plasma exposure in mice. So the degrader **156** (**MS4322**) was a valuable chemical tool for exploring the PRMT5 functions in disease.

#### SIRT2

Sirtuins protein is a nicotinamide adenine dinucleotide (NAD+) dependent histone deacetylase.^271^ There are seven recognized members in the human sirtuin family: SIRT1-SIRT7, all of which have highly conserved NAD binding domains and catalytic functional domains. The sirtuin protein family can regulate the acetylation modification and ADP ribosyl modification of a variety of proteins. SIRT2 is mainly distributed in the cytoplasm and is particularly important for the growth and metabolism of tumor cells, which makes SIRT2 an attractive target for cancer treatment.^272^

**Fig. 39:**
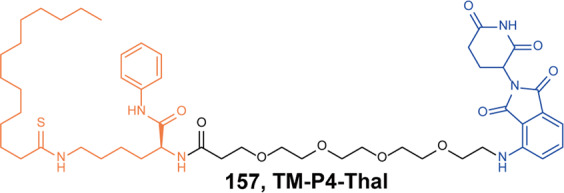
The representative PROTAC targeting SIRT2

In 2020, Lin group developed a SIRT2 degrader **157** (**TM-P4-Thal**, Fig. 39) conjugated with the thiomyristoyl lysine-based SIRT2 selective inhibitor **TM** and CRBN ligand through a PEG linker.^273^ The degrader **157** (**TM-P4-Thal**) successfully induced the degradation of SHP2 at 0.5 µM in MCF-7 cells, which led to simultaneous inhibition of its deacetylase and defatty-acylase activities in living cells.

#### WDR5

The chromatin-associated WD40 repeat domain protein 5(WDR5) acts as a functional subunit of the mixed lineage leukemia (MLL) histone methyltransferase complexes. WDR5 is critical for the methylation of histone H3 lysine 4 (H3K4) on chromatin catalyzed by the MLL1 complex and MLL1 complex-mediated regulations of gene transcription.^274^ Numerous studies have identified WDR5 as a promising potential therapeutic target. Efforts on targeting the WIN or WBM binding site of WDR5 have led to discovery of several inhibitors that potently and selectively block PPIs between WDR5 and its binding partners.^275^ However, these WDR5 PPI inhibitors rely on receptor occupancy pharmacology and target only some but not all WDR5 oncogenic functions, exerting poor antiproliferative activity on tumor cells.^276^

**Fig. 40:**
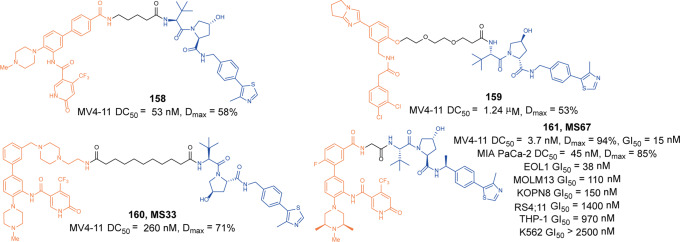
The representative PROTACs targeting WDR5

In 2021, Knapp group reported different WDR5 degraders based on two diverse WIN site binding scaffolds (**OICR-9429** modified scaffold and pyrroloimidazole modified scaffold) and different E3 ligase ligands.^277^ In MV4-11 cells, **OICR-9429** modified scaffold derived degrader **158** (Fig. 40) induced the degradation of WDR5 of 58% with a DC_50_ value of 53 nM, and pyrroloimidazole modified scaffold derived degrader **159** (Fig. 40) induced the degradation of WDR5 of 53% with a DC_50_ value of 1.24 µM.

In the same year, Jin group also reported the WDR5 degraders based on **OICR-9429** and VHL ligase ligands.^278^ Firstly, they designed WDR5 degrader **160** (**MS33**, Fig. 40) and found it could induce the degradation of WDR5 with DC_50_ of 260 nM. Subsequently, they used the degrader **160** (**MS33**) successfully resolve the structure of the VHL-**MS33**-WDR5 ternary complex. Based on the crystal structure, they modified the **OICR-9429** ligand and shortened the linker to obtain a new WDR5 degrader **161** (**MS67**, Fig. 40). They found that the degrader **161** (**MS67**) could quickly induce the degradation of WDR5 with DC_50_ of 3.7 nM, which was about 70-folds higher than the degrader **160** (**MS33**). At the same time, they also resolved the structure of the VHL-**MS67**-WDR5 ternary complex. Subsequently, the antiproliferative activity of the degrader **161** (**MS67**) was tested on a variety of AML cells, which showed that it had good anti-proliferation activity. In addition, it inhibited the malignant growth of MLL-*r* AML cells in vitro and in vivo.

### Cell cycle-related protein

#### Aurora A

Aurora kinases are serine/threonine protein kinases that regulate centrosome and microtubule functions during mitosis. They are mainly divided into three subtypes: Aurora A, Aurora B, and Aurora C. Aurora A and Aurora B are essential for the cell cycle progression of most cell types and Aurora C mainly distributes in testis.^279^ Aurora A kinase is related to centrosome replication and mitotic exit. When Aurora A kinase is inhibited, it can effectively cause cell mitosis to arrest in the G2/M phase and quickly induce apoptosis.^280^ Aurora B kinase plays a role in coordinating the function of chromosomes and cytoskeleton in the cell cycle. When Aurora B kinase is inhibited, the cytoplasm of the cell cannot be divided and cell growth is arrested.^281^ Since both Aurora A and Aurora B kinases are related to tumorigenesis, Aurora kinase inhibitors have been extensively carried out in clinical researches on various diseases, such as lung cancer, breast cancer, esophageal cancer, colorectal cancer, acute myeloid leukemia and so on, but currently no drugs have been approved for clinical use.

**Fig. 41:**
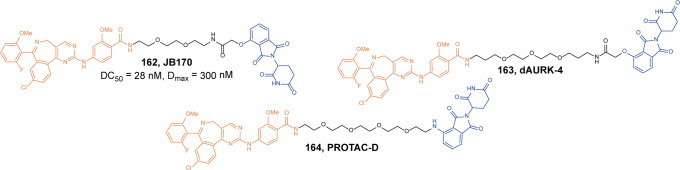
The representative PROTACs targeting Aurora A

The Wolf group reported the first selective Aurora A degrader **162** (**JB170**, Fig. 41) in 2020 based on the inhibitor **alisertib** (**MLN8237**),^282^ which had a known binding mode and high binding affinity with Aurora A kinase and easy derivation. They found that degrader **162** (**JB170**) had strong binding ability and degradation activity to Aurora A kinase, its degradation activity was significantly better than that of other designed molecules, and it had a *D*_max_ of 300 nM and a DC_50_ of 28 nM for Aurora A kinase. After MV4-11 cells were treated with degrader **162** (**JB170**) or **alisertib**, degrader **162** (**JB170**) reduced the level of Aurora A by 73%, which was 57% lower than **alisertib**. And among the 4259 proteins detectable in this experiment, no other proteins were downregulated, including Aurora B. Subsequently, they found that degrader **162** (**JB170**) only induced cell accumulation in S phase, which was different with **alisertib** that induced the G2/M phase arrest of cells. They speculated this may be due to the fact that degrader **162** (**JB170**) had regulated the non-enzymatic activity of Aurora A. In order to study the effect of degrader **162** (**JB170**) on the proliferation activity of cancer cells, they used degrader **162** (**JB170**) to treat MV4-11 cells. The MV4-11 cells were inhibited by 32% after 72 h of treatment with 1 µM of **JB170**. Similar results were observed in the colony formation assay using IMR5 cells.

Fischer group reported the second case of selective Aurora A degrader **163** (**dAURK-4**, Fig. 41) in 2021,^283^ which was also based on **alisertib** as the target protein ligand. They verified the degradation activity by western blot in the MM.1S multiple myeloma cell line, and also proved that the antiproliferative activity of degrader **163** (**dAURK-4**) in the MM.1S multiple myeloma cell line was significantly better than that of the inhibitor **alisertib**.

Lindon group reported the third Aurora A degrader **164** (**PROTAC-D**, Fig. 41) in 2021.^284^ They verified its degradation activity on Aurora A in the U2OS cell lines and also proved that it did not induce Aurora B kinase degradation. At the subcellular level, they found that the distribution of Aurora A on the spindle was different from that of the centrosome. Therefore, the therapeutic phenotypic result of degrader **164** (**PROTAC-D**) was different from the result mediated by **alisertib**. At the same time, they also confirmed that in intermitotic cells, degrader **164** (**PROTAC-D**) mediated the degradation of Aurora A in the non-centrosome, but had no effect on the level of Aurora A in the centrosome.

#### Cdc20

The cell-division cycle protein 20 (Cdc20) is the substrate receptor of anaphase synthesis complex or cyclosome (APC/C). It coordinates late initiation and exit of mitosis through safety and time-dependent degradation.^285^ Cdc20 is a key mitotic factor, which controls the beginning of anaphase and the exit of mitosis. Moreover, Cdc20-APC/C plays a key role in cancer progression and drug resistance. Furthermore, Cdc20-APC/C cell processes beyond the cell cycle, including apoptosis, neurogenesis, stem cell expansion.^286^ Meanwhile, it has a strong connection between the aberrant upregulation of Cdc20 and various types of cancers. Cdc20 is a potential biomarker and an ideal target for cancer treatment.^287^

**Fig. 42:**
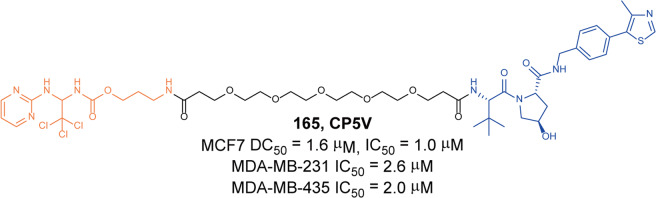
The representative PROTAC targeting Cdc20

In 2019, Wan group described the first Cdc20 degrader **165** (**CP5V**, Fig. 42).^288^ The degrader **165** (**CP5V**) induced the degradation of Cdc20 resulted in significant inhibition of breast cancer cell proliferation and resensitization of paclitaxel-resistant cell lines. The degrader **165** (**CP5V**) played an important role in inhibiting the progression of breast tumors. In cultured cells and preclinical breast cancer models, it induced the degradation of Cdc20 to induce mitosis inhibition, thereby inhibiting cancer cell proliferation. As a result, it played an important role in inhibiting breast cancer tumor progression in vivo. **CP5V**-induced degradation of Cdc20 may be an effective treatment strategy for breast cancer anti-mitotic therapy.

#### CDK2

Inactivation of cyclin-dependent kinase 2(CDK2), which overcomes the differentiation arrest of acute myeloid leukemia(AML) cells, maybe a promising approach for the treatment of AML.^289–291^ However, there are no available selective CDK2 inhibitors.^292,293^

**Fig. 43:**
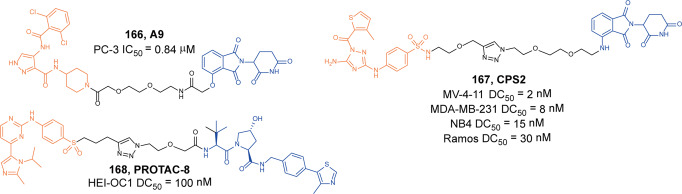
The representative PROTACs targeting CDK2

In 2020, Chen group used **AT-7519** as the CDK2 targeting ligand connected to CRBN ligand through different linkers to obtain a series of degraders.^294^ They found the degrader **166** (**9A**, Fig. 43) had a good selective degradation of CDK2 through activity screening. And it had no effect on CDK5 and CDK9. Subsequently, the degrader **166** (**9A**) showed good inhibitory activity against cell proliferation with IC_50_ of 0.84 µM in PC-3 cells.

In 2021, Rao group developed a first-in-class CDK2 degrader **167** (**CPS2**, Fig. 43) by conjugating a CRBN ligand and a nonselective CDK2 ligand **JNJ-7706621**.^295^ The degrader **167** (**CPS2**) induced the rapid and potent degradation of CDK2 in different cell lines without comparable degradation of other targets, and induced remarkable differentiation of AML cell lines and primary patient cells. These data clearly demonstrated the practicality and importance of PROTACs as alternative tools for verifying CDK2 protein functions.

Also in the same year, Zuo group reported the another CDK2 degrader **168** (**PROTAC-8**, Fig. 43) based on **AZD5438** and **VH032.**^296^ They found the degrader **168** (**PROTAC-8**) could selectively induce the degradation of CDK2 protein. The DC_50_ was about 100 nM to CDK2 while no degradation activity for other subtypes of CDK protein. They also found that it could be used to prevent and treat cisplatin ototoxicity and excitotoxicity of kainic acid in zebrafish. It the first report that CDK2 degrader could be used to prevent and treat acquired hearing loss.

#### CDK2/5

**Fig. 44:**
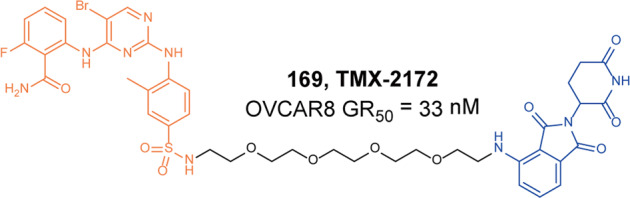
The representative PROTAC targeting CDK2/5

In 2020, Gray group modified the structure of a pan-CDK inhibitor **TMX-2039** to obtain a compound **TMX-3010** that increased the selectivity for CDK1/2/5, but still had a certain constant activity on CDK4/6 (IC_50_ ≈ 100 nM).^297^ **TMX-3010** improved the selectivity to CDK1/2/5, possibly because the linker moiety significantly impaired the inhibitory activity on CDK4 and CDK6. Based on **TMX-3010**, they developed a selective degrader **169** (**TMX-2172**, Fig. 44) for CDK2 and CDK5 over other CDKs. In OVCAR8 cells, the degrader **169** (**TMX-2172**) selectively induced the degradation of CDK2 and CDK5 in a time and dose-dependent manner, while having no influence on other CDKs. Although CDK5 deletion did not cause growth defects in OVCAR8 cells, a more selective CDK2 degrader was in urgent need of development.

#### CDK2/4/6

CDK4 and CDK6 are a family of serine-threonine kinases that interact with cyclins to phosphorylate Rb, thereby regulating the G1/S transition of the cell cycle. Phosphorylation of Rb leads to the release of the transcription factor E2F to activate many transcription genes that are responsible for cell cycle progression.^298^ Among CDKs, CDK4/6 play an important role in the cell cycle and are often overexpressed or overactivated in tumor samples.^299^ However, the current clinical use of CDK4/6 inhibitors have limited its application due to its drug resistance and off-target effects.^300^ So far, many selective and nonselective degraders of CDK4/6 have been reported.^301–306^ Here, we make a supplement to the CDK4/6 degraders reported in recent 2 years.

**Fig. 45:**
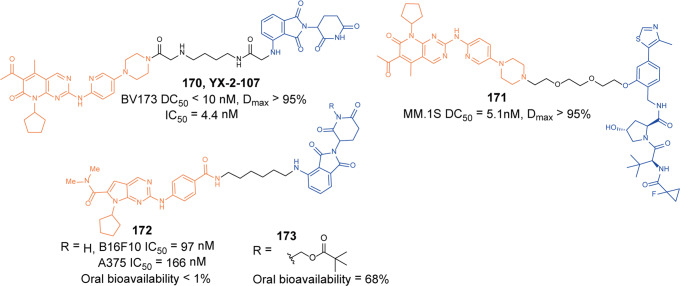
The representative PROTACs targeting CDK2/4/6

In 2021, Dominici group reported their work on the development of selective CDK6 degraders.^307^ The results showed that the degradation of CDK6 was more effective than inhibition with the dual CDK4/6 inhibitor **Palbociclib** in suppressing Ph-positive ALL in mice, suggesting that the growth-promoting effects were CDK6 kinase-independent in Ph-positive ALL. The most effective degrader **170** (**YX-2-107**, Fig. 45) induced the rapid and selective degradation of CDK6 over CDK4 in Ph-positive ALL cells, and significantly inhibited S-phase cells and inhibited the expression of phosphor-RB and FoxM1 regulated by CDK6. The degrader **170** (**YX-2-107**) showed good antitumor effects in Ph-positive ALL xenografts and it had a good safety window. However, it was fast metabolized in mice with a t_1/2_ of 1 h, suggesting that further improvement in PK parameters was warranted.

In addition to CRBN and MDM2 ligands, the VHL and cIPA ligands have also successfully been utilized in PROTAC for the degradation of CDK4/6. In 2020, Kronke group designed and generated series of VHL and cIPA-based CDK4/6 degraders.^308^ The VHL-based degraders were either specific degradation for CDK6 or exhibited dual degradation for CDK4 and CDK6. The most representative degrader **171** (Fig. 45) was a selective CDK6 degrader with the DC_50_ value of 5.1 nM and *D*_max_ more than 95% in MM.1S cells. However, cIAP-based degraders induced a combined degradation of CDK4/6 and IAPs resulted in synergistic effects on cancer cell growth. In conclusion, the results showed that VHL and cIAP-based degraders were an attractive approach for targeted degradation of CDK4/6 in cancer.

A growing number of reports indicated that inhibitors targeting CDK2/4/6 could act as a more feasible chemotherapy strategy. In 2021, Wei group developed a degrader **172** (Fig. 45) based on **Ribociclib** derivative. The degrader **172** could selectively induce the degradation of CDK2/4/6 over other CDKs.^309^ It also showed reduced protein levels of CDK2/4/6 in a dose and time-dependent manner as well as inhibition of the retinoblastoma (Rb) protein phosphorylation in malignant melanoma cells. Moreover, it also remarkably induced cell cycle arrest and apoptosis of melanoma cells. However, the degrader **172** showed poor oral bioavailability. By adding a labile group to the degrader **172** they got the new degrader **173** (Fig. 45), whose oral bioavailability significantly increased up to 68%. In B16F10 xenograft model, oral administration (200 mg/kg) of the degrader **173** showed a significant reduction in tumor growth. This may also provide a universal solution for oral CRBN-based degraders.

#### CDK9

Cyclin-dependent kinases (CDKs) are a class of Ser/Thr protein kinases that have key roles in cell cycle regulation or cell transcription.^310^ CDK9 is a member of the family of CDKs and is crucial in transcription regulation that regulates most of the cancer suppressors and oncogenes.^311^ It overexpressed in a variety of tumors such as leukemia and malignant melanoma. CDK9 inhibitors can inhibit the kinase activity and phosphorylation of CDK9, thereby preventing P-TEFb-mediated activation of RNA Pol II, and inhibiting gene transcription of many anti-apoptotic proteins.^312^ Although a large amount of data indicates that CDK9 is a promising target for cancer treatment, the development of highly selective CDK9 inhibitors is still a huge challenge. Indiscriminate inhibition of CDK family kinase activity can cause adverse side effects and undesirable toxicity.^313^ In recent years, some examples of selective CDK9 degrader have been reported.

**Fig. 46:**
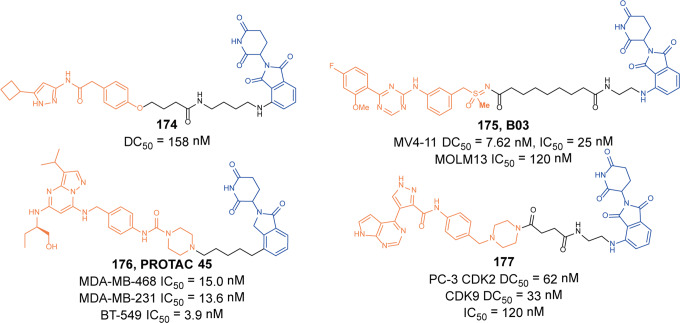
The representative PROTACs targeting CDK9

In 2017, Rana group reported the first CDK9 degrader by conjugating a CDK9 inhibitor **Nopyrazole** and thalidomide. The degrader could potently induce 56% CDK9 to degrade at 10 μM, which was not enough for tumor treatment.^314^ Recently, Natarajan group reported a similar degrader **174** (Fig. 46) with better degradation activity by changing the length of linker. The degrader **174** could selectively induce degradation of CDK9 with DC_50_ of 158 nM while sparing other CDK family members.^315^ These results indicated that the length of the linker was important for degradation activity and selectivity of CDK9.

In 2021, Bian group reported a selective degrader **175** (**B03**, Fig. 46) for CDK9 by conjugating a selective CDK9 inhibitor **BAY-1143572** and pomalidomide.^316^ The degrader **175** (**B03**) induced the degradation of CDK9 in acute myeloid leukemia cells with DC_50_ of 7.6 nM, which was superior to the reported CDK9 degraders. In addition, the degrader **175** (**B03**) strongly induced apoptosis and inhibited cell proliferation of MV4-11 cells (IC_50_ = 25 nM), which was better than the inhibitor **BAY-1143572** (IC_50_ = 560 nM). Moreover, the degrader **175** (**B03**) could induce the degradation of CDK9 in vivo. It was the lead degrader of further development and CDK9 degradation was a potentially valuable treatment strategy for acute myeloid leukemia.

Based on the previous results and through the analysis of the CDK9 ligands binding modes, Chen group also designed and developed a series of new CDK9 protein degraders in the same year.^294^ They found the degrader **176** (**PROTAC 45**, Fig. 46) had better activity in inhibiting the growth of TNBC cells, the DC_50_ reached up to 4 nM in BT-549 cells. And the degrader **176** (**PROTAC 45**) could effectively and selectively induce degradation of CDK9 in a short period of time. In addition, mechanistic studies have shown that the degrader **176** (**PROTAC 45**) could effectively downregulate the downstream targets of CDK9 (such as MCL1 and MYC) at the transcriptional level. Pharmacodynamic studies have shown that the degrader **176** (**PROTAC 45**) could effectively induce tumor regression in a TNBC xenograft model. This was the first reported in vivo evaluation of the therapeutic activity of CDK9 degrader in the treatment of TNBC, which proved that CDK9 degradation therapy was a new method for the treatment of TNBC.

In 2020, Chen group first developed a series of novel degraders for CDK2/9 degradation by connecting CDK9 inhibitor **FN-1501** and CRBN ligand.^317^ The representative degrader **177** (Fig. 46) potently induced degradation of both CDK2 (DC_50_ = 62 nM) and CDK9 (DC_50_ = 33 nM) in PC-3 cells, and inhibited cell proliferation by effectively blocking the cell cycle in S and G2/M phases. The degradation of CDK2/9 by dual PROTAC may be a potentially effective therapeutic approach.

#### CDK12

CDK12 can phosphorylate RNA polymerase II to regulate transcription elongation, and it can also play a key role in RNA splicing, DNA-damage response (DDR) and the maintenance of genome stability.^318,319^ It is overexpressed in breast cancer, ovarian cancer, prostate cancer and other cancers, and is a valuable target for cancer valuable.^318^ Loss of CDK12 function by silencing and using selective covalent CDK12/13 inhibitor **THZ531** leads to a decrease in DDR-associated genes transcription and increases sensitivity to PARP inhibitors and platinum chemotherapy. Thus, CDK12 inhibitors can be used in combination with DNA damaging agents for HR-deficient cancers.^320,321^ However, the development of CDK12 inhibitors is particularly challenging due to high sequence similarity with its close homolog CDK13.^322^

**Fig. 47:**
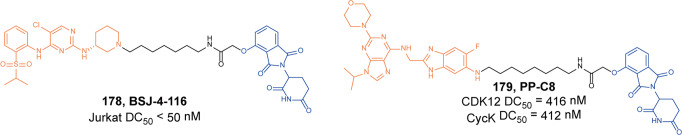
The representative PROTACs targeting CDK12

In 2021, Gray group developed a selective CDK12 degrader **178** (**BSJ-4-116**, Fig. 47) by linking piperidine moiety of optimized segment to thalidomide.^323^ As expected, the degrader **178** (**BSJ-4-116**) demonstrated strong inhibitory activity on CDK12 enzyme activity and effectively induced the degradation of CDK12 in a dose and time-dependent manner in Jurkat cells, while CDK13 protein levels were minimally affected. Moreover, it exhibited potent antiproliferative effects when used alone or in combination with the PARP **Olaparib** as well as used alone against cell lines that resistant to covalent CDK12 inhibitors. Interestingly, chronic exposure of degrader **178** (**BSJ-4-116**) to MOLT-4 cells and Jurkat cells resulted in acquired resistance phenotypes via mutations of Ile733Val and Gly739Ser on CDK12 which resulted in the weaken of degrader binding affinity.

Then Zhu group synthesized a potent PROTAC **179** (**PP-C8**, Fig. 47) based on CDK12/13 noncovalent dual inhibitor **SR-4835**.^324^ They demonstrated that the degrader **179** (**PP-C8**) could selectively induce the degradation of CDK12, while there is no degradation activity on CDK13 in different cell lines. And they also found that the degrader could lead to the degradation of cyclin K, and the degradation activity DC_50_ for CDK12 and cyclin K were 416 and 412 nM, respectively. They also proved the degrader **179** (**PP-C8**) was highly selective to CDK12-cyclin K complex by quantitative proteomics. They have also shown that **179** (**PP-C8**) and PARP inhibitors exhibited good synergy in triple-negative breast cancer (TNBC).

#### Wee1

Wee1 is a tyrosine kinase that regulates the G2/M cell cycle checkpoint by phosphorylating and inactivating CDK1 in response to extrinsic DNA damage and errors in DNA synthesis, thereby preventing mitotic entry.^325^ Cancer cells often have a deficient G1/S checkpoint which frequently via mutation of p53 to leave them reliant on the G2/M checkpoint to avoid mitotic catastrophe. Therefore, abrogation of the G2/M checkpoint by inhibiting Wee1 can sensitize tumors to DNA damaging therapies. In previous studies, Wee1 inhibitor **AZD1775** has been discovered.^326^ However, it has obvious off-target and side effects, which limits its clinical application.

**Fig. 48:**
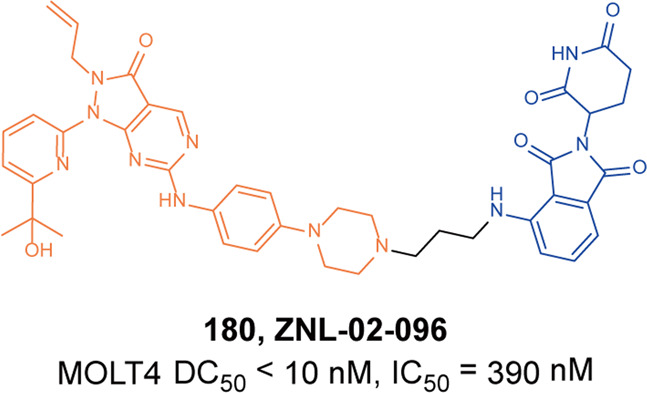
The representative PROTAC targeting Wee1

In 2019, in an attempt to overcome these limitations, Gray group developed Wee1 degraders by conjugating **AZD1775** to pomalidomide.^327^ The degrader **180** (**ZNL-02-096**, Fig. 48) displayed potent Wee1 degradation in MOLT-4 cells while having no influence on PLK1. In addition, it induced G2/M phase arrest at 10 folds lower doses than **AZD1775** and synergized with **Olaparib** in ovarian cancer cells.

### UPS-related protein

#### CRBN

The cereblon (CRBN) is a subunit of the E3 ubiquitin ligase complex cullin-RING ligase 4 (CRL4^CRBN^) and is the substrate receptor for the damaged DNA binding protein 1 (DDB1) and Cul4A.^328,329^ CRBN is also the direct target protein of thalidomide and immunomodulatory imide drugs (IMiDs) and is crucial for thalidomide teratogenicity.^330^ CRL4^CRBN^ is a unique E3 ubiquitin ligase with substrate selectivity altered by ligands such as IMiDs.^331^ It can be efficiently recruited to a protein of interest (POI) when binding with IMiD-based proteolysis-targeting chimeras(PROTACs), leading to the protein ubiquitination and proteasomal degradation.^332^ As the primary IMiD target, CRBN is a protein of huge potential practice.^333^ CRBN degraders are considered to be useful tools to figure out the molecular mechanism of thalidomide analogs^334^.

**Fig. 49:**
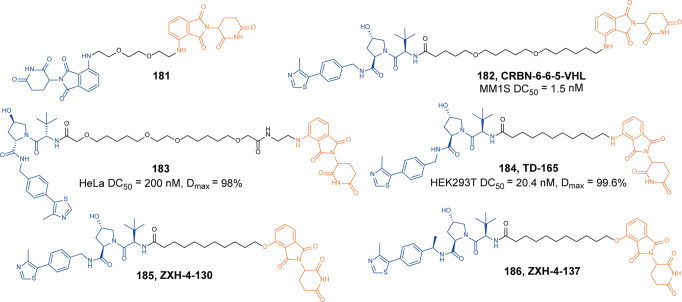
The representative PROTACs targeting CRBN

In 2018, Gütschow group designed a series of PROTACs that targeted CRBN for the first time, proving the feasibility of a degrader-induced degradation of CRBN.^330^ They synthesized so-called homo-ROTACs by linking the two pomalidomide molecules. They found the degrader **181** (Fig. 49) could selectively induce the degradation of CRBN with minimal effects to other related proteins such as IKZF1, IKZF3 and CRL4. The maximum degradation activity of CRBN was achieved at 6h after treatment with 100 nM degrader **181**. The homo-PROTACs could overcome the low selectivity of traditional chemical probes and also provide a new tool for exploring the mechanism of CRBN and IMiDs. Subsequently, they also reported several hetero-PROTACs by assembling CRBN and VHL in 2019. One of the particularly potent CRBN degrader **182** (**CRBN-6-5-5-VHL**, Fig. 49) had the best degradation activity of CRBN with DC_50_ of 1.5 nM in MM1S cells.^334^ Compared with the degrader **181**, hetero-PROTACs such as **CRBN-6-5-5-VHL**, **CRBN-6-6-6-VHL** and **CRBN-4-4-4-6-VHL** all could achieve more efficient degradation activities of CRBN while inducing almost negligible degradation of the neo-substrates IKZF1 and IKZF3.

In 2019, Ciulli group reported their most active VHL-CRBN hetero-dimerizing degrader **183** (Fig. 49) which induced the rapid and efficient degradation of CRBN with a DC_50_ of 200 nM in HeLa cells (within 1 h of treatment). Their work provided proof-of-concept for hijacking different E3 ligase to induce degradation of a particular one.^335^

Subsequently, Kim group developed a series of VHL-CRBN hetero-dimerizing degraders which effectively induced degradation of CRBN but not VHL.^336^ To develop VHL-CRBN hetero-dimerizing PROTACs they designed different linkers to connect pomalidomide with **VH032**. The linkers were designed to connect the amino group of pomalidomide and the terminal acetyl group of **VH032**. The most potent and selective degrader **184** (**TD-165**, Fig. 49) could induce the degradation of CRBN with DC_50_ of 20.4 nM in HEK293T cells.

Recently, Gray group developed six homo-PROTACs and six hetero-PROTACs adopting a similar strategy as the previous ones. By combining protein immunology and genomics analysis, they successfully screened out two effective hetero-PROTACs which could selectively induce the degradation of CRBN, which provids a reference for follow-up research. The degradation efficiency of degrader **185** (**ZXH-4-130**, Fig. 49) and degrader **186** (**ZXH-4-137**, Fig. 49) were better than degrader **183** and degrader **184** (**TD-165**).^337^

#### hRpn13^*Pru*^

The 26S proteasome consists of a 20S core and a 19S regulatory subunit. hRpn13 is a component of the 19S subunit, which contains two functional domains, an N-terminal pleckstrin-like receptor for ubiquitin(Pru) domain and a C-terminal deubiquitinase adaptor (DEUBAD) domain.^338–340^

**Fig. 50:**
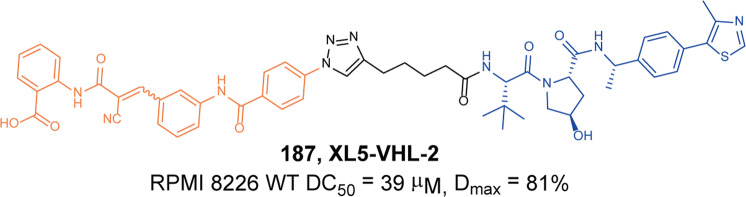
The representative PROTAC targeting hRpn13^*Pru*^

In 2021, Walters group screened an inhibitor **XL5** with better binding ability based on the reported structure of hRpn13 inhibitor **RA190.**^341^ On the basis of XL15, they used different E3 ligase ligands to connect with it to obtain a series of PROTAC molecules. They found that **187** (**XL5-VHL-2**, Fig. 50) was able to induce the degradation of DEUBAD-lacking hRpn13^*Pru*^ species in RPMI-8226 wild-type cells, but not hRpn13. The degradation activity of DEUBAD-lacking hRpn13^*Pru*^ species was DC_50_ of 39 µM and *D*_max_ of 81%. When treated with **187** (**XL5-VHL-2**), it only resulted in ubiquitination of hRpn13 protein without degradation.

#### VHL

E3 ubiquitin ligases are emerging as attractive targets for small-molecule modulation and drug discovery and gaining importance as targets to small molecules, both for direct inhibition and to be hijacked to induce the degradation of non-native neo-substrates using bivalent compounds known as PROTACs.^342^ However, E3 ligases do not comprise deep and “druggable” active sites for binding to small molecules. E3 ligase inhibition may be ineffective or fail to recapitulate genetic knockout or knockdown.^343^ New chemical modalities to target E3 ligases are therefore demanded.

**Fig. 51:**
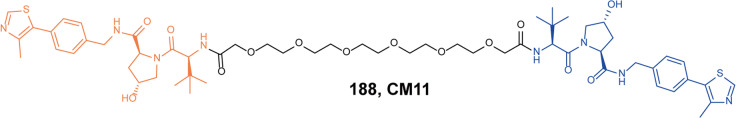
The representative PROTAC targeting VHL

In 2017, Ciulli group described proof-of-concept of homo-PROTACs using diverse molecules composed of two instances of a ligand for the VHL E3 ligase.^344^ The most active degrader **188** (**CM11**, Fig. 51) dimerized VHL with high avidity in vitro and induced potent, rapid, and proteasome-dependent self-degradation of VHL in different cell lines, in a highly isoform-selective fashion and without triggering a hypoxic response. As a novel chemical probe for selective VHL degradation, the degrader **188** (**CM11**) would find wide use in investigating and dissecting the biological functions of pVHL.

#### ZFP91

ZFP91 is a newly discovered ubiquitin E3 ligase that is upregulated in prostate cancer, acute myeloid leukemia, and colon cancer tumors. It maintains the stability of NIK protein through K63-ubiquitinated NIK, thereby activating the NF-κB signaling pathway and promoting the occurrence and development of tumors.^345^

**Fig. 52:**
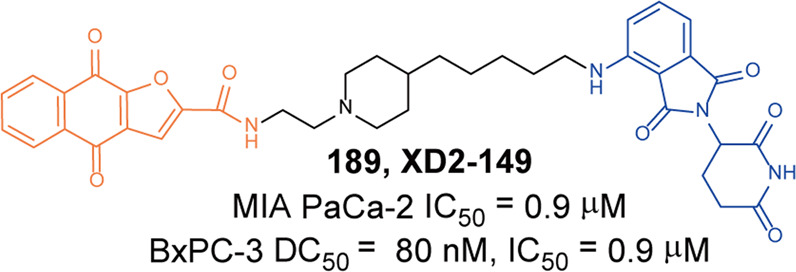
The representative PROTAC targeting ZFP91

In 2021, Neamati group designed a ZFP91 degrader **189** (**XD2-149**, Fig. 52) based on **Napabucasin**, which was undergoing multiple clinical trials and was reported to inhibit STAT3, and the degrader **189** (**XD2-149**) resulted in inhibition of STAT3 signaling in pancreatic cancer cell lines without inducing proteasome-dependent degradation of STAT3.^346^ Proteomics analysis showed that it could induce the degradation of the E3 ubiquitinprotein ligase ZFP91 with DC_50_ values of 80 nM in BxPC-3 cells. At the same time, it also showed a moderate antiproliferative activity, and the IC_50_ was about 0.9 µM in MIA PaCa-2 cells and BxPC-3 cells.

### Other proteins

#### BTK

As a key regulator in the B-cell receptor(BCR) signaling pathway, BTK is a non-receptor cytoplasmic tyrosine kinase and plays a key role in B-cell lymphomas.^347^ In 2013, although the covalent inhibitor ibrutinib was approved for treating MCL and activated B-cell-like (ABC)-DLBCL by FDA, the patients developed resistance due to the missense BTK mutation of C481S.^348^ With the advent of PROTACs technology, there have been many noncovalent degraders which can degrade both the wild-type and ibrutinib-resistant C481S BTK effectively.^349–353^ The next stage may be aimed at improving druggability for a clinical study.

**Fig. 53:**
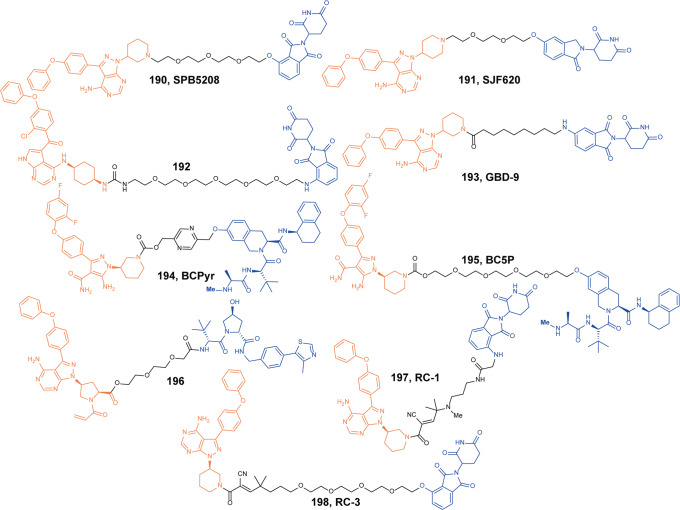
The representative PROTACs targeting BTK

In 2019, Lin group developed a new BTK degrader **190** (**SPB5208**, Fig. 53) by conjugating **Ibrutinib** and thalidomide.^354^ The degrader **190** (**SPB5208**) could inhibit the growth of the JeKo-1 cells and induce the degradation of BTK protein both in vitro and in vivo.

In 2020, Crews group illustrated a new series of BTK degraders based on **MT802** which was reported previously with potent degradation activity of BTK and poor pharmacokinetic properties. To develop an appropriate degrader for in vivo study, they modified the linker and E3 ligands while keeping their length constant. Fortunately, the degrader **191** (**SJF620**, Fig. 53) exhibited both potent degradation activity and encouraging pharmacokinetic profile.^355^

In 2021, Wang group designed a novel series of PROTACs targeting BTK based on **ARQ531**, a reversible noncovalent BTK inhibitor that inhibits both wild-type (WT) and mutated BTK. In addition to having moderate membrane permeability and good plasma stability, the most potent degrader **192** (Fig. 53) also could induce the degradation of BTK^*WT*^/BTK^*C481S*^ and inhibit BTK^*WT*^/BTK^*C481S*^ TMD8 cells growth effectively.^356^

In 2021, Rao group developed a new type degrader of targeted protein degradation by merging PROTAC and molecular glue for inducing the degradation of BTK and GSPT1 proteins concurrently. They designed a BTK PROTACs library with short linkers to keep the balance of the efficiency of PROTACs and the features of molecular glue.^357^ The representative degrader **193** (**GBD-9**, Fig. 53) induced the fast and effective degradation of BTK and GSPT1 in DOHH2 cells and exhibited more potent antiproliferative activity than **Ibrutinib** on a variety of DLBCL and AML cell lines. This work not only provided a new example for the design of double-mechanism degraders with the characteristics of molecular glue and PROTACs, but also expanded the indications of PROTACs targeting BTK, which may have broader clinical application prospects.

In 2021, Pfizer solved the unique BTK-degrader-cIAP1 ternary complex crystal structure by using the degrader **195** (**BC5P**, Fig. 53) which was derived from aminopyrazole series in previous work and it was shown that BTK was flexibly tethered to cIAP1 with a multitude of sampled conformations. To restrict this flexibility, a shorter and more rigid linker was induced to obtain the degrader **194** (**BCPyr**, Fig. 53) which enhanced protein–protein interactions, restricted conformational dynamics but weakened BTK- degradation activities. This indicated that the cooperativity of ternary complex did not necessarily correlate with degradation activity.^358^

Although **Ibrutinib** is an irreversible covalent inhibitor, most PROTACs targeting BTK are based on noncovalent binding. Certainly, there have been many attempts to design covalent PROTACs in recent years. In 2020, Pan group illustrated a new class of BTK PROTACs based on an irreversible covalent binder. Although the degrader **196** (Fig. 53) could induce the degradation of BTK protein with a DC_50_ of 136 nM and a *D*_max_ of 88%, its potency was reduced due to the loss of substoichiometric activity.^359^ In 2020, Nir group^360^ and Wang group^361^ successively reported reversible covalent BTK PROTACs which enhanced the binding affinity of inhibitor without compromising the substoichiometric activity of PROTACs. Coincidentally, they both developed reversible covalent PROTACs based on cyanoacrylamide. Interestingly, in Wang work, the reversible covalent degrader **197** (**RC-1**, Fig. 53) had high target occupancy and worked as both an inhibitor and a degrader which exhibited more potent degradation activity of BTK^*WT*^ and BTK^*C481S*^ than the equivalent irreversible covalent and reversible noncovalent degrader. Conversely, the degrader **198** (**RC-3**, Fig. 53) was the best reversible covalent BTK PROTACs in Nir work, even less potent than the equivalent irreversible covalent and reversible noncovalent degrader. But Nir work mainly illustrated the molecular mechanism that part of the degradation by irreversible covalent PROTACs was driven by reversible binding prior to covalent bond formation, while the reversible covalent PROTACs drave degradation primarily by covalent engagement.

Finally, the authors compared the reported BTK degraders (Table 6). It was found that although there were various types of BTK warheads currently used, **Ibrutinib** was used more frequently and others were used less. In the selection of E3 ligases, most of the currently used was CRBN, and VHL and cIAP have been mentioned in some reports. Therefore, more BTK degraders based on different BTK inhibitors and E3 ligases need to be developed.

**Table 6:** The summary and comparison of PROTACs targeting BTK

No.	PROTAC	Warhead	E3 ligase
1	**SPB5208(190)**	**Ibrutinib**	CRBN
2	**SJF620(191)**	**Ibrutinib**	CRBN
3	**192**	**ARQ531**	CRBN
4	**GBD-9(193)**	**Ibrutinib**	CRBN
5	**BCPyr(194)**	Aminopyrazole series	cIAP
6	**BC5P(195)**	Aminopyrazole series	cIAP
7	**196**	**Ibrutinib**	VHL
8	**RC-1(197)**	**Ibrutinib**	CRBN
9	**RC-3(198)**	**Ibrutinib**	CRBN

#### CCR9

CCR9 is a chemokine receptor expressed on memory/effector CD4+ T cells and selectively binds the chemokine CCL25.^362^ The expression of CCL25 is significantly increased when the intestine is inflamed and drives lymphocyte migration to the intestinal tissue through interaction with CCR9.

It a treatment for Crohn disease to prevent the migration of lymphocytes to the intestinal tissue by inhibiting the interaction of CCR9 and CCL25. **Vercirnon** is a small-molecule CCR9-selective antagonist that binds to the CCR9 receptor on the intracellular side of the GPCR, but not on the extracellular side of the cell where GPCR ligands normally bind.^363^ By binding to this allosteric site on the CCR9 receptor, **Vercirnon** prevents CCR9 from interacting with intracellular signaling molecules, thereby achieving an antagonistic effect.

**Fig. 54:**
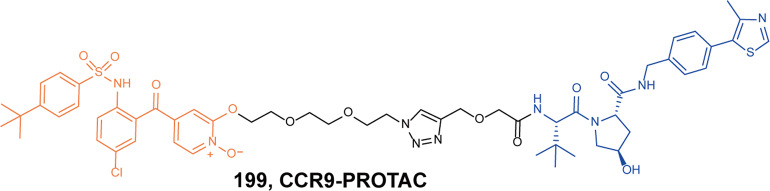
The representative PROTAC targeting CCR9

Based on the binding mode of **Vercirnon** and CCR9, Schiedel group obtained a more active antagonist.^364^ Based on the structure of the new antagonist, they designed a CCR9-targeted degrader **199** (**CCR9-PROTAC**, Fig. 54) and then they tested the degradation activity of the degrader on CCR9 in HEK293T cells. They found the degrader **199** (**CCR9-PROTAC**) could induce the degradation of CCR9 at 1 nM, but the “hook effect” also appeared as the concentration increased. This was the first PROTAC targeted the intracellular allosteric binding site of GPCRs and provided an unprecedented approach to modulate GPCR activity.

#### CD147

CD147 is a transmembrane glycoprotein, a member of the immunoglobulin superfamily. As a tumor-associated antigen, it is highly expressed in various tumors, including malignant melanoma (MM). As a pivotal role in tumor progression, including affecting tumor cell apoptosis, invasion and metastasis. Therefore, CD147 may provide a potential drug target for tumor diagnosis and treatment.^365^

**Fig. 55:**
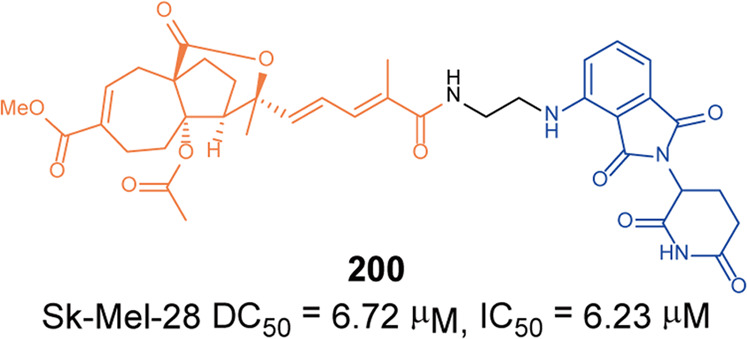
The representative PROTAC targeting CD147

To date, the anti-CD147 monoclonal antibody drug **Licartin** has been used in clinical treatment of liver cancer, but the radioactive **I**^130^ contained in **Licartin** may have inconvenience and risk of radiation leakage in clinical application.^366^ Pseudolaric acid B (**PAB**), a recently reported natural product, was an antagonist of CD147, which could avoid the above-mentioned problems.^367^ So in 2020, Chen group reported the first CRBN-based CD147 degrader **200** (Fig. 55) derived from **PAB**.^368^ The degrader **200** could effectively induce the degradation of CD147 with DC_50_ of 6.72 µM in Sk-Mel-28 cells and inhibited melanoma cells in vitro and in vivo. In summary, the degrader **200** as the novel type of anticancer agent provided a new method for CD147-mediated cancer treatment.

#### CRABP

Cytoplasmic retinoic acid-binding proteins, CRABP-I and CRABP-II, are found in all vertebrates and are conserved across species. CRABP-I is thought to be related to metabolism of retinoic acid(RA) and resistance to RA in cancer cells, while CRABP-II is suggested to be associated with nuclear transportation of RA.^369^ CRABP-II is expressed in tissues such as the choroid plexus, gut endoderm, and interdigital mesenchyme, which do not express CRABP-I.^370^ CRABP-I/II are related to Alzheimer disease and various cancer.^371,372^ Therefore, CRABPs could be target proteins for treatment of these diseases. However, it is challenging to directly inhibit the functions of CRABPs by small molecules.

**Fig. 56:**
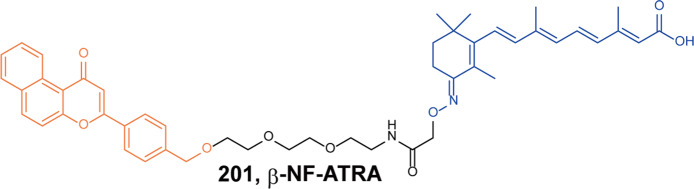
The representative PROTAC targeting CRABP

In 2010, Hashimoto group developed the first cIAP1-based PROTAC by hijacking cIAP1-E3 ligase (bestatinmethyl ester, **MeBS**) and all-trans retinoic acid (**ATRA**) to induce the degradation of CRABP-I/II.^373^ Based on this work Naito group developed new class CRABPs degrader **201** (**β-NF-ATRA**, Fig. 56) that used **β-naphthoflavone** (**β-NF**) as a ligand to replace the **MeBS** to recruit aryl hydrocarbon receptor(AhR) E3 ligase complexes in 2019.^374^ The degrader **201** (**β-NF-ATRA**) could effectively induce the degradation of CRABP-I/II in a dose and time-dependent manner in MCF-7 and IMR-32 cells, and induced AhR self-ubiquitylation and was degraded by the proteasome, which may cause the potential for synergistic anti-tumorigenic activity.

#### HSP90

Heat shock protein 90 (HSP90) is a ubiquitous, highly conserved and highly active protein in cells, which is more abundant in tumor cells than in normal cells. As a molecular chaperone, HSP90 assists different kinds of oncoproteins to fold, stabilize and mature.^375^ The service proteins of HSP90 contain a large number of signal transduction molecules such as kinases and transcription factors, which play an important role in tumor formation and growth.^376^ HSP90 inhibitors are a class of compounds that can bind to HSP90-regulatory sites, cause conformational changes of HSP90 and induce degradation of substrate proteins, thereby exerting an inhibitory effect.^377^ HSP90 inhibitors also can cause multiple signal transduction pathways of tumor cells to be inhibited. Many kinds of HSP90 inhibitors have entered the clinical phase.

**Fig. 57:**
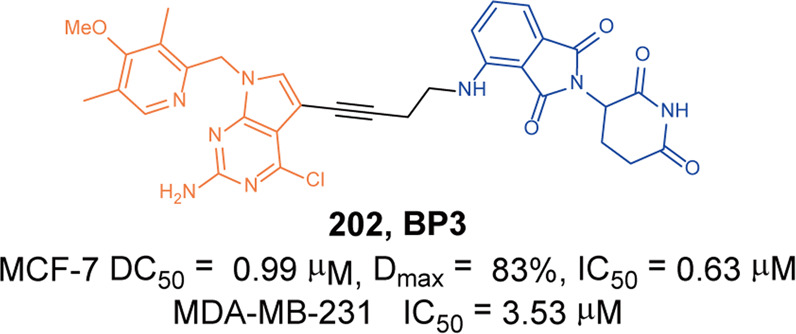
The representative PROTAC targeting HSP90

Wu group designed and synthesized a series of PROTACs based on the binding mode of HSP90 inhibitor **BIIB021** to the protein.^378^ Then they tested their degradation activity on HSP90 protein and proliferation inhibitory activity in MCF-7 and MDA-MB-231 cells, they found the degrader **202** (**BP3**, Fig. 57) could induce the degradation of HSP90 protein with a DC_50_ of 0.99 µM and an IC_50_ of 0.63 µM for cell proliferation inhibitory activity in MCF-7 cells. When used as a single drug, degrader **202** (**BP3**) could effectively inhibit the tumor proliferation in mice.

#### IDO1

Indoleamine 2,3-dioxygenase 1 (IDO1) is a heme enzyme that catalyzes the first and rate-limiting steps of the decomposition of tryptophan into *N*-formyl-aminoylinosine. It acts on a variety of tryptophan substrates, including *D*-tryptophan, *L*-tryptophan, 5-hydroxytryptophan, tryptophan, serotonin, and so on.^379^ IDO1 plays a role in various pathophysiological processes, such as antibacterial, antitumor defense, neuropathology, immunomodulation, and antioxidant activity.^380^ Recently, a large number of reports have confirmed that IDO1 was overexpressed in a variety of cancers and plays an important role in cancer immune escape. Therefore, IDO1 has become one of the important targets in the field of tumor therapy. In fact, several highly effective and selective IDO1 inhibitors have entered the clinical development stage for the treatment of human cancer.^381^

**Fig. 58:**
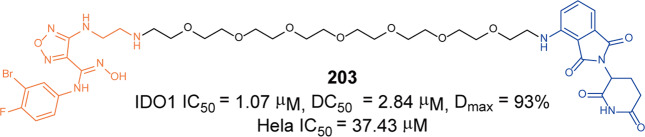
The representative PROTAC targeting IDO1

In 2020, Xie group reported the application of PROTAC technology in the targeted degradation of IDO1 enzymes. They designed a series of targeted IDO1 degraders based on pomalidomide and IDO1 inhibitor **Epacadostat**, and evaluated their degradation activity and anti-proliferation in HeLa cells.^382^ They found that the degrader **203** (Fig. 58) had the best degradation activity of IDO1, its DC_50_ was 2.84 µM, *D*_max_ was 93%. And its antiproliferative activity on HeLa cells was 37.43 µM, which was significantly better than the inhibitor **Epacadostat**.

#### LXR-β

Liver X receptors (LXRs) are members of the orphan nuclear receptor transcription factor superfamily and ligand-dependent transcription factors, including two subtypes of LXR-α (NR1H3) and LXR-β (NR1H2).^383^ LXR-α is mainly expressed in the liver, spleen, kidney, lung, intestine, adipose tissue, and macrophages, and LXR-β is widely and lowly expressed throughout the body.^384^ When LXRs are activated by ligands or synthetic agonists, they can participate in the regulation of sugar, lipid, cholesterol metabolism, immune response, and inflammation to regulate the transcription and expression of target genes.^385^

**Fig. 59:**
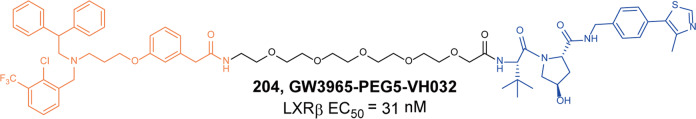
The representative PROTAC targeting LXR-β

In 2021, Demizu group synthesized some LXR-β degraders based on the LXR-α/LXR-β agonist **GW3965** and different E3 ligase ligands through different connection methods and then found that the VH032-based degrader **204** (**GW3965-PEG5-VH032**, Fig. 59) had the best degradation activity of LXR-β, the obvious degradation of LXR-β could be observed when the drug concentration was 3 µM.^386^

#### MIF

Macrophage migration inhibitory factor (MIF) is the first cytokine with the function of multiple inflammatory mediators. It is also an important endocrine hormone.^387^ As an inflammatory chemokine, MIF plays a very important role in many diseases: metabolic diseases (such as atherosclerosis), autoimmune diseases, cancers, infectious diseases(such as sepsis) and wound healing.^388^ Therefore, MIF can be used as a biomarker and target of these diseases.

**Fig. 60:**
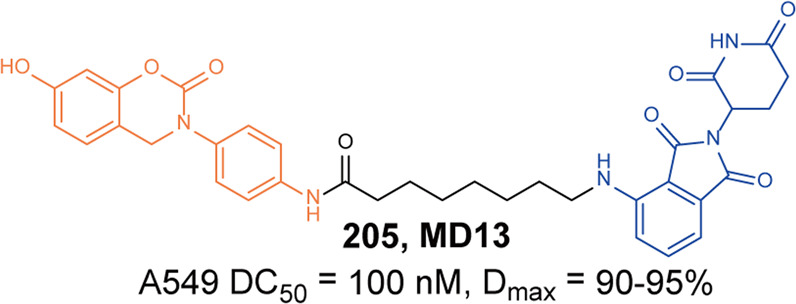
The representative PROTAC targeting MIF

In 2021, Dekker group designed and synthesized a variety of degraders based on the MIF tautomerase inhibitors and pomalidomide.^389^ Among them, the degrader **205** (**MD13**, Fig. 60) could induce the degradation of MIF with a DC_50_ of 100 nM in A549 cells. It induced cell cycle arrest at the G2/M phase and also inhibited ERK phosphorylation.

#### PARP1

The nuclear protein PARP1 has a well-established role in the signaling and repair of DNA, and is a validated therapeutic target for cancers and other human diseases.^390^ Cancer cells bearing mutations in BRCA1 or BRCA 2 are exquisitely sensitive to PARP1 inhibitors, which is called synthetic lethality. To date, several small-molecule PARP1 inhibitors, such as **Olaparib**, **Rucarparib**, **Niraparib**, and **Talazoparib**, have been approved for the treatment of BRCA-mutation ovarian and breast cancers.^391^ However, there are still challenges in terms of PARP1 inhibitors usage that limit their therapeutic utility, including the acquisition of drug resistance, the low proportion of BRCA1 or BRCA 2 mutations in cancer cells, and cytotoxicity caused by PARP1 trapping.^392,393^

**Fig. 61:**
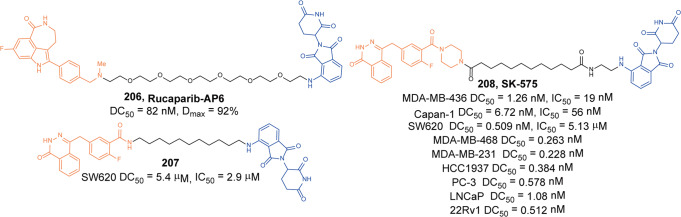
The representative PROTACs targeting PARP1

In 2019, Yu group reported their works on the development of PARP1 degraders.^394^ The degrader **206** (**iRucaparib-AP6**, Fig. 61) induced robust degradation of PARP1 at concentrations as low as 50 nM in primary rat neonatal cardiomyocytes. Knocking down the protein level of PARP1, the degrader **206** (**iRucaparib-AP6**) protected muscle cells and primary cardiomyocytes from DNA-damage-induced energy crisis and cell death. In summary, the degrader **206** (**iRucaparib-AP6**), by blocking both the catalytic and scaffolding functions of PARP1 without causing PARP1 trapping, provided an ideal approach for the treatment of cancers and other diseases caused by PARP1 hyperactivation.

In 2020, Shen group developed several PARP1 degraders based on the combination of PARP1 inhibitor **Olaparib** and lenalidomide.^395^ The representative degrader **207** (Fig. 61) effectively induced the degradation of PARP1 at 10 µM in SW620 cells. At the same time, Chen group reported the design and synthesis of PARP1 degraders based on **Olaparib** and thalidomide**/**lenalidomide.^396^ The representative degrader **208** (**SK-575**, Fig. 61) potently inhibited the growth of cancer cells bearing BRCA1/2 mutations, and induced potent and specific degradation of PARP1 in various human PARP1-positive cancer cells with the *D*_max_ more than 95%. It was worth noting that the PK and PD data showed that a single dose of degrader **208** (**SK-575**) was fully exposed to plasma for more than 24 h and effectively induced PARP1 degradation in SW620 xenograft tumor tissues. Moreover, it exhibited durable tumor growth inhibition in mice when used as a single agent or in combination with cytotoxic agents.

#### PARP14

PARP14 is an interferon-stimulated gene that is overexpressed in a variety of tumors. By regulating IFN-γ and IL-4 signals, affects the polarization of pro-tumor macrophages and inhibits the antitumor inflammatory response. Catalytic inhibitors of PARP14, such as **RBM012042**, can reverse IL-4 driven pro-tumor gene expression in macrophages. However, it is not clear what roles the non-enzymatic biomolecular recognition motifs play in PARP14-driven immunology and inflammation. However, the role of non-enzymatic functions of PARP14 in PARP14-driven immunity and inflammation is still unclear.^397^

**Fig. 62:**
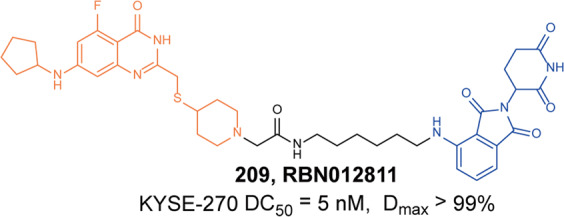
The representative PROTAC targeting PARP14

Kuntz group developed the first-in-class degrader **209** (**RBN012811**, Fig. 62) based on a PARP14 inhibitor **RBN012042.**^398^ The degrader **209** (**RBN012811**) could selectively induce the degradation of endogenous PARP14 with DC_50_ about 5 nM in KYSE-270 cells, and have no effect on the total protein levels of other PARP enzymes. It also could induce a dose-dependent reduction of IL-10 levels in primary human macrophages.

#### PD-L1

Programmed death receptor (PD-1), also known as CD279, is an important immunosuppressive molecule. It regulates the immune system and promotes self-tolerance by downregulating the response of the immune system to human cells and by inhibiting the inflammatory activity of T cells.^399^ PD-L1 is a ligand of PD-1. The combination of PD-1 and PD-L1 initiates the programmed death of T cells and enables tumor cells to obtain immune escape. PD-L1 is upregulated in a variety of tumor cells and it binds to PD-1 on T cells, inhibits T-cell proliferation and activation, inactivates T cells, and finally induces immune escape.^400^

**Fig. 63:**
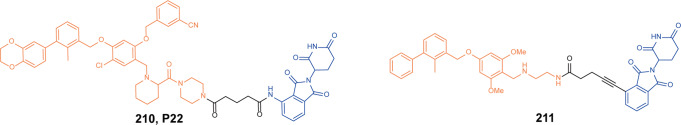
The representative PROTACs targeting PD-L1

In 2020, Chen group first reported a series of novel PD-L1 degraders.^401^ Most of the compounds exhibited excellent inhibitory activities against PD-1/PD-L1 interaction, especially the degrader **210** (**P22**, Fig. 63) with an IC_50_ value of 39.2 nM. The degrader **210** (**P22**) could moderately induce the degradation of PD-L1 in a lysosome-dependent manner, which may contribute to its immune effects. In 2021, Yang group reported another PD-L1 degrader **211** (Fig. 63) based on **BMS-37.**^402^ The degrader **211** could effectively induce the degradation of PD-L1 protein at micromolar in various malignant cells in a proteasome-dependent manner, such as MC-38, MCF-7, and Kasumi-1 cells. Moreover, it could significantly reduce PD-L1 protein levels of MC-38 cancer cells in vivo, and achieve significant tumor growth inhibition in the MC-38 xenograft model. This approach showed that PROTAC could be used as a new alternative strategy for tumor immunotherapy.

#### PLK1

Polo-Like Kinase 1 (PKL1) belongs to the polo-like kinase family, which is a type of serine/threonine kinase that is widely present in eukaryotic cells. The structure of PKL1 is highly conserved, with a kinase domain (KD) at the N-terminus and two conserved polo-box domains (PBD) at the C-terminus.^403^ PBD is related to the subcellular localization and function of PKL1. Normally, the PBD of PLK1 binds to the KD domain to inhibit the phosphorylation of T210 in the KD domain, thereby inhibiting its kinase activity. Once the PBD of PLK1 binds to its ligand, the PBD is immediately separated from the T loop of the kinase domain and PLK1 is activated. PLK1 kinase can interact with a variety of substrates through its kinase activity to regulate cell mitosis, cytokinesis, DNA-damage response, development, and other processes.^404,405^ There is also a destruction box (D box) between the KD and PBD domains, which is closely related to the degradation of PLK1.

**Fig. 64:**
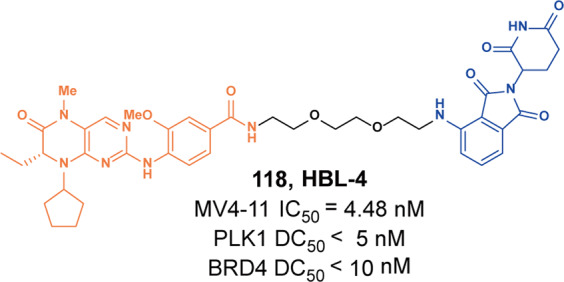
The representative PROTAC targeting PLK1

In 2020, Lu group developed a dual degrader **118** (**HBL-4**, Fig. 64) conjugated with the PLK1 inhibitor **BI2536** and CRBN ligand through a PEG linker.^209^ This was the first degrader for PLK1. The degrader **118** (**HBL-4**) induced the efficient degradation of BRD4 and PLK1 in MV4-11 cells with a DC_50_ of about 10–20 nM and 5 nM, respectively. Then they found the antiproliferative activity of the degrader **118** (**HBL-4**) (IC_50_ = 4.48 nM) was better than **BI2536** (IC_50_ = 88.5 nM) in MV4-11 cells. At the same time, it induced dramatically improved efficacy in the MV4-11 tumor xenograft model compared with **BI2536**.

#### RAR

Retinoic acid receptors (RAR) belong to the nuclear receptor superfamily, including three subtypes of α, β, and γ. RAR-β is divided into β1, β2, β3, β4, and so on. RAR regulates the transcription of target genes by binding to their ligands, thereby exerting various biological effects. It plays an important role in mediating cell growth and apoptosis.^406^

**Fig. 65:**
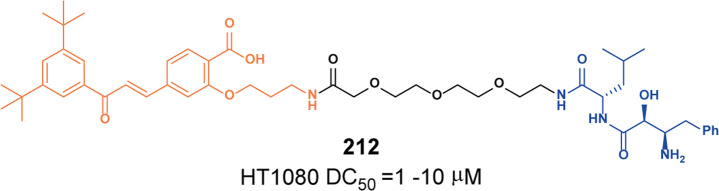
The representative PROTAC targeting RAR

In 2011, Hashimoto group developed an RAR degrader **212** (Fig. 65) conjugated with the RAR inhibitor **Ch55** and cIAP1 ligand **BE04** through a PEG linker.^407^ The degrader **212** could induce the degradation of RAR protein in HT1080 cells for the first time, while had no influence on the amount of CRABP-II.

#### RHAU

Nucleic acids with a four-stranded helix structure are called G-quadruplexes, which are a class of nucleic acid secondary structures with important regulatory functions and are believed to play an important role in gene expression and telomere maintenance.^408^ G-quadruplex binding proteins play an important role in the regulation of life in which G-quadruplexes are involved. G-quadruplex-binding proteins include a variety of proteins, such as BRCA1, PARP1, RHAU, TRF2, and so on.^409^ RNA helicase associated with AU-rich element (RHAU) proteins has been identified as a major source of quadruplex-resolving activity in cell lysates.^410^

**Fig. 66:**
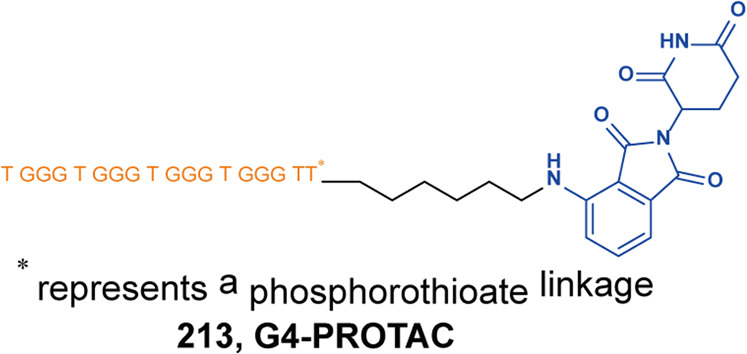
The representative PROTAC targeting RHAU

Phan group reported the novel degrader **199** (**G4-PROTAC**, Fig. 66) generated from a combination of G4 warhead and different E3 ligase ligands.^411^ They found that a significant downregulation of RHAU protein could be observed at **199** (**G4-PROTAC**) concentration of 1 nM, and its degradation activity increased along with increasing degrader concentration. At the same time, the degrader also had obvious “hook effect”. They also proved that the degradation of RHAU protein could be rapidly induced after using the degrader for 6 h, and the degradation effect gradually increased with the prolongation of the incubation time.

#### RIPK2

Receptor serine/threonine kinases are single-pass transmembrane protein receptors with serine/threonine protein kinase activity in the intracellular region. It mainly phosphorylates the serine or threonine in downstream signal proteins, transmits the extracellular signal into the cell, and then achieves a variety of biological functions by affecting gene transcription. Pattern recognition receptors NOD1 and NOD2 are located upstream of RIPK2 and regulate its activity.^412^ The transcriptional activation of a variety of inflammatory cytokine genes and a variety of immune diseases are related to the activation of this pathway.^413^

**Fig. 67:**
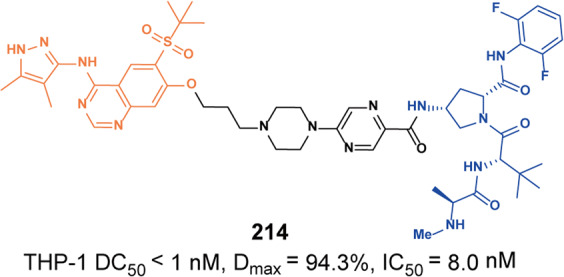
The representative PROTAC targeting RIPK2

In 2021, Harling group developed a highly potent and selective RIPK2 degrader **214** (Fig. 67) based on a novel IAP ligase ligand.^414^ The degrader **214** successfully induced the efficient degradation of RIPK2 in the absence of cIAP1 autoubiquitination over a range of concentrations in human PBMCs. The DC_50_ and *D*_max_ were about 1 nM and 94.3%, respectively. The antiproliferative activity of the degrader **214** (IC_50_ = 10 nM) was better than other RIPK2 degraders. It exhibited low total systemic clearance in both rat and dog which afforded prolonged in vivo degradation of RIPK2 in vivo. In addition, it reduced endogenous RIPK2 in rats at low doses and extended PD that persists in the absence of detectable compound.

#### SF3B1

The spliceosome is a large protein-RNA complex that consists of five small nuclear RNAs (snRNAs) and a variety of associated proteins, such as splicing factor 3B (SF3B).^415^ SF3B subunit 1 (SF3B1) is one of the fundamental components of the multiprotein complex SF3B and is involved in 30 splice site recognition at intron–exon junctions during RNA splicing. The SF3B1 gene is frequently mutated in tumors, such as myelodysplastic syndrome (20%) and chronic lymphocytic leukemia (15%). Because the functions of individual components of the spliceosome are largely unknown, SF3B1 inhibitors (such as **pladienolide B**, **spliceostatin A**, and **herboxidiene**) are widely used to repress spliceosome-mediated alternative splicing and induce apoptosis in various tumor cells.^416^

**Fig. 68:**
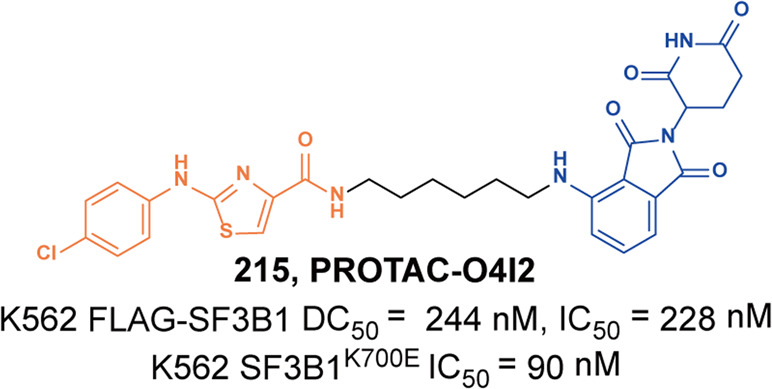
The representative PROTAC targeting SF3B1

In 2021, Cheng group reported the SF3B1 degrader **215** (**PROTAC-O4I2**, Fig. 68) by fusing thalidomide to **O4I.**^417^ They found the degrader **215** (**PROTAC-O4I2**) selectively induced the degradation of SF3B1 and induced cellular apoptosis in a CRBN-dependent manner. In a Drosophila intestinal tumor model, the degrader **215** (**PROTAC-O4I2**) increased survival by interference with the maintenance and proliferation of stem cells. Thus, their results demonstrated that SF3B1 could be degraded by utilizing noninhibitory chemicals, which expanded the PROTAC target proteins.

#### SLC

Among the membrane proteins that constitute about 27% of all proteins in the human genome, the solute carrier(SLC) family is the second largest membrane protein family in humans, and consists of more than 400 types of proteins divided into 52 families. In recent years, phylogenetic analysis has shown that 15 SLC families can be divided into four phylogenetic clusters, namely α, β, γ, and δ. The sequence analysis has shown that 24 SLC families belong to three PFAM clans, MFS, APC, and CPA/AT.^418^ SLCs are responsible for the absorption and transportation of several substances on cell membranes, including amino acids, nucleotides, sugars, inorganic ions, and drugs. Nearly 100 human SLCs that transport amino acids have been proposed, 60% of them have been confirmed to transport amino acids, and the rest are closely related to amino acid transporters known phylogenetically.^419^

**Fig. 69:**
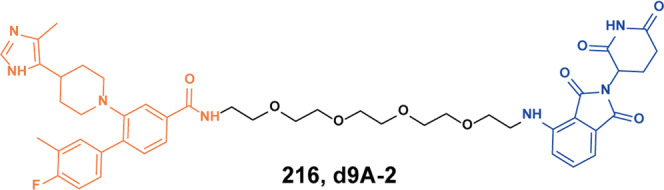
The representative PROTAC targeting SLC

In 2020, Furga group developed a SLC degrader **216** (**d9A-2**, Fig. 69) conjugated with a SLC9A1 inhibitor and pomalidomide with an optimized PEG linker.^420^ The degrader **216** (**d9A-2**) was a first-class SLC PROTAC, which could induce the high-efficiency degradation of its homologous targeted SLC9A1, as well as the degradation of other SLC9 members, such as the transporters SLC9A2 and SLC9A4. The proliferation inhibitory activity of the degrader **216** (**d9A-2**) on KBM7 cells was consistent with the evidence of degradation in the cytotoxicity test. In addition, the degrader **216** (**d9A-2**) treatment resulted in a variety of cancer cell intracellular pH (pHi) restoration obstacles and toxicity.

#### SMARCA2/4

The ATP-dependent chromatin remodeling complex BAF/PBAF changes the position and composition of nucleosomes through slipping and elimination to achieve dynamic regulation of chromatin structure. About one-fifth of tumors occur are closely related to the complex somatic mutations. The studies have found that two ATPases in the complex SMARCA2/4 are potential cancer treatment targets.^421^ SMARCA4 plays a tumor suppressor effect in solid tumors, but in AML it is necessary to maintain the oncogenic transcription program and drive proliferation.^422^ It has been reported that the double allosteric inhibitor of SMARCA2/4 ATP enzyme activity effectively inhibited the proliferation of the SMARCA4 mutant xenograft model.^423^

**Fig. 70:**
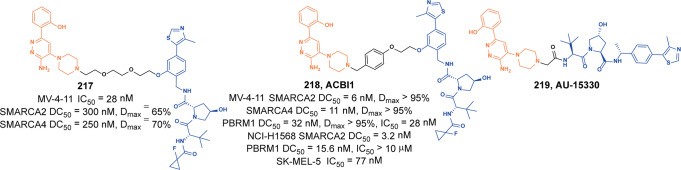
The representative PROTACs targeting SMARCA2/4

In 2019, Ciulli group developed a series of SMARCA2/4 degraders conjugated with a bromodomain ligand and VHL ligand.^424^ The degrader **217** (Fig. 70) successfully induced the moderate efficient degradation of SMARCA2/4 in MV4-11 cells with the *D*_max_ about 65–70%. Guided by high-resolution ternary complex crystal structures of SMARCA2^BD^-**217**-VCB, they also designed and developed the degrader **218** (**ACBI1**, Fig. 70), a potent and cooperative degrader of SMARCA2, SMARCA4, and PBRM1. The degrader **218** (**ACBI1**) could effectively induce the degradation of SMARCA2, SMARCA4, and PBRM1 in MV4-11 cells with the DC_50_ value of 6, 11, and 32 nM, respectively.

In 2021, Chinnaiyan group also linked the same bromodomain ligand with VHL and developed a degrader **219** (**AU-15330**, Fig. 70) for SMARCA2 and SMARCA4.^425^ The degrader **219** (**AU-15330**) could induce the degradation of SMARCA2 and SMARCA4 in HEK293 and HeLa cells, but it also showed a certain degradation activity to PBRM1.

#### SRC-1

Steroid receptor coactivator (SRC) is a type of transcription coactivator, which usually contains three subtypes, namely SRC-1, SRC-2, and SRC-3.^426^ SRC-1 is the first identified transcription coactivator that can promote the activity of various transcription factors (TF), such as estrogen receptor α, progesterone receptor and so on. As a coactivator, SRC-1 can not only promote the effect of transcription factor, but also promote the formation of protein complexes, so SRC-1 plays an important role in the organism.^427^ Studies have shown that the expression level of SRC-1 is low in normal physiological body, and it is abnormally activated and highly expressed in tumor cells. Therefore, SRC-1 is considered to be an oncogenic protein. Although corresponding inhibitors have been developed for SRC, most of them target SRC-1 and SRC-3. The inhibitors are lack selectivity to SRC-1 and are prone to cause side effects.^428^ Therefore, using PROTAC technology to induce degradation of SRC-1 protein has become a promising method.

**Fig. 71:**
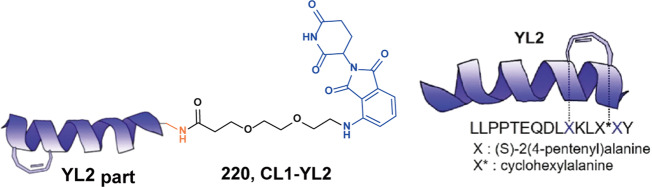
The representative PROTAC targeting SRC-1

In 2020, Lim group designed and synthesized the degrader **220** (**CL1-LY2**, Fig. 71) that targeted SRC-1 based on specific SRC-1 ligand stapled peptide **YL2** and pomalidomide.^429^ Although the SRC-1 binder was stapled peptide, it showed good binding affinity to SRC-1. In MDA-MB-231 cells, the degrader **220** (**CL1-LY2**) could induce degradation of SRC-1 protein at 10 μM, while it could not induce degradation of SRC-1 protein in Colo205 cells with low CRBN expression. In addition, it did not induce degradation of SRC-3, indicating that it had good selectivity.

#### Tyrosinase

Tyrosinase is the main rate-limiting enzyme of melanin synthesis, and it plays an important role in skin protection and pigmentation. So far, almost all tyrosinase inhibitors are based on cheap and easily available mushroom tyrosinase (mTYR) obtained through in vitro screening, such as hydroquinone, arbutin, l-ascorbic acid, ellagic acid, and tranexamic acid.^430^ However, most tyrosinase inhibitors have certain side effects. For example, hydroquinone is toxic to human cells and can cause skin irritation and bone marrow toxicity, l-ascorbic acid is easily sensitive to heat, ellagic acid is insoluble and has poor bioavailability.^431^ In addition, in order to produce an inhibitory effect, tyrosinase inhibitors need to constantly occupy the active site of the tyrosinase, but high doses can cause undesirable off-target effects and cause damage to the skin.^432^

**Fig. 72:**
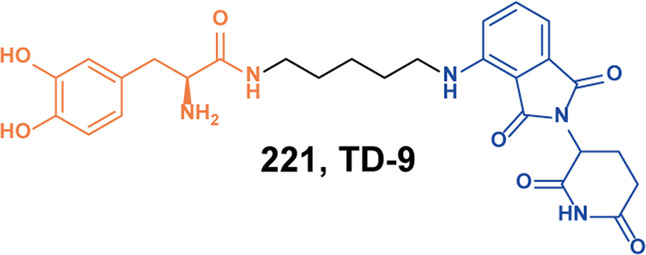
The representative PROTAC targeting tyrosinase

In 2021, Tang group designed and synthesized a tyrosinase degrader based on **L-Dopa** and pomalidomide.^433^ The degrader **221** (**TD9**, Fig. 72) had the best effect in inducing degradation of human tyrosinase, and it also showed obvious dose and time-dependent manner. It also proved that the degradation mechanism was achieved through the protein–ubiquitin system. In addition, they also used the low-toxicity degrader **221** (**TD9**) on zebrafish to reduce the synthesis of zebrafish melanin, highlighting the potential for the treatment of tyrosinase-related diseases.

## PROTACs targeting virus-related targets

### Pan-coronavirus antiviral

**Indomethacin** is a non-steroidal anti-inflammatory drug (NSAID) with anti-inflammatory, analgesic, and antipyretic properties. Its pharmacological effects are not fully understood. It is not only related to the activities of cyclooxygenase 1 and 2, but also inhibit the biosynthesis of phospholipase A2 (PLA-2) and microsomal prostaglandin E synthase 2 (mPGES-2).^434^ Studies have shown that it had good activity against coronavirus infections, so it had great development potential in the field of anti-inflammatory and antiviral.^435^

**Fig. 73:**
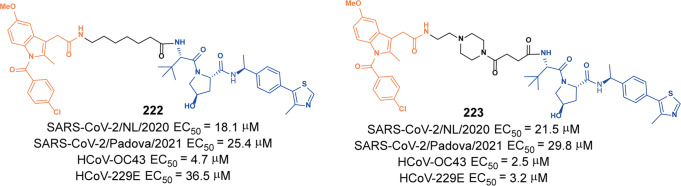
The representative PROTACs targeting pan-coronavirus antiviral

In 2021, Goracci group designed a series of PROTACs based on **Indomethacin** and VHL,^436^ it was found that the degrader **222** (Fig. 73 and **223** (Fig. 73) had good inhibitory activities against a variety of coronaviruses through activity screening. Molecular simulations showed that PGES-2 may be the potential target of INM-based antiviral PROTAC.

### SARS-CoV-2

Severe acute respiratory syndrome coronavirus 2 (SARS-CoV-2) has infected more than 240 million individuals worldwide, and the death toll has exceeded 4.9 million and is still rising. Although some vaccines have been widely used to prevent COVID-19, due to the high mortality and the occurrence of mutations, the development of curable drugs has become more and more important. Although some drugs have entered the clinical trial stage, the therapeutic effect is still to be observed. So using new technologies to develop new treatments has become a strategy.

**Telaprevir**-based PROTAC has been reported to be used for the degradation of HCV NS3/4A protease. The Mpro protein plays an important role in SARS-CoV-2, so PROTAC technology can be used to induce degradation of the Mpro protein so as to achieve the purpose of curing SARS-CoV-2. The applicability of PROTAC technology on SARS-CoV-2 has been discussed recently by different groups.

**Fig. 74:**
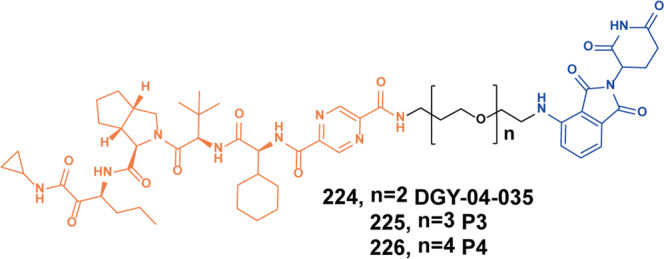
The representative PROTACs targeting SARS-CoV-2

In order to apply PROTAC technology to the treatment of SARS-CoV-2, Sobhia group firstly assessed the similarity of NS3/4A and Mpro residues. The sequence alignment results showed that **Telaprevir** could be used in the design of Mpro degraders. Subsequently, the degraders **224**–**226** (Fig. 74) which were most likely to induce degradation of Mpro protein was generated by constructing a ternary complex model and simulating the protein–protein interaction.^437^

## PROTACs for treating immune disorders

### HDAC3

Histone deacetylases (HDACs) play important roles in inflammatory diseases like asthma and chronic obstructive pulmonary disease (COPD). Currently, eighteen HDAC isoenzymes, which can be divided into four classes, have been discovered. Among them, class I HDACs, containing HDAC1, 2, 3, and 8, are well-known for their importance in cell motility, immunoregulation, and proliferation.^438^ More and more studies have shown that HDAC3 is closely related to the occurrence and development of tumors.^439^ However, because HDAC3 has a highly conserved catalytic domain, it is quite challenging to develop an isoenzyme-specific HDAC3.

**Fig. 75:**
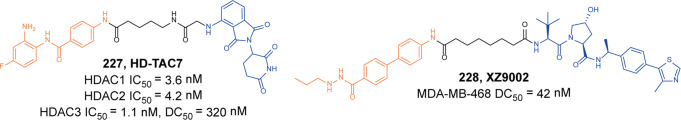
The representative PROTACs targeting HDAC3

In 2020, Dekker group reported the development of a novel PROTAC targeting HDAC3 by tethering the CRBN ligand pomalidomide and o-aminoanilide-based class I HDAC inhibitors.^440^ The degrader **227** (**HD-TAC7**, Fig. 75) induced the degradation of HDAC3 with a DC_50_ value of 0.32 μM in RAW 264.7 macrophages, whereas without influence on HDAC1 and 2. In the same years, Liao group developed a novel VHL-based degrader **228** (**XZ9002**, Fig. 75) that could selectively and potently induce the degradation of HDAC3 in a dose and time-dependent manner.^441^ Furthermore, the degrader **228** (**XZ9002**) had potent antiproliferative activity against cancer cells.

### H-PGDS

Hematopoietic prostaglandin D synthase (HPGDS) is an attractive target for the treatment of a variety of diseases, including allergic diseases and Duchenne muscular dystrophy.^442^ To date, several types of H-PGDS inhibitors have been developed as therapies for allergic and inflammatory responses. However, no HPGDS inhibitors have yet been approved into clinical studies.^443^ Therefore, the development of novel agents having other modes of action to modulate the activity of H-PGDS is required.

**Fig. 76:**
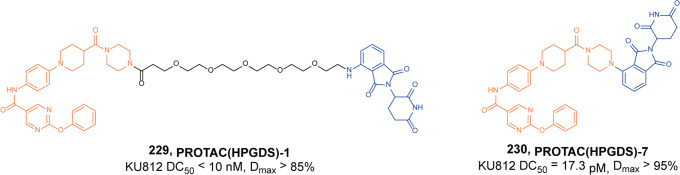
The representative PROTACs targeting H-PGDS

In 2021, Demizu group reported the first H-PGDS degrader **229** (**PROTAC(HPGDS)-1**, Fig. 76) by coupling the HPGDS inhibitor **TCF-007** with CRBN ligand pomalidomide.^444^ The degrader **229** (**PROTAC(HPGDS)-1**) effectively induced the selective degradation of H-PGDS protein and suppression of prostaglandin D2(PGD2) production. It was worth noting that the degrader **229** (**PROTAC(HPGDS)-1**) continued to inhibit the production of PGD2 after the removal of the drug, whereas the production of PGD2 was restored after the removal of **TFC-007**. In the same year, by using a docking simulation of the ternary complex of H-PGDS-**PROTAC (H-PGDS)-1**-cereblon, this group also successfully developed the degrader **230** (**PROTAC(H-PGDS)-7**, Fig. 76) without any linker.^445^ The degrader **230** (**PROTAC(H-PGDS)-7**) exhibited potent and selective degradation of H-PGDS with DC_50_ value of 17.3 pM after 6 h treatment, and potent suppression of prostaglandin D2 production in KU812 cells. In addition, the degrader **230** (**PROTAC(H-PGDS)-7**) had better inhibition of inflammatory cytokines than **TFC-007** in a Duchenne muscular dystrophy model using mdx mice with cardiac hypertrophy. Therefore, the degrader **230** (**PROTAC(H-PGDS)-7**) was expected to play a role in biological research and clinical treatment.

### IRAK1

IRAK1, a class of serine-threonine protein kinases associated with IL-1R and TLR signaling, plays a key role in initiating the innate immune response against foreign pathogens. Upon activation, TLR or IL/1Rs dimerizes and allows its intracellular structural domain to recruit the myeloid differentiation primary response 88 (MyD88) adaptor protein. MyD88 molecules oligomerize through the N-terminal death domain (DD) and recruit IRAK containing the DD structural domain to form a macromolecular signaling complex called myddosome, which leads to the autophosphorylation and activation of IRAK4. Activated IRAK4 phosphorylates IRAK1 and/or IRAK2, followed by the complementation of TNFR-associated factor 6 (TRAF6) into the myddosome. Activated TRAF6 is then released into the cytoplasm, where it triggers activation of the IκCB kinase (IKK)-nuclear factor-κB (NF-KB) cascade and MAP kinase (MAPK) signaling pathways.^446–449^ Despite some advances in the study of IRAK1, IRAK1 remains a challenging target in the field of traditional small-molecule inhibitors, partly due to the lack of understanding of the structural domains primarily responsible for its scaffolding function.

**Fig. 77:**
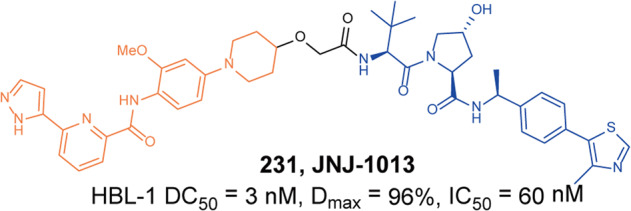
The representative PROTAC targeting IRAK1

In 2021, Dai group first reported a series of IRAK1-targeting PROTAC based on IRAK1 inhibitor **JH-I-25** and VHL ligands.^450^ The most potent degrader **231** (**JNJ-1013**, Fig. 77) showed better kinase selectivity and potent IRAK1 degradation activity with DC_50_ of 3 nM and *D*_max_ of 96% in HBL-1 cells. In ABC DLBCL cells with the MyD88^*L265P*^ mutation, the degrader **231** (**JNJ-1013**) effectively blocked downstream signaling pathway of IRAK1, induced apoptosis, and displayed stronger antiproliferative effects compared to IRAK1 inhibitors, indicating that the scaffold function of IRAK1 played a key role in ABC DLBCL cell survival.

### IRAK3

IRAK3 (interleukin-1 receptor-associated kinase 3, also known as IRAK-m) is a class 1 pseudokinase member of the IRAK family(along with IRAK1, IRAK2, and IRAK4).^451^ IRAK3 is believed to perform biological functions through a non-kinase-catalyzed scaffolding action.^452^ The expression of IRAK3 is mainly restricted to leukocytes, and it has been reported to inhibit pro-inflammatory signaling in innate leukocytes(monocytes, macrophages and neutrophils).^453^ Knockdown of IRAK3 in mice leads to reprogram of myeloid cells toward immune activation and promotes proliferation of effector T cells, which in turn helps to overcome immune suppression and enhance the host response to checkpoint inhibition.^454,455^ Since IRAK3 is a pseudokinase, it is unclear whether the molecule bound to IRAK3 has a functional role, and it is essential to employ some techniques to address this issue.

**Fig. 78:**
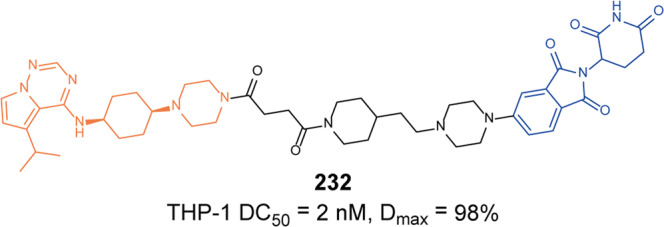
The representative PROTAC targeting IRAK3

In 2020, Edmondsonet group first reported potent and selective IRAK3 degraders by conjugating a byproduct of IRAK4 inhibitors with CRBN and VHL ligands.^456^ the degrader **232** (Fig. 78) induced the degradation of IRAK3 with DC_50_ of 2 nM and *D*_max_ of 98% in THP-1 cells and primary macrophages. Although not yet fully optimized, the degrader **232** was an excellent tool to study the biology of IRAK3 degradation in vitro.

### IRAK4

Playing a key role in both toll-like receptors (TLRs) and interleukin-1 receptors (1L-1R) signaling pathways, IRAK4 has been identified as a potential drug target involved in the innate immune process and has attracted widespread interest as a novel modality for the treatment of inflammatory diseases and oncology.^457^ Upon activation of TLRs or 1L-1R receptors, MYD88 will be recruited to the TIR domain of the receptor, followed by the IRAK family to form a myddosome complex. IRAK4 bound to MYD88 will lead to phosphorylation of IRAK1 and IRAK2 in the complexed and subsequent activation of the IkB kinase (IKK)-nuclear factor-kB (NF-kB) and the mitogen-activated protein kinase (MAPK) signaling pathways. Several reports have revealed an important role of IRAK4 in myddosome assembly in addition to its kinase catalytic function and its kinase backbone function, suggesting that overall inhibition or elimination of IRAK4 has a great potential to completely eliminate IL-1R/TLR assembly or the resulting signaling.^458^ Several investigators have begun to explore to use PROTACs to induce the degradation of IRAK4.

**Fig. 79:**
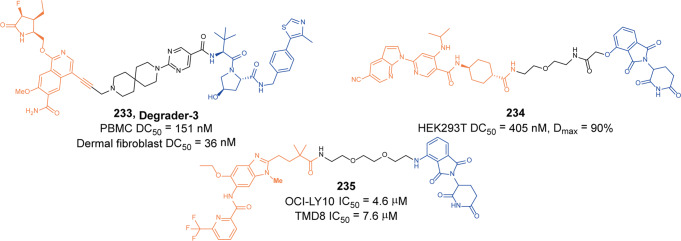
The representative PROTACs targeting IRAK4

In 2019, Anderson group reported a series of PROTACs by incorporating IRAK4 ligand **PF-06650833** to different E3 ligases.^459^ The VHL-based degrader **233** (**Degrader-3**, Fig. 79) induced the most potent degradation of IRAK4 with a DC_50_ of 151 nM in PBMCs and 36 nM in dermal fibroblast cells. However, the degrader **233** (**Degrader-3**) did not show additional potential benefit upon TLR7/8 stimulation in PBMCs compared to **PF-06650833**. And the inhibition of IL-6 and TNF-α was also not observed in IL-1β stimulated human dermal fibroblasts. Nevertheless, the possibility of targeting IRAK4 kinase allowed new therapeutic chances to treat autoimmune and oncological diseases.

In 2020, Dai group reported a series of potential PROTACs based on CRBN ligands and a highly selective IRAK4 inhibitor for the degradation of IRAK4.^460^ These PROTACs showed moderate affinities to CRBN-DBB1 with binding affinity values ranging from 490 to 1080 nM. Among all of the PROTACs, the degrader **234** (Fig. 79) only induced the degradation of IRAK4 protein, displayed a remarkable selectivity of IRAK4 degradation in OCI-LY3 ABC DLBCL cells in a proteome-wide analysis. The degrader **234** induced the rapid degradation of IRAK4 with a DC_50_ value of 405 nM in HEK293T cells after 24 h of treatment. The results showed that neither IRAK4 kinase inhibition nor degradation resulted in cell death or growth inhibition, suggesting that the role of IRAK4 in the survival of ABC DLBCL cells was redundant. IRAK4 PROTACs characterized in this study provided useful tools for understanding IRAK4 protein scaffolding function, which was previously unachievable through pharmacological perturbation.

In 2021, Duan group disclosed a set of PROTACs by conjugating an IRAK4 inhibitor to pomalidomide via flexible linkers, including hydrophilic polyethylene glycol (PEG) and hydrophobic all-carbon chains.^461^ The most potent degrader **235** (Fig. 79) induced the degradation of IRAK4 in OCI-LY10 and TMD8 cells in a dose and time-dependent manner. In addition, the degrader **235** efficiently blocked the IRAK4-NF-κB signaling pathway and displayed a substantial advantage in inhibiting the growth of cells expressing the MYD88^*L265P*^ mutation compared with the parent IRAK4 inhibitor.

## PROTACs for treating neurodegenerative diseases

### GSK-3β

Glycogen synthase kinase 3 (GSK-3) belongs to a multifunctional serine/threonine protein kinase under the phosphotransferase family. There are two isomers of mammalian GSK-3, namely GSK-3α and GSK-3β.^458^ GSK-3β controls the synthesis of glycogen to regulate the metabolism of glycogen, and affects the permeability of mitochondria and the releases of cytochrome C to regulate apoptosis. GSK-3β also acts on activation of the transcription factors and functions in many biological processes including embryonic development, cell differentiation, and insulin response.^459^ GSK-3β is abundant in brain and is associated with many neurodegenerative diseases and neurological disorders. Recently, GSK-3β has been described as a tumor suppressor due to its ability to phosphorylate the pro-oncogenic moleculars that affect the cell cycle and DNA repair.^460^

**Fig. 80:**
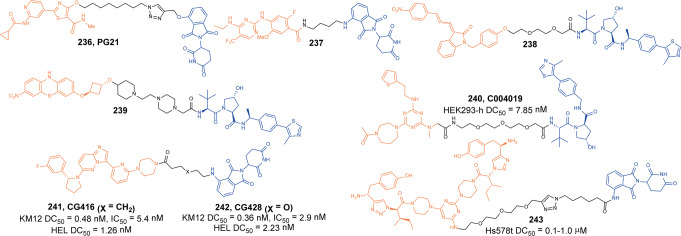
The representative PROTACs targeting neurodegenerative diseases

The first GSK-3β degrader was reported by Sun group in 2021.^461^ The degrader **236** (**PG21**, Fig. 80) was a powerful GSK-3β degrader, it could effectively induce the degradation of GSK-3β in a dose-dependent manner, which could induce 44.2% protein degradation at 2.8 μM. Mechanistic investigation determined that the degrader **236** (**PG21**) needed to rely on the intracellular UPS to induce GSK-3β degradation. In addition, it protected against glutamate-induced cell death in HT-22 cells.

### LRRK2

PARK8 is one of the genes associated with Parkinson's disease (PD), which encodes leucine-rich repeat kinase 2 (LRRK2).^462^ To date, a large number of disease-associated LRRK2 mutations have been identified and five mutations (R1441C, R1441G, Y1699C, G2019S, and I2020T) are associated with PD pathogenesis. LRRK2 mutations, particularly the most common G2019S mutation, are observed in patients with autosomal dominant PD and apparently disseminated PD. Pathogenic mutations in the LRRK2 gene increase LRRK2 kinase activity, and LRRK2 kinase inhibitors are neuroprotective in preclinical models of Parkinson's disease. These findings make LRRK2 to be one of the important targets for the treatment of PD and targeting LRRK2 has become an effective treatment for PD.^463^

In 2020, Dömling group firstly reported the PROTACs targeting LRRK2 degradation.^464^ Series of potential LRRK2 PROTACs were constructed based on the LRRK2 inhibitors **PF-06447475** or **GNE-7915** and CRBN ligand pomalidomide. For example, the degrader **237** (Fig. 80) was a representative degrader, although the PROTACs were effective in inhibiting kinase activity and performed good cell permeability, the western blotting did not show any significant changes in LRRK2 protein level after the PROTACs treatment, which suggested that these PROTACs were unable to induce degradation of LRRK2.

### α-Synuclein

α-Synuclein is mainly expressed in human brain cells and central nervous system cells. It is a presynaptic 140 amino acid that has no secondary structure in many tissues. The misfolding and aggregation of these proteins is a pathological feature of Parkinson's disease (PD) and other neurodegenerative diseases.^465^

In 2020, Crew group published the patent on α-Synuclein degraders.^466^ They designed and synthesized different degraders and tested their degradation activity in HEK293 TREX α-synuclein A53T cells. The experimental results showed that the degrader **238** (Fig. 80) and **239** (Fig. 80) induced degradation of α-Synuclein, and the degradation activity were 30–65% at 1 µM, which proved neuronal diseases related to α-Synuclein accumulation and aggregation (AD, PD, Dementia, etc.) could be treated by targeted degradation of α-Synuclein.

### Tau

Tau is a cytoskeletal protein that regulates the formation and stability of microtubules by binding to neuronal microtubules, and maintains the structure and function of cytoskeletal neurons. AD and other neurological diseases can occur when the tau protein is defective and cannot sufficiently stabilize microtubules.^467^ Hyperphosphorylation and aggregation of tau protein destroy the microtubule structure, leading to neurofibrillary tangles in AD neurons. More and more evidence showed that the hyperphosphorylation of tau protein and the increase of *p*-tau protein aggregation are closely related to the occurrence and development of AD.^468^

In 2021, Wang group developed a novel tau protein degrader **240** (**C004019**, Fig. 80) by recruiting VHL E3 ligase.^469^ It showed selective and potent tau protein degradation both in vitro cellular models (HEK293 and SH-5Y5Y) and in vivo mice models (hTau-transgenic and 3xTg-AD). Most importantly, both single and multiple doses (once per 6 days for a total of five times) of subcutaneous degrader **240** (**C004019**) significantly reduced tau levels in the brains of wild-type, hTau-transgenic, and 3xTg-AD mice with improved synaptic and cognitive function. Therefore, the degrader **240** (**C004019**) could be considered as a promising drug candidate in related diseases.

### TRKA

TRKs are receptor tyrosine kinases encoded by the NTRK1/2/3 genes and contain mainly three members(TRKA/B/C), which are associated with the development and function of neuronal tissues.^470^ When the extracellular structural domains of TRKs bind to their corresponding ligands, they will induce dimerization and activation of the intracellular kinase domains of TRKs thereby providing signals to downstream PI3K/AKT, RAF/MEK/ ERK, and phospholipase C gamma(PLCγ).^471^ This ultimately leads to constitutive activation of the TRK pathway and promotes cell proliferation, survival, and malignant transformation.

In 2020, Chen group reported a series of selective and potent TRKA degraders by conjugating a pan-TRK inhibitor **GNF-8625** to CRBN ligand thalidomide.^472^ The degrader **241** (**CG416**, Fig. 80) and **242** (**CG428**, Fig. 80) were the most promising degraders, whose DC_50_ were 0.48 nM and 0.36 nM in KM12 cells. The degrader **241** (**CG416**) and **242** (**CG428**) also induced degradation of wild-type TRKA with DC_50_ values of 1.26 nM and 2.23 nM in HEL cells. These two degraders exhibited more potent inhibition of cell growth than the TRK inhibitor **GNF-8625**. Further, they also exhibited good plasma exposure in mice. These results suggested that they were valuable chemical tools for investigating TRKA function.

### TRKC

TRKC, a member of the TRK family, is closely related to the development and function of neuronal tissues. The decrease in TRKC has been found in a variety of neurodegenerative diseases including AD, PD, and HD.^473^ In addition, overexpression and aberrant activation of TRKC has been observed in a variety of human tumors. Aberrant activation of TRKC and TRKC fusion proteins significantly induces growth rate, epithelial–mesenchymal transition(EMT), and oncogenic capacity through constitutive activation of the PI3K-AKT, RAS-MAP kinase (MAPK), and JAK2-STAT3 pathways.^474^

In 2019, Zhao group reported the first TRKC PROTACs based on the TRKC binder **IY-IY** (*K*_d_ = 112 nM) and different E3 ligase ligands.^475^ pomalidomide-based degrader **243** (Fig. 80) showed better degradation activity of TRKC with an estimated DC_50_ of 0.1–1.0 μM. TRKC degraders may provide opportunities for alternative treatment strategies for TRKC-related diseases.

## Other PROTACs

### Engineered Cas protein

Cas protein (CRISPR-associated protein) is a type of nuclease in the CRISPR (Clustered regularly interspaced short palindromic repeats) system, which plays an important role in the adaptive immune function of CRISPR system.^476,477^ It assists CRISPR-Cas systems in targeting and ultimately degrading foreign nucleic acids under the guidance of CRISPR RNA (crRNA).^478,479^ Cas protein can be divided into two classes (Classes 1 and 2) and six types (types I–VI) based on their specific mechanisms of guiding RNA biogenesis and targeting interference.^480,481^ There are several most widely used examples such as Cas9 (type II), Cas12 (type V), and Cas13 (type VI).^482–484^ Although CRISPR-Cas system has become a powerful tool to manipulate the human genome for gene therapy, it may still cause some side effects owing to off-target edits.^485^

**Fig. 81:**
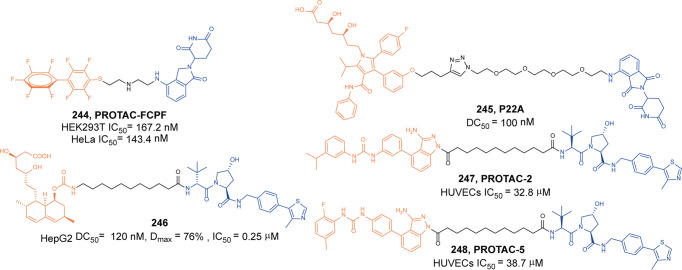
The representative PROTACs targeting Cas protein, HMGCR and VEGFR2

In 2021, Cheng group developed engineered Cas^FCPF^ proteins (Cas9, dCas9, Cas12, and Cas13) by inserting a Phe-Cys-Pro-Phe(FCPF) amino acid sequence (known as the π-clamp system) into Cas proteins, which could be labeled by perfluoroaromatics carrying thefluorescein. Then they designed a perfluoroaromatics-based degrader **244** (**PROTAC-FCPF**, Fig. 81) by using lenalidomide as E3 ligases ligand. The degrader **247** (**PROTAC-FCPF**) could induce the degradation of Cas9^FCPF^ with IC_50_ of 167.2 nM in HEK293T cells and 143.4 nM in HeLa cells.^486^

### HMGCR

HMG-CoA reductase (HMGCR) is an eight-channel transmembrane protein located in the endoplasmic reticulum (ER). It is the rate-limiting enzyme in the cholesterol biosynthetic pathway and a classical drug target of statins which can lower the cholesterol and preventing cardiovascular disease (CVD).^487,488^ Statins competitively bind to the active site of the enzyme through its HMG-like part to prevent the production of mevalonate and downstream derivatives including cholesterol. However, statins may cause a compensatory increase in protein expression^489^ which limits the maximal effectiveness of the drug and provoking some side effects including skeletal muscle damage.^490,491^ With the development of PROTACs, a promising therapeutic approach to induce degradation of proteins of interests, it might be able to solve this problem by chemical knockdown instead of small-molecule inhibition.^492^

The first degrader of HMGCR was reported by Rao group in 2020.^493^ They synthesized a class of PROTACs by linking **Atorvastatin** with CRBN ligands. The most potent and selective degrader **245** (**P22A**, Fig. 81) induced the degradation of HMGCR with DC_50_ of 0.1 μM. HMGCR was the first ER-localized and polytopic transmembrane protein successfully degraded by PROTAC. It highlighted the potential application of PROTAC technique in treating hypercholesterolemia and CVD.

In 2021, Xiang group developed the VHL-based PROTAC targeting HMGCR.^494^ By combining VHL with **Lovastatin** they demonstrated the most potent degrader **246** (Fig. 81) with IC_50_ of 0.25 µM. Furthermore, they conducted experiments to evaluate the HMGCR degradation potency in vivo and confirmed that the degrader **246** could be the active ingredient to lower cholesterol in vivo. The VHL-based degrader **246** was the first PROTAC targeting HMGCR with oral activity.

### VEGFR2

Vascular Endothelial Growth Factor Receptor 2 (VEGFR2, previously known as KDR or Flk-1) is a member of VEGFR family of receptor tyrosine kinases (RTK)^495,496^ and plays an important role in angiogenesis. It is a highly active kinase and can stimulate many signaling pathways and varieties of biological responses. VEGFR2 is the key receptor that mediate the major growth and permeability actions of VEGF. Mice lacking VEGFR2 are unable to develop a vasculature and have rare endothelial cells.^497,498^ As a member of RTKs, the basic activation principles of VEGFR2 can be separated into three parts. The first procedure is dimerization of receptor monomers mediated by ligand, followed by the transphosphorylation with dimerized receptors and docking of signaling proteins to receptor phosphotyrosines.^499,500^ Occupancy-based RTK inhibitors are the traditional methods to intervene the pathological angiogenesis. However, owing to the high concentration and potential off-target side effects, it is necessary to develop PROTACs to treat angiogenesis.

In 2020, Zhang group designed several PROTACs based on the previously developed anti-angiogenesis agents.^501^ They combined their novel potent angiogenesis inhibitors **S7** (biphenyl-aryl ureas incorporated with salicyladoxime) and E3 ubiquitin ligases ligand (**VH032**) with different lengths of dicarboxylic acid. The most potent degrader targeting VEGFR2 were degrader **247** (**PROTAC-2**, Fig. 81) and **248** (**PROTAC-5**, Fig. 81) with IC_50_ of 32.8 μM and 38.7 μM. The degrader **247** (**PROTAC-2**) could specifically reduce the protein levels of VEGFR2 and normalize the abnormal vessels without significant cytotoxicity.

## New technologies based on PROTACs

In the above space, we summarize the main progress in the PROTAC field in the past 2 years. Compared with traditional small-molecule inhibitors, although PROTACs have shown obvious advantages, they also have similar disadvantages as small-molecule inhibitors. First, because PROTAC is developed based on POI inhibitors, it still has a certain degree of off-target effect. Secondly, due to the large molecular weight of PROTAC, it has poor cell-membrane permeability and poor pharmacokinetic(PK) properties, which greatly reduces its biological and therapeutic effects. In addition, although some PROTACs can efficiently induce the degradation of target proteins, their biological effects are so weak that they do not have an effective effect on the disease. Finally, most proteins do not have corresponding small-molecule binders for designing PROTACs, such as most transcription factors, which play an important role in the occurrence and development of diseases. The inhibitors of transcription factors are few, resulting in there is no binder available when designing PROTACs targeting transcription factors. This greatly limits the application of PROTAC technology. Therefore, it is of great significance to integrate other drug design strategies into PROTAC technology to solve the above shortcomings.

In order to solve the problems mentioned above, different types of PROTAC technologies have emerged in recent years, such as Antibody-PROTAC, Aptamer-PROTAC, Dual-target PROTAC, Folate-caged PROTAC, and Transcription factor-PROTACs, etc. Although these technologies are based on PROTACs, they all have advantages that are different from traditional PROTAC technologies. For example, Antibody-PROTAC can overcome the shortcomings of PROTACs off-target effect and weak tissue specificity, Aptamer-PROTAC can improve the cell-membrane permeability and pharmacokinetic(PK) properties of PROTACs. Although these new PROTAC-based technologies achieved degradation on very few proteins, they undoubtedly injected vitality into the development of PROTACs. So we will briefly introduce these new technologies.

### Antibody-PROTAC

Antibody-PROTAC is a new strategy to explore the combination of PROTAC and antibodies to assemble new Antibody-PROTAC conjugates. This technology can achieve the specific degradation of proteins in different cells and tissues, thereby optimizing the therapeutic window and maximizing the treatment window, reducing the side effects of broad-spectrum PROTAC and increasing its potential as a drug or chemical tool. What’s more, although examples of intravenous (IV) and oral (PO) administration have been reported, most of the currently reported PROTACs are usually delivered to animals by subcutaneous (SC) or intraperitoneal (IP) administration routes,^502–505^ which result in their low bioavailability. Contrastly, the antibody-PROTAC conjugate technology can be used to overcome the potential delivery difficulties of PROTACs.

**Fig. 82:**
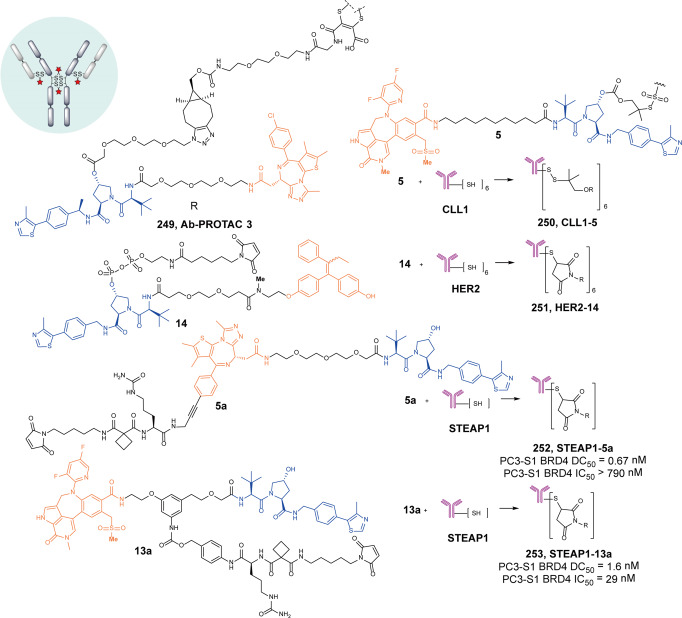
The representative PROTACs of antibody-PROTAC

In 2020, Tate group reported a trastuzumab-PROTAC conjugate **249** (**Ab-PROTAC 3**, Fig. 82) based on trastuzumab and BRD4 degrader.^506^ Its antibody connection position is at the end of E3 ligase, and the antibody linker can be hydrolyzed in the cell and then releases active PROTAC and induces the degradation of BRD4 protein. They found that the conjugate **249** (**Ab-PROTAC 3**) only induced the degradation of BRD4 protein in HER2-positive breast cancer cell lines (SK-BR-3 and BT474), but not in HER2-negative cells (MCF-7 and MDA-MB 231). Significant degradation of BRD4 protein was observed in HER2^+^ cells incubated with 50 nM and 100 nM conjugate **249** (**Ab-PROTAC 3**) for 4 hours, but not detected at any concentration in the HER2^−^ cell lines. This study verified the concept of tissue-specific Ab-PROTAC inducing BRD4 protein degradation, overcoming the limitations and selectivity of PROTACs, and laying the foundation for the development of new type PROTAC.

Researchers from Genentech have also developed a series of Ab-PROTAC conjugates based on different antibodies and BRD4 degraders. In 2020, they reported an antibody-PROTAC conjugate **250** (**CLL1-5**, Fig. 82) based on **CLL1** (C-type lectin-like molecule-1,^507^ also known as C-type lectin domain family 12 member A(CLEC12A) and the BRD4 degrader developed by themselves. To avoid the aggregation problem caused by high lipophilicity of linker, they linked six BRD4 degraders to CLL1. After antibody-mediated delivery, the higher drug loading of these conjugates could increase the intracellular drug concentration. Afterward, the conjugate **250** (**CLL1-5**) showed significant antitumor activity in the HL-60 AML xenograft model. All of these results proved that the mechanism of the conjugate **250** (**CLL1-5**) was the degradation of BRD4 protein induced by PROTAC after antibody-mediated delivery. At the same time, they also reported another antibody-PROTAC conjugate **251** (**HER2-14**, Fig. 82) based on HER2 and ERa degrader.^508^ It was subsequently proved that the antibody-PROTAC conjugates could be delivered dependently to MCF-7-neo/HER2 cells, and the conjugate **251** (**HER2-14**) had the best effect on ERα degradation, with a DC_50_ of 0.03 µg/mL and a *D*_max_ of 94%. By synthesizing different antibody-PROTAC conjugates and testing their degradation activities, they proved that the linker between the antibody and PROTAC could adjust the stability of the conjugate and regulate the efficiency of releasing the degrader in the cell.

In 2021, they successively reported two types of antibody-PROTAC conjugates based on different BRD4 degraders and antibodies. First, they designed a series of BRD4 protein degraders based on **JQ1**. On this basis, they used different linkers to connect the degraders to the antibodies to synthesize antibody-PROTAC conjugates. The antibodies they used included **STEAP1** (an antibody that recognizes the six transmembrane epithelial antigen of the prostate 1), **CLL1,** and **HER2**.^509^ It was found that in the PC-3-S1 cell line, the conjugate **252** (**STEAP1-5a**, Fig. 82) showed the best BRD4 degradation activity with a DC_50_ of 0.67 nM, but its antiproliferative activity was poor, with an IC_50_ value more than 790 nM. The possible reason may be the poor cell-membrane-permeability properties. Subsequently, on the basis of previous researches, by replacing the structure of the BRD4 degrader and synthesizing a series of antibody-PROTAC conjugates again they found the conjugate **253** (**STEAP1-13a**, Fig. 82) had good BRD4 protein-degradation activity,^510^ and its DC_50_ was 1.4 nM. At the same time, it showed good antiproliferative activity against PC-3-S1 cell line with IC_50_ of 29 nM. In addition, they also evaluated antiproliferative activity of the conjugate **253** (**STEAP1-13a**) in PC-3-S1 and HL-60 xenograft models and found that it achieved antigen-dependent antitumor activity in both prostate cancer and AML xenograft models.

### Aptamer-PROTAC conjugates

In addition to antibody-PROTAC, Aptamer-PROTAC is also a new PROTAC technology that can improve water solubility, membrane permeability, and tumor targeting, which are typical disadvantages of traditional PROTACs.^511^ Aptamer is single-stranded nucleic acid with complex three-dimensional structures, which mainly include stems, loops, hairpins, and G4 polymers.^512^ They bind to target proteins with high specificity and affinity through special effects, including hydrogen bonding, van der Waals force, base stacking force and electrostatic effect.^513^ Due to special properties, aptamers are also called chemical antibodies by researchers. Compared with other targeting carriers, aptamers have the following advantages: (1) good tissue permeability, (2) good in vivo safety, (3) no obvious immunogenicity. They have been widely used in targeted therapy for human tumors.^514,515^

**Fig. 83:**
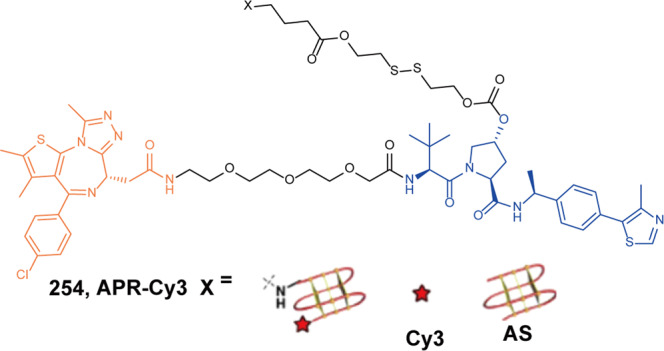
The representative PROTACs of aptamer-PROTAC conjugates

Inspired by the application of aptamers in targeted tumor therapy, in 2021 Sheng group designed and synthesized the first Aptamer-PROTAC conjugate (APC) by combining the aptamer with the BET protein degrader.^516^ They used a disulfide bond to connect the effective BET (BRD4) degrader and the nucleoside-dependent aptamer **AS** to synthesize the APC **254** (**APR-Cy3**, Fig. 83). The conjugate APC had a good effect on the BET degradation in MCF-7 breast cancer cells. It is the first time this study has proved that aptamer binding could help improve degrader targeting specificity, reduce toxicity, and enhance in vivo antitumor activity and protein-degradation activity. Therefore, the innovative APC technology established in this work may improve the drug similarity of traditional PROTACs and provide a new method for obtaining degraders which have better tumor tissue specificity and clinical efficacy.

### Dual-target PROTACS

In addition to using antibody-PROTAC and Aptamer-PROTAC to solve the targeting specificity and membrane permeability problems of PROTAC, how to improve the antitumor activity of PROTAC is also an urgent problem to be solved. But during the occurrence and development of cancer, there are usually many factors working together, including different kinds of kinases and growth factors, which can act independently or interfere with each other through signal networks.^517^ In the process of cancer treatment, tumor cells can easily obtain drug resistance by compensatory action or other signal pathways activation.^518^ Therefore, there are obvious limitations in drug treatment for a single target. In order to overcome the shortcomings, drug development for two or more targets has received more and more attention. Although “cocktail therapy” has achieved good clinical effects, long-term large doses and multiple use of different types of drugs have greatly reduced the compliance and quality of life of patients. Therefore, the development of single-drug-based dual-target drugs has become one of the strategies. The method is mainly to design a single molecule that combined two or more pharmacophores to target two or more antitumor targets at the same time. In the previous studies, there have been a large number of reports showing that the dual inhibitors based on this strategy can achieve good antitumor effects,^519–521^ and it has gradually become an alternative to combination therapy. The currently reported PROTACs are basically degraders designed based on only one protein. Although the one degrader can induce the degradation of different proteins,^294,522–525^ it is mostly caused by off-target effects of the protein ligands more than the rational design of multi-target degradation. Therefore, for two abnormal proteins in the same disease, it has become a way to treat the disease with a powerful degrader that is rationally designed based on two different ligands.

**Fig. 84:**
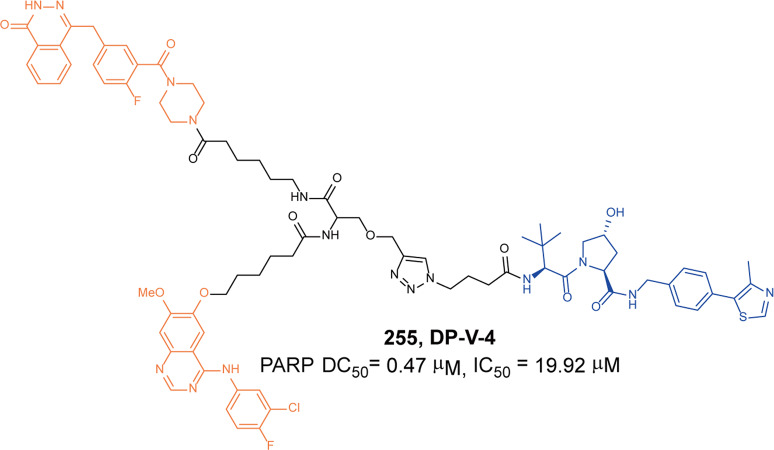
The representative PROTAC of dual-target PROTACs

In 2021, Li group used the E3 ligase ligand and the EGFR inhibitor **Gefitinib** and the PARP inhibitor **Olaparib** to form star-shaped dual-target degraders,^526^ and then evaluated their degradation activities of the two proteins in H1299 cells. They found that the compound **255** (**DP-V-4**, Fig. 84) showed the best dual-target degradation activity. Its degradation activity of PARP was slightly better than that of EGFR, and the DC_50_ of PARP was 0.47 µM. As the concentration increased, its degradation activity gradually increased. Meanwhile, its degradation activity of EGFR was weak and a higher concentration was required to achieve effective degradation. Subsequently, they tested the antiproliferative activity of the compound **255** (**DP-V-4**) in H1299 cells. Its IC_50_ was 19.92 µM, which was between EGFR inhibitor **Gefitinib** (IC_50_ = 6.56 µM) and PARP inhibitor **Olaparib** (IC_50_ = 35.93 µM). The weaker antiproliferative activity of the compound **255** (**DP-V-4**) may be due to the larger molecular weight of the degrader, which leads to poor solubility and cell permeability.

### Folate-caged PROTACs

Folate-caged PROTACs is another technology that can improve the targeting specificity of PROTACs. Its basic principle is similar to Antibody-PROTAC, which introduces folate groups into PROTAC molecules to achieve release in targeted cells and tissues. Since folate receptor α (FOLR1) is low in normal tissues but is highly expressed in many human cancers, including multiple myelom (MM), lymphoma, and non-small cell lung cancer (NSCLC), folate-conjugating strategy is one of the commonly used drug delivery methods. This strategy has been widely and maturely used in tumor imaging and cancer-targeted drug delivery. At the same time, several FOLR1 targeted drugs have good antitumor effects and are currently in phase II/III clinical trials.^527–534^ Therefore, the use of folate-conjugating strategy in PROTACs technology (folate-caged PROTACs) to achieve the specific delivery of degraders to cancer cells has become a practical method. In this technology, folate releases active PROTAC by the action of cell endogenous hydrolase, and then the degrader induces the degradation of the target protein (POI). This strategy can eliminate the potential toxicity of degraders in normal tissues.

**Fig. 85:**
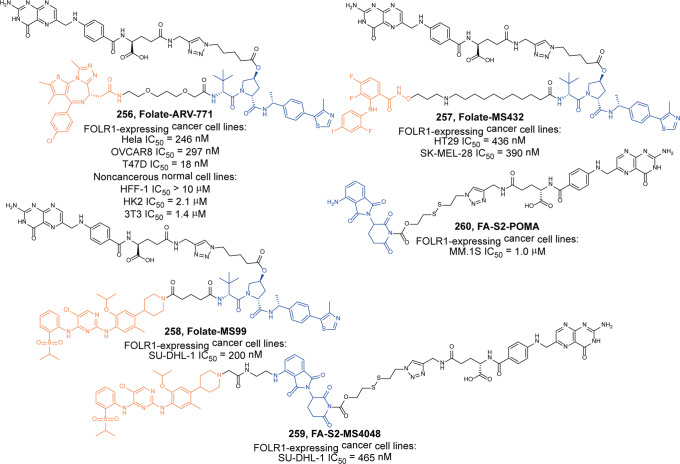
The representative PROTACs of Folate-Caged PROTACs

In 2021, Wei and Jin group reported the first folate-caged PROTAC. To ensure that the design of folate-caged PROTAC was universally applicable, they introduced a folate group on the E3 ubiquitin ligase ligand.^535^ Based on the reported BRD protein degrader **ARV-771**, they combined folate to the hydroxyl group of VHL via an ester bond to obtain folate-caged PROTAC **256** (**Folate-ARV-771**, Fig. 85). Subsequently, they tested the degradation activity of the folate-caged PROTAC **256** (**Folate-ARV-771**) on BRD4 protein in different cell lines and found that it could introduce the degradation of BRD4 protein effectively, which was equivalent to the degrader **ARV-771** in cells that highly express folate, when it also has shown similar antiproliferative activity as **ARV-771**. Meanwhile, as the expression of folate was less in normal cells, the degradation activity of BRD4 protein and antiproliferative activity were weaker than that of degrader **ARV-771**. Those experiments have proved that folate-caged PROTAC could exert high-efficiency degradation activity and anti-proliferation activity in tumor cells with high folate expression while its activity was weak in normal cells, which has achieved the purpose of targeting specific cells to generate degradation activity.

In addition, they also designed other folate-caged PROTAs that target other proteins in order to verify the versatility of the method. They designed folate-caged PROTAC **257** (**Folate-MS432**, Fig. 85) which was based on the MEK protein degrader **MS432**, **258** (**Folate-MS99**, Fig. 85), and **259** (**FA-S2-MS4048**, Fig. 85) which were based on the ALK protein degrader **MS99** and **MS4048.**^536^ And then it was also found that the folate-caged PROTACs have similar properties to that of **Folate-ARV-771**. They could efficiently introduce the degradation of the target proteins and have strong antiproliferative activity in high-expression-folate cell lines whereas having little effect on the target protein in normal cells.

Subsequently, they reported the first case of folate-caged molecular glue **260** (**FA-S2-POMA**, Fig. 85) that was designed and synthesized based on pomalidomide. They found that in cell lines that highly express folate, the molecular glue **260** (**FA-S2-POMA**) could efficiently induce the degradation of IKZF3, but its antiproliferative activity (IC_50_ = 1.0 µM) on MM.1S cells was weaker than that of pomalidomide (IC_50_ = 58 nM), which may be caused by the incomplete release of the molecular glue caged by folate in the cells.

In summary, they designed a universal folate-caged PROTAC design platform, through which they proved that the strategy could be applied to the protection and targeted delivery of PROTACs and molecular glues. This strategy could realize the selection of degraders for cancer cells and the safety for normal cells, and provide a reference method for avoiding the potential toxicity of protein degraders.

### TF-PROTACs

Transcription factors (TFs) are a class of proteins that are related to gene expression and regulation.^537^ In addition to normal regulatory functions, the cancer dependency map project (DepMap) also finds that TFs are also a class of essential proteins that maintain cancer cell proliferation and tumorigenesis, thus indicating that TFs are potential targets for tumor therapy.^538^ There are about 1600 TFs discovered and they can be divided into more than a dozen families according to their functions and structures. These TFs are different from traditional kinases as they do not have active pockets or allosteric regulatory site commonly found in kinases or other enzymes, so they are difficult to be targeted by small-molecule inhibitors.^539^ Among the reported TFs, only a few have small-molecule inhibitors, including NF-κB,^540,541^ STAT3/5,^542–544^ MYC,^545,546^, and nuclear receptors AR^547,548^ and ER.^549^ Most other TFs cannot be effectively targeted by small-molecule inhibitors, so the development of TFs small-molecule inhibitors is extremely difficult.

Since TFs can bind to specific DNA sequences and regulate the transcription process, different DNA sequences can be used instead of small-molecule inhibitors to target TFs and regulate their biological functions in theory. At present, researchers have experimentally determined more than 600 DNA sequences that can specifically bind to human TFs, which provides great possibilities for the targeted regulation of TFs. Inspired by the PROTAC technology, researchers are thinking about whether the small-molecule ligands that target the protein in the PROTAC can be replaced with the corresponding DNA sequence, so that it can form a TF-PROTAC to target specific TFs and induce their degradation to regulate the level of specific TFs and biological functions.

**Fig. 86:**
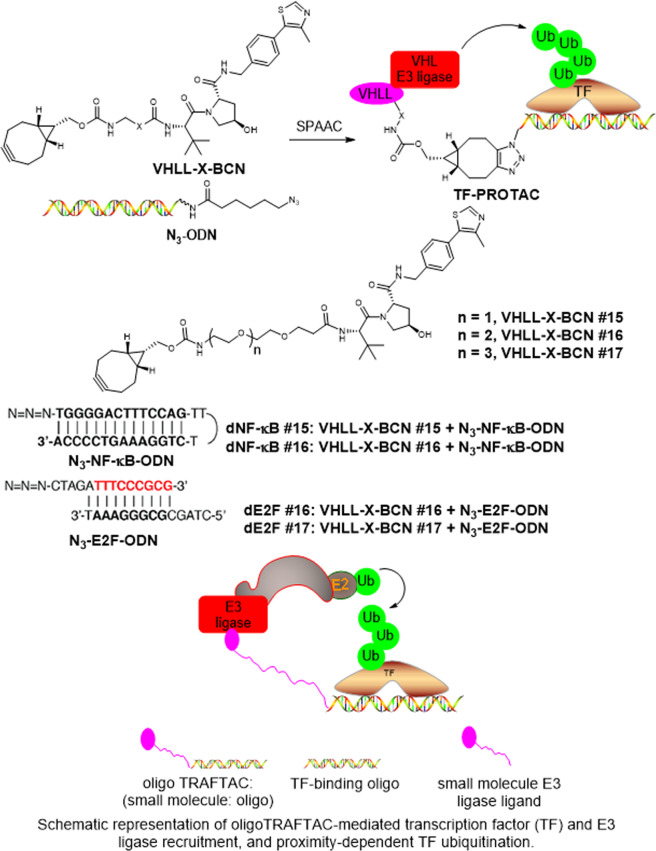
The representative PROTACs of TF-PROTACS

In 2021, Wei group reported a platform named TF-PROTAC,^550^ which linked DNA sequences to E3 ligase ligands via a copper-free strain-promoted azide-alkyne cycloaddition (SPAAC) reaction to selectively induce the degradation of the interested TFs (Fig. 86). They used NF-KB as the first TF to be degraded and designed a series of BCN-modified VHL ligands. At the same time, they generated a single strand DNA oligonucleotide, 5′-TGGGGACTTTCCAGTTTCTGGAAAGTCCCCA-3′ (hereafter termed as NF-κB-ODN)^551^ that specifically bind to NF-KB, then modified it by azide and obtained **N**_**3**_**-NF-κB-ODN**. Afterward, they tested the binding of BCN-modified VHL ligands to the modified **N**_**3**_**-NF-κB-ODN** in cells and found that most VHL ligands could interact well with **N**_**3**_**-NF-κB-ODN**. By having tested the degradation activity of different VHL ligands to p65 (a subunit of NF-KB) in HeLa cells, they found that **dNF-κB #15** and **dNF-κB #16** showed the best activity to induce the degradation of p65 and had good antiproliferative activity in tumor cells.

Moreover, they used this technology to design a TF-PROTAC that induced the degradation of E2F. Similar to the above method, they selected a double-strand DNA, in which the sense chain was 5′-CTAGATTTCCCGCG-3′ and the antisense chain was 5′-CTAGCGCGGAAAT-3′ (hereafter named as **E2F-ODN**)^552^ that targeted E2F and modified it by azide to obtain **N**_**3**_**-E2F-ODN**. Then, the degradation activities of different VHL ligands to E2F were tested in HeLa cells, and it was found that **dE2F #16** and **dE2F #17** showed the best E2F degradation activity and also had good antiproliferative activity in tumor cells. This was the first report of the technology for targeted degradation of TFs. The experimental results have shown this platform is a universal technology for inducing the degradation of TFs and that it has overcome the shortcomings of traditional small-molecule inhibitors and thus provided a possible solution to target the undruggable TFs.

In 2021, Crews group developed a novel targeted TFs degradation technology “oligoTRAFTACs”^553^ (Fig. 86), which is based on the binding of oligonucleotide sequences to TFs, the binding of E3 ligase ligands to E3 ligase, and the ubiquitination degradation system in vivo. This technology requires the generation of a chimeric oligoTRAFTAC via a copper-catalyzed alkyne-azide cycloaddition (CuAAC) click reaction between an alkyne-oligonucleotide and an azide-containing VHL ligand, which then acts on the target TFs, resulting in the degradation of a transcription factor of interest. In this study, they successfully induced the degradation of c-Myc and brachyury using two different oligoTRAFTACs, and also demonstrated the feasibility of applying oligoTRAFTACs to degrade brachyury in vivo in zebrafish.

Apart from the new technologies based on PROTAC described above, researchers have also developed a series of other new technologies, such as BioPROTACs,^554^ Covalent PROTACs,^555^ Photocaged PROTACs,^556^ Pre-fused PROTACs,^557^ RNA-PROTAC,^558–560^ Semiconducting polymer nano-PROTACs,^561^ Trivalent PROTACs^212,562^, and so on. These innovations can not only induce the degradation of target proteins but also induce the degradation of RNA, make up for the related shortcomings of traditional PROTAC and achieve the control of PROTAC on space and time scales. They have been well applied in a variety of cells, and laid the foundation for the development and maturity of PROTAC technology. Since having been reviewed in literatures, these technologies are only briefly discussed in this article.

## PROTACs in clinical trials

In addition to research tools, PROTACs also have great potential applications in disease treatment. It has become a new mode of drug discovery, which has the potential to change traditional drug discovery and may become a new blockbuster therapy.

Update to March 2022, about a dozen PROTACs around the world have entered the clinical development stage (Table 7). Among them, Arvinas’ **ARV-110** and **ARV-471** have entered clinical phase II stage, which are the fastest clinical progress in PROTAC drugs. Some R&D start-ups have gained the attention of large global pharmaceutical companies such as Roche, Sanofi, Merck, Pfizer, Gilead, etc. In addition, Chinese companies have also actively participated in the development, including BeiGene, Kintor, Haisco, Nuocheng Jianhua, Ascentage, Hengrui, etc.

**Table 7:** The summary of protein-degradation drug candidates based on PROTAC technology in the global clinical and IND stages (data source: https://clinicaltrials.gov updated: 3/20/2022)

NO.	Time	Company	PRORAC	Target	Indications	E3Ligase	Phase	Nation
1	2019	Arvinas	**ARV-110**	AR	Metastatic Castration Resistant Prostate Cancer	CRBN	Phase II	America
2	2019	Arvinas Pfizer	**ARV-471**	ER	ER+/HER2- Locally Advanced or Metastatic Breast Cancer	CRBN	Phase II	America
3	2020	Bristol Myers Squibb	**CC-94676**	AR	Metastatic Castration- Resistant Prostate Cancer	CRBN	Phase I	America
4	2021	BeiGene	**BGB-16673**	BTK	B-Cell Malignancies	CRBN	Phase I	China
5	2021	Nurix Therapeutics	**NX-2127**	BTK	Relapsed/Refractory B-cell Malignancies	CRBN	Phase I	America
6	2021	Nurix Therapeutics	**NX-5948**	BTK	Relapsed/Refractory B-cell Malignancies	CRBN	Phase I	America
7	2021	Haisco	**HK29116**	BTK	Relapsed/Refractory B-cell Malignancies	CRBN	Phase I	China
8	2021	Lynk	**LNK-01002**	Ras GTPase	Primary (PMF) or Secondary Myelofibrosis (PV-MF, ET-MF) or Acute Myeloid Leukemia	CRBN	Phase I	China
9	2021	Accutar Biotech	**AC682**	ER	Locally Advanced or Metastatic ER+ Breast Cancer	CRBN	Phase I	America
10	2021	Arvinas	**ARV-766**	AR	Metastatic Castration-Resistant Prostate Cancer	Undisclosed	Phase I	America
11	2021	Foghorn Therapeutics	**FHD-609**	BRD9	Advanced Synovial Sarcoma	Undisclosed	Phase I	America
12	2021	Kymera Sanofi	**KT-474**	IRAK4	Atopic Dermatitis (AD) or Hidradenitis Suppurativa (HS)	Undisclosed	Phase I	America
13	2022	Hinova	**HP518**	AR	Metastatic Castration-Resistant Prostate Cancer	CRBN	Phase I	China
14	2022	Kintor	**GT-20029**	AR	Prostate Cancer	CRBN	Phase I	China
15	2022	Arvinas	**ARV-110**	AR	**ARV-110** in Combination with **Abiraterone**	CRBN	Phase I	America
16	2022	Kymera	**KT-413**	IRAK4	Relapsed or Refractory B-cell NHL	CRBN	Phase I	America
17	2022	Kymera	**KT-333**	STAT3	Refractory Lymphoma, Large Granular Lymphocytic Leukemia, Solid Tumors	Undisclosed	Phase I	America
18	2022	C4 Therapeutics	**CFT8634**	BRD9	Synovial Sarcoma	CRBN	IND	America
19	2022	C4 Therapeutics	**CFT8919**	EGFR- L858R	Non-small-cell Lung Cancer	CRBN	IND	America
20	2022	Cullgen	**CG001419**	TRK	Cancer and other indications	CRBN	IND	America

Based on the application time of clinical trials, there are two applications in 2019, one in 2020, nine in 2021, and eight in 2022. It can be seen that clinical applications are currently in an explosive growth mode. In the future, more and more companies will participate in clinical trials of PROTAC-related drugs.

Based on the targets in clinical trials, AR has the most examples by six, BTK is followed by four, ER and BRD9 are two, respectively, and all the other remaining targets are one. It can be seen that currently pharmaceutical companies are more inclined to targets with relatively high maturity.

Today, PROTAC technology has become a new strategy for new drug research and development, providing new methods for the treatment of diseases. The next few years will be a critical period for the development of PROTACs. More and more PROTACs will enter preclinical and clinical research to further test the therapeutic effect of PROTACs. It is expected that PROTAC technology will provide benefits for human disease treatment and life health in the future.

## Conclusion and perspectives

Since the first case of PROTAC was reported in 2001, PROTAC technology has entered the stage of practical application from concept. Although the field of PROTAC has been rapidly developed in the past 20 years, there are still many challenges to be solved. These challenges mainly come from two aspects, namely PROTAC molecular design and optimization of druggability, and comprehensive evaluation of biological activity.

The first is about the molecular design and druggability of PROTAC, involving target protein ligands, new E3 ligase ligands, and new linking chains.

1. Design of target protein ligand. Most of the PROTAC molecules that have been reported are kinase degraders, and there are few examples of targeting undruggable targets. PROTAC molecules that target kinases are usually obtained by using existing small-molecule inhibitors as target protein ligands for modification. These inhibitors are designed for target kinase binding pockets. For undruggable targets, including transcription factors, phosphatases, protein–protein interactions, etc., the overall progress is slow due to the lack of effective small-molecule ligands.
2. New E3 ligase ligand. There are still few E3 ligases that can be used in PROTAC design. How to expand the E3 ubiquitin ligases that can be used in PROTAC technology is also one of the challenges that PROTAC faces.
3. How to connect POI and E3 ligase ligand. Researchers have realized that the linkers can profoundly affect the activity, selectivity, and druggability of PROTAC molecules. How to efficiently design and connect POI and E3 ligase ligands is also an important issue.
4. Pharmacological properties. The PROTAC molecule is usually not good enough as a drug due to its large molecular weight. How to optimize quickly is a huge challenge.
5. PROTAC molecules generally have a “hook” effect. Can a reasonable molecular design be used to weaken or even eliminate this concentration-dependent problem? Obviously, conventional medicinal chemistry screening and optimization methods are not suitable for the rapid development and optimization of PRTOAC molecules, especially for undruggable targets.

Aiming at the above-mentioned problems, AI technology (protein structure prediction), virtual drug screening technology and DEL screening technology, etc. can help develop the corresponding ligands for the target protein and E3 ligase. Based on these screened ligands, it is necessary to develop efficient synthesis methods to quickly and effectively construct large-scale PROTAC molecular libraries characterized by skeletal diversity for high-throughput screening and optimization of molecular druggability. In terms of molecular design, the existing ternary complex structures of PROTAC molecule with POI and E3 ligase proteins are still very few. In the future, more complex information obtained by X-Ray or cryo-electron microscopy will help in better molecular design. In recent years, breakthroughs in the ability to predict the protein and related complex structure by alphafold2 may also contribute to the design of PROTACs. For molecular optimization, increasing the overall rigidity of the molecule and reducing the molecular weight can generally improve the drug-like properties of the hit compounds, such as oral bioavailability, ADME, etc.

The second is about biological activity evaluation. This involves the screening of PROTAC molecules, evaluation of druggability, and pharmacological evaluation.

1. PROTAC molecular screening. The existing technical methods mainly rely on immunoblotting methods and proteomics methods, which are not only time-consuming, labor-intensive, low-efficiency, and high-cost. In recent years, screening technologies such as fluorescent tags and HiBiT have been gradually introduced. In the future, more new high-throughput and high-sensitivity methods are needed for rapid and accurate assessment.
2. Evaluation of druggability. In addition to the conventional evaluation indicators such as solubility, in vitro and in vivo activity, toxicity, and other drug-like properties. The role of PROTAC is to catalyze the cycle, so traditional methods cannot accurately assess the properties of PROTAC’s PK and PD. For PROTAC molecules, there is a great need to develop PK/PD models that are more consistent with protein degradation as a new drug modality.
3. How to better understand the degradation activity or degradability, selectivity, possible off-target effects, and pharmacological effects of PROTAC molecules (based on different targets, different cell lines, and different animal models). And how to correspondingly achieve differentiation in clinical treatment.

None of these discussed questions currently have ready-made answers, but we believe that as more and more research progresses, the whole field will be greatly advanced. With the development of more biological, pharmacological, and clinical research, new evaluation methods and systems will gradually be set up to solve these problems. It is believed that in the future, more and more PROTAC molecules will not only be used as tools for basic biological research but will also enter the clinic to solve the actual needs of patients.
